# Urinary Proteome and Exosome Analysis Protocol for the Discovery of Respiratory Diseases Biomarkers

**DOI:** 10.3390/biom15010060

**Published:** 2025-01-03

**Authors:** Laura Martelo-Vidal, Sara Vázquez-Mera, Pablo Miguéns-Suárez, Susana Belén Bravo-López, Heidi Makrinioti, Vicente Domínguez-Arca, Javier de-Miguel-Díez, Alberto Gómez-Carballa, Antonio Salas, Francisco Javier González-Barcala, Francisco Javier Salgado, Juan José Nieto-Fontarigo

**Affiliations:** 1 BioLympho Research Group, Department of Biochemistry and Molecular Biology, Faculty of Biology-Biological Research Centre (CIBUS), Universidade de Santiago de Compostela, 15782 Santiago de Compostela, Spain; laura.martelo@rai.usc.es (L.M.-V.); sara.vazquez.mera@rai.usc.es (S.V.-M.); pablo.miguens@rai.usc.es (P.M.-S.); francisco.javier.gonzalez.barcala@sergas.es (F.J.G.-B.); juanjose.nieto.fontarigo@usc.es (J.J.N.-F.); 2 Translational Research in Airway Diseases Group (TRIAD), Health Research Institute of Santiago de Compostela (IDIS), 15706 Santiago de Compostela, Spain; 3 Proteomic Service, Health Research Institute of Santiago de Compostela (IDIS), 15706 Santiago de Compostela, Spain; susana.belen.bravo.lopez@sergas.es; 4 Department of Emergency Medicine, Massachusetts General Hospital, Harvard Medical School, Boston, MA 02114, USA; cmakrynioti@mgh.harvard.edu; 5 Grupo de Física de Coloides y Polímeros, Departamento de Física de Partículas, Universidade de Santiago de Compostela, 15782 Santiago de Compostela, Spain; vdominguez@iim.csic.es; 6 Bioprocess Engineering Group, Instituto de Investigacións Mariñas (IIM-CSIC), 36208 Vigo, Spain; 7 Respiratory Department, Hospital General Universitario Gregorio Marañón, 28009 Madrid, Spain; javier.miguel@ucm.es; 8 Health Research Institute Gregorio Marañón (IISGM), 28009 Madrid, Spain; 9 Faculty of Medicine, Universidad Complutense de Madrid, 28040 Madrid, Spain; 10 Genetics, Vaccines and Infections Research Group (GenViP), Instituto de Investigación Sanitaria de Santiago, Universidade de Santiago de Compostela, 15706 Santiago de Compostela, Spain; alberto.gomez.carballa@sergas.es (A.G.-C.); antonio.salas@usc.gal (A.S.); 11 Unidade de Xenética, Instituto de Ciencias Forenses, Facultade de Medicina, Universidade de Santiago de Compostela, 15782 Santiago de Compostela, Spain; 12 Genética de Poblaciones en Biomedicina (GenPoB) Research Group, Instituto de Investigación Sanitaria (IDIS), Hospital Clínico Universitario de Santiago (SERGAS), 15706 Santiago de Compostela, Spain; 13 Centro de Investigación Biomédica en Red de Enfermedades Respiratorias (CIBER-ES), 28029 Madrid, Spain; 14 Department of Respiratory Medicine, University Hospital Complex of Santiago de Compostela, 15706 Santiago de Compostela, Spain; 15 Department of Medicine, Universidade de Santiago de Compostela, 15782 Santiago de Compostela, Spain

**Keywords:** exosomes, miRNAs, proteomics, respiratory disease biomarkers, urine

## Abstract

This study aims to develop a protocol for respiratory disease-associated biomarker discovery by combining urine proteome studies with urinary exosome components analysis (i.e., miRNAs). To achieve this, urine was DTT treated to decrease uromodulin, then concentrated and ultracentrifuged. Proteomic analyses of exosome-free urine were performed using LC-MS/MS. Simultaneously, miRNA expression from urine exosomes was measured using either RTqPCR (pre-amplification) or nCounter Nanostring (non-amplication) analyses. We detected 548 different proteins in exosome-free urine samples (N = 5) with high confidence (FDR < 1%), many of them being expressed in different non-renal tissues. Specifically, lung-related proteins were overrepresented (Fold enrichment = 1.31; FDR = 0.0335) compared to whole human proteome, and 10–15% were already described as protein biomarkers for several pulmonary diseases. Urine proteins identified belong to several functional categories important in respiratory pathology. We could confirm the expression of miRNAs previously connected to respiratory diseases (i.e., miR-16-5p, miR-21-5p, miR-146a-5p, and miR-215-5p) in urine exosomes by RTqPCR. Finally, we detected 333 miRNAs using Nanostring, 15 of them up-regulated in T2^high^ asthma (N = 4) compared to T2^low^ asthma (N = 4) and healthy subjects (N = 4). Therefore, this protocol combining the urinary proteome (exosome free) with the study of urinary exosome components (i.e., miRNAs) holds great potential for molecular biomarker discovery of non-renal and particularly respiratory pathologies.

## 1. Introduction

Urine is the most common source of biomarkers for renal, bladder, or prostate pathologies [1]. In addition, this biofluid derives directly from plasma; hence, its composition can reflect changes associated with disease state in tissues far from the renal system. Thus, urine is naturally enriched in components that cross the glomerular filtration barrier, such as low molecular weight proteins [2].

Interestingly, the utility of urine was previously demonstrated using different -omic approaches. For example, some proteomic studies have evidenced changes in specific proteins associated with different non-renal pathologies, including brain (e.g., Alzheimer’s disease, Parkinson’s disease) and cardiovascular (e.g., coronary heart disease, obesity) disorders [3,4,5,6] or cancer (e.g., hepatocellular carcinoma, endometrial cancer) [7,8]. However, only a handful of studies utilizes this sample for respiratory diseases. In this respect, urine was specially studied using lipidomic and metabolomic approaches [9,10,11]. For instance, urine is an ideal sample for the study of eicosanoids, especially important in respiratory pathology [9,12]. However, the study of urine proteome associated with respiratory diseases was not extensively addressed. Indeed, to our knowledge, there are no works assessing the urinary proteome of patients with asthma or COPD.

Both asthma and COPD, main chronic respiratory diseases, are responsible for the 3.79% of total disability-adjusted life-years (DALYs), whereas only COPD represent 7.03% of death causes in the world [13]. They are also highly heterogeneric diseases, with several phenotypes and endotypes [14,15,16,17]. Thus, the discovery of biomarkers whose variation is associated with specific molecular pathways, physiological mechanisms, or pharmacological responses is key to categorize these patients and to develop personalized treatment strategies (i.e., precision medicine). In this regard, the use of non-invasive samples such as urine is urgently needed [18].

Another approach that has attracted a lot of interest in urine biomarker discovery is the analysis of exosomes. Exosomes are small vesicles (30–150 nm) produced by all types of immune and structural cells (including those from lung tissues) that reach the blood stream and could be finally detected in urine [19]. These vesicles transport different biomolecules (DNA, mRNA, miRNA, and proteins), whose levels can vary depending on the cell of origin and the presence of a pathology; therefore, exosomes can be used as a potential source of molecular biomarkers. For example, several miRNAs were associated with specific respiratory disorders, including asthma (e.g., miR-21-5p, miR-126-3p, miR-146a-5p, miR-2155p, miR-148b-3p, or miR-221-5p) [20,21], COPD (e.g., miR-223-3p) [22], or respiratory infections (e.g., miR-146a-5p and miR-155-5p) [23]. These works were mainly carried out in serum or tissue samples [24], but hardly any were carried out in urine.

Taken together, this clearly exposes a need to develop novel high-throughput biomarker discovery strategies for non-renal and particularly respiratory pathologies by using urine as a non-invasive and a clinically relevant sample. In this study, we have established a protocol combining the analysis of the urinary proteome with the analysis of exosomal miRNA cargo that provides different layers of information and increases the potential for discovering molecular biomarkers of non-renal diseases. This is particularly interesting for heterogeneous respiratory diseases such as asthma or COPD, in which there are no studies in humans.

## 2. Material and Methods

### 2.1. Study Population

One hundred mL of second-void urine was collected from 5 adult (30–69 years old) healthy volunteers (4 male/1 female) recruited between 1 September 2021 and 31 January 2022. Donors were scheduled for minor therapeutic interventions (orthopedic surgery or inguinal hernia.

For multiplex miRNA expression analyses, 50 mL of second-void urine was used from 12 donors: 4 healthy subjects, 4 T2^high^ asthma donors, and 4 T2^low^ asthma donors. All patients had stable asthma (≥3 months), were adults (54–72 years old), with a similar age/sex ratio and an asthma diagnosis according to GINA criteria 2017 (https://ginasthma.org/). T2^high^ asthma was defined as blood eosinophil count (BEC) ≥ 300 cells/mL, fractional exhaled nitric oxide (FeNO) ≥ 30 ppb, and IgE ≥ 100 UI/mL.

The study protocol was approved by the Ethics Committee of Research Santiago-Lugo (2019/601). All donors have been informed and signed a written consent to participate in the study. All methods described below were performed according to the relevant guidelines and regulations, as well as the Declaration of Helsinki.

### 2.2. Urine Processing

**Figure 1:**
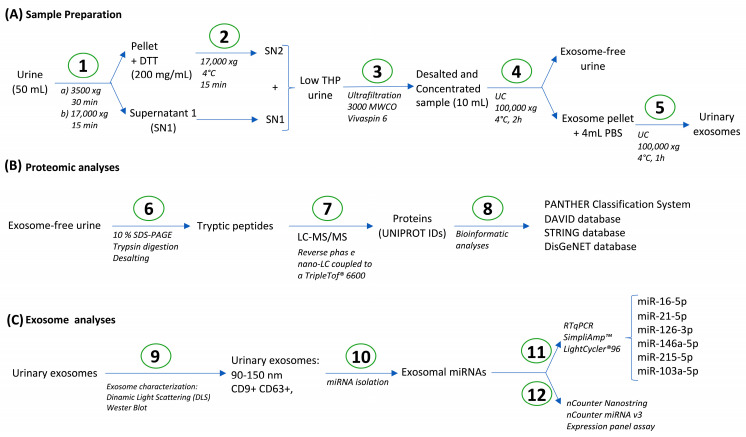
Study design. (**A**) Sample preparation, including sample cleaning (1), DTT treatment for uromodulin (THP) depletion (2), ultrafiltration for desalting and concentration (3), and ultracentrifugation for exosome isolation (4–5). (**B**) Exosome-free urine proteomic analyses using LC-MS/MS (6–8). (**C**) Analyses of respiratory-related exosomal miRNAs in human urine (9–10) by RTqPCR (11) (miR-16-5p, miR-21-5p, miR-126-3p, miR-146a-5p, miR-215-5p, miR103a-5p) and multiplex miRNA analysis (nCounter Nanostring; 12).

The study design is depicted in Figure 1. Firstly, an aliquot of urine (50 mL) from each patient was centrifugated (3500× *g*, 30 min, 4 °C) to remove cells and cell debris. This precleaned sample was then centrifuged (17,000× *g*, 15 min, 4 °C) to reduce the amount of uromodulin (THP). The supernatant was collected and transferred into a new tube. The pellet (enriched in THP) was treated with dithiothreitol (DTT) (final concentration of 200 mg/mL) for 30 min at 35 °C to avoid losing important components, such as exosomes [25]. Then, it was centrifuged again (17,000× *g*, 15 min, 4 °C), and the resulting supernatant was combined with the previous one. THP-rich pellets were discarded (Figure 1A).

Low-THP urine samples were subsequently ultrafiltered (UF) (4000× *g*, RT) using 3000 MWCO devices (Vivaspin^®^ 6, 3000 MWCO, PES; Sartorius, Göttingen, Germany) for desalting and concentration. The concentrated volume (~10 mL) was ultracentrifuged (UC) (100,000× *g*, 2 h, 4 °C) (Optima L-70 Ultracentrifuge; Beckman, Brea, CA, USA). Exosome-free supernatant was obtained and stored until proteomic analysis. Exosome-rich pellet was washed with 4 mL of PBS and ultracentrifuged again (100,000× *g*, 4 °C, 1 h). The resulting pellet, containing urinary exosomes, was resuspended in final volume of 250 µL in PBS and stored at −80 °C until further analysis (Figure 1A).

### 2.3. Urinary Proteome Analysis

To perform a proteomic quantification using gel electrophoresis–liquid chromatography mass spectrometry (LC-MS/MS) techniques, exosomes were lysed and the protein was quantified to analyze equal amounts of μg per sample (Figure 1B). Extensive information about protein quantification and proteomic quantification are detailed in the Supplementary Methods [26,27,28,29,30,31,32].

### 2.4. Bioinformatic Analysis

Gene Ontology (GO) analysis and overrepresentation tests were performed using PANTHER (PANTHER Classification System; V17.0, Released 22 February 2022, Accessed on 11 July 2022). Functional annotation clustering for GO-Biological Processes and reactome pathways, and tissue expression distribution of proteins were performed using The Database for Annotation, Visualization and Integrated Discovery (DAVID; Released 15 June 2022; Accessed on 11 July 2022; DAVID Knowledgebase v2022q2 released, https://davidbioinformatics.nih.gov/content.jsp?file=update.html). Additionally, protein–protein interaction networks were made with the STRING database (v.10.0 database, free access at http://string-db.org). The DisGeNET database “DisGeNET-a database of gene-disease associations” (https://www.disgenet.org/) was used to identify already described biomarkers of disease (Figure 1B).

### 2.5. Exosome Characterization

Exosome characterization was performed using size distribution by dynamic light scattering (DLS) and measuring CD9 and CD63 levels by Western blot (Figure 1C). More details are provided in Supplementary Methods.

### 2.6. microRNA (miRNA) Isolation from Urine Exosomes—RT-qPCR

Exosomes were lysed and miRNA isolated using an miRNeasy Mini Kit (Qiagen, Hilden, Germany) switching lysis reagent for TRIZOL-LS (Invitrogen, Waltham, MA, USA). After that, RT-qPCR was performed using the miRCURY LNA RT and miRCURY SYBR Green PCR kits (Qiagen) and different primers (Figure 1C). More detailed information is provided in Supplementary Methods.

### 2.7. Multiplex miRNA Expression Analysis

We used the commercial nCounter^®^ miRNA v3 Expression Panel assay (https://nanostring.com/products/ncounter-assays-panels/immunology/mirna/; accessed on 11 July 2022) to quantify miRNA expression (Figure 1C). This panel allows for the simultaneous quantification of 827 biologically relevant miRNAs. We used 3 µL of total RNA as input and followed standard conditions for both the ligation and hybridization steps. More detailed information is provided in Supplementary Methods.

## 3. Results

### 3.1. Tissue Distribution of the Proteins Identified in Exosome-Free Urine Samples

**Figure 2:**
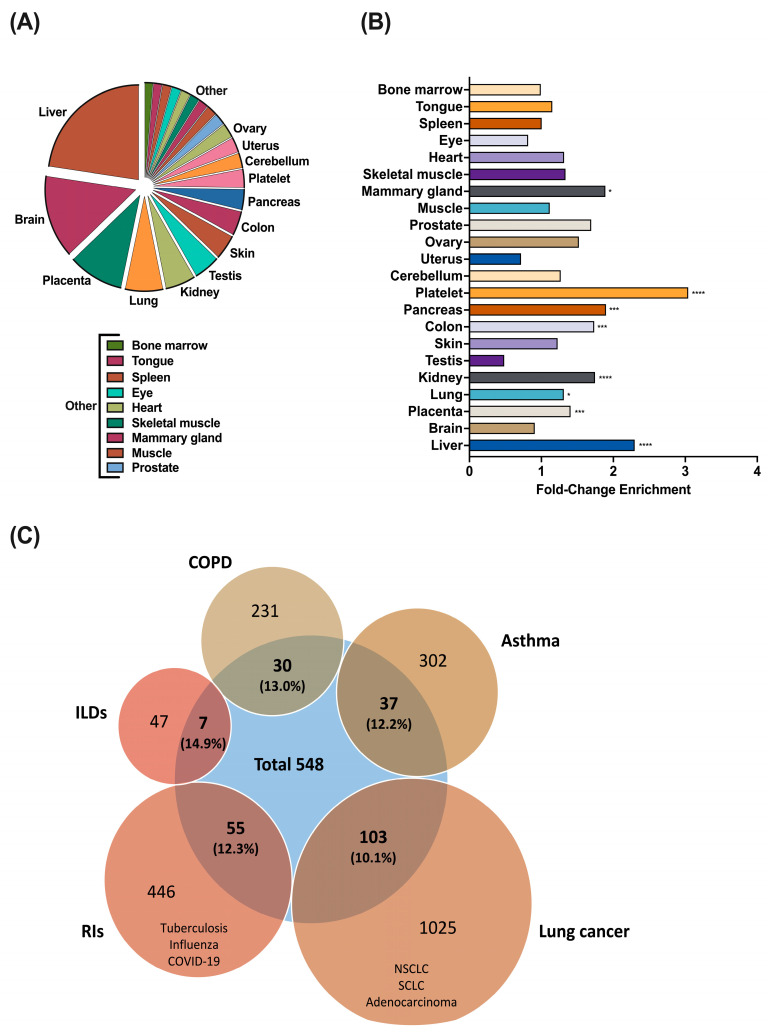
Expression of the proteins detected in urine in different tissues and respiratory diseases. (**A**) Tissue distribution of the proteins identified in urine excluding biofluids. (**B**) Enrichment of proteins detected in urine expressed in different tissues compared to the whole human proteome. (**C**) Biomarkers discovered (Disgenet database) in different pathologies related to the respiratory system. Number of disease biomarkers (and %) detected in urine with the protocol we developed. * *p* < 0.05; *** *p* < 0.001; **** *p* < 0.0001.

Using the protocol described in Figure 1, we identified 769 proteins with high confidence (FDR < 1%). Only those proteins found in at least two of the samples (N = 548) were selected for further analyses. Firstly, we aimed to study the tissue expression of these proteins. As expected, many of those proteins are expressed in biological fluids including plasma (N = 132, 24.4%; Fold enrichment (FE) = 16.65, FDR = 2.11 × 10^−127^), blood (N = 40, 7.10%; FE = 1.97, FDR = 0.0009), cerebrospinal fluid (N = 31, 5.67%; FE = 24.40, FDR = 3.30 × 10^−34^), saliva (N = 22, 4%; FE = 10.48, FDR = 2.46 × 10^−14^), bile (N = 20, 3.64%; FE = 32.92, FDR = 1.31 × 10^−25^), urine (N = 20, 3.63%; FE = 24.97, FDR = 1.13 × 10^−21^), milk (N = 16, 2.91%; FE = 17.55, FDR = 5.43 × 10^−14^), and serum (N = 13, 2.37; FE = 22.41, FDR = 8.82 × 10^−13^). Interestingly, many of the proteins we detected are expressed in tissues other than biological fluids (Figure 2A), including the liver (N = 324), brain (N = 202), placenta (N = 139), or lungs (N = 95). Moreover, there is an overrepresentation of proteins from the liver (FE = 2.29; FDR = 5.75 × 10^−64^), placenta (FE = 1.42; FDR = 0.0001), lungs (FE = 1.31; FDR = 0.0335), kidneys (FE = 1.75, FDR = 4.15 × 10^−5^), colon (FE = 1.73, FDR = 0.0006), pancreas (FE = 1.86; FDR = 0.0005), platelet (FE = 3.05; FDR = 3.48 × 10^−9^), and mammary gland (FE = 1.18; FDR = 0.0371) compared to the whole human proteome (Figure 2B), making urine an important source of biomarkers for diseases affecting these tissues.

Secondly, we have evaluated the ability of the developed proteomic protocol to discover biomarkers of lung disease. To do that, we have compared the detected urine proteome (N = 548) with the biomarkers previously described in different respiratory diseases included in the database “DisGeNET-a database of gene-disease associations” (https://www.disgenet.org/). The conditions included were asthma, COPD, and lung cancer (i.e., Non-Small Cell Lung Cancer/NSCLC, Small Cell Lung Cancer/SCLC, and Adenocarcinoma of the lung), Interstitial Lung Diseases (ILDs), and respiratory infections (RIs; i.e., Tuberculosis/TB, Influenza, and COVID-19) (Figure 2C). Of note, 10–15% of the already discovered protein biomarkers in respiratory diseases were identified in our study (Figure 2C). Particularly, we could detect 37/302 of the biomarkers of asthma, 30/231 of COPD, 103/1025 of lung cancer, 55/446 of Ris, and 7/47 of ILDs. A more detailed characterization of the biomarkers detected in urine with this protocol is provided in Supplementary Table S1.

### 3.2. Functional Characterization of the Proteins Identified in Urine

**Figure 3:**
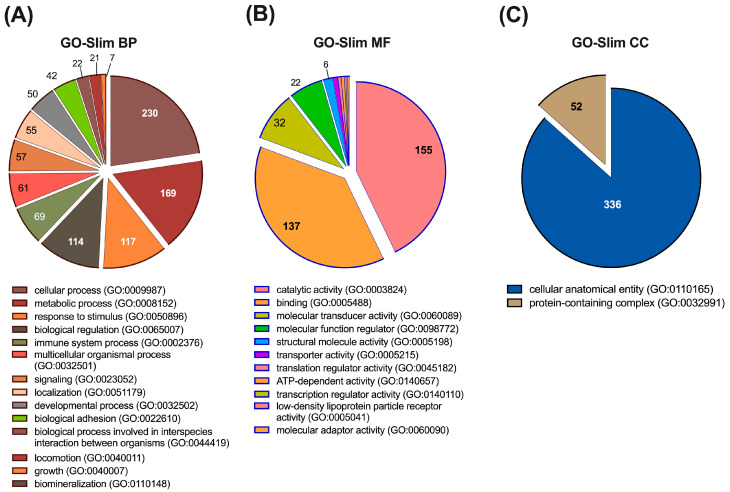
Functional classification of the detected proteins in Gene Ontology (GO) categories. The values on the graphs represent the number of proteins that belong to each GO category. Proteins with multiple GO annotations can be present in multiple GO categories. (**A**) GO-Slim Biological Process category (GO-Slim BP). (**B**) GO-Slim Molecular Function category (GO-Slim MF). (**C**) GO-Slim Cellular Component category (GO-Slim CC).

First, we made a qualitative analysis of the described urine proteome in terms of GO-term categories. Classification of the identified proteins (N = 548) in biological process categories (GO-Slim BP) indicates that 42.8% (N = 230) of them participate in cellular processes and 31.5% (N = 169) in metabolic processes. Interestingly from a biomarker discovery point of view, 21.8% (N = 117) of the proteins participate in response to stimulus, 21.2% (N = 114) in biological regulation, and 12.8% (N = 69) in immune system processes. We could also detect, for example, proteins implicated in signalling (10.6%; N = 57), localization (10.2%; N = 55), or biological adhesion (7.8%; N = 42) (Figure 3A). Classification by GO Molecular Function (GO-Slim MF) indicates that most of the identified proteins have catalytic activity (28.9%; N = 155) or binding functions (25.5%; N = 137), although other functions, including molecular transducer activity (6%; N = 32) and molecular function regulator activity (4.1%; N = 22), were also relevant (Figure 3B). Finally, classification by cellular component (GO-Slim CC) reveals that all proteins belong to cellular anatomical entity (62.6%; N = 336) or protein-containing complex (9.7%; N = 52) categories (Figure 3C).

Next, we performed a functional annotation clustering analysis using the DAVID database for GO-BP and reactome pathways. The similarity term overlap used was 3 and the similarity threshold 0.50. The multiple linkage threshold was set at 0.50, with an initial and final group membership of 3. The enrichment threshold was EASE = 1.0.

We identified 56 clusters with a positive enrichment score (Supplementary Table S2); all of them can be summarized into seven broader categories: Immune system, Haemostasis, Extracellular Matrix Organization/Cell adhesion, Signalling, Metabolism, Cell Death, and Others (Table 1). Each of these categories include several clusters (Table 1). Thus, the immune system category is made of ten clusters, including pathways with a significant enrichment score, such as innate (R-HSA-168249) and adaptive (GO:0002250) immune system, and complement cascade (GO:0006958, R-HSA-166658). The Haemostasis category is composed of four clusters with their representative pathways/processes being haemostasis (R-HSA-109582), fibrinolysis (GO:0042730), or blood coagulation (R-HSA-140837; GO:0072378). The next six clusters are related to proteins with a function in extracellular matrix organization or cell adhesion and include important and significantly enriched pathways like cell–cell communication (R-HSA-1500931), Nectin/Necl trans-heterodimerization (R-HSA-420597), or Degradation of Extracellular Matrix (R-HSA-1474228). The signalling category included eleven different clusters of proteins, but only three of them had an FDR < 0.05. Those three clusters are represented by the pathways Regulation of Insulin-like Growth Factor (IGF) transport and uptake by Insulin-like Growth Factor Binding Proteins (IGFBPs) (R-HSA-381426), Transmembrane receptor PTK signalling pathway (GO:0007169), and Ephrin signalling (R-HSA-3928664). Proteins with functions in metabolic processes and pathways are also overrepresented within the proteome detected. Thus, 16 clusters belong to this category, including relevant pathways involved in carbohydrate metabolism (R-HSA-5663084, R-HSA-71387, R-HSA-70326, R-HSA-189085, R-HSA-70268), lipid and lipoprotein metabolism (R-HSA-174824, GO:0006869, R-HSA-1660662), peptides and protein modifications (R-HSA-156590, R-HSA-159740, R-HSA-3781860), transcription (R-HSA-74160), metabolism of RNA (R-HSA-8953854), or detoxification of ROS (R-HSA-3299685). We have also described a category of proteins related to cell death, which participate in processes related to autophagy (R-HSA-9612973) or apoptosis (R-HSA-109581, GO:2000352). Finally, we also found proteins that participate in cell cycle (R-HSA-1640170), transport of gasses (R-HSA-1480926), developmental biology (R-HSA-1266738), and keratins (R-HSA-1638074).

### 3.3. Protein–Protein Interaction (PPI) Network Analysis of Proteins Identified in Urine

The final step was to perform a topological analysis of the proteins identified in urine. For this purpose, we used STRING with text mining, experiments, and databases as interaction sources. The interaction score was set as 0.700 (high confidence), and disconnected nodes in the network were omitted. In order to simplify the network, we ran Markov clustering (MCL) with an inflation value of 1.5. Candidate biomarkers with higher interactions could be more effective than proteins with fewer interactions. Only the first 15 clusters (out of 32) were depicted; all of them with PPI enrichment *p*-values < 1.0 × 10^−11^. The resulting network is easier to visualize and very simplified with the main interactions for the detected proteins; we have also included here the most important pathways enriched within the different clusters.

**Figure 4:**
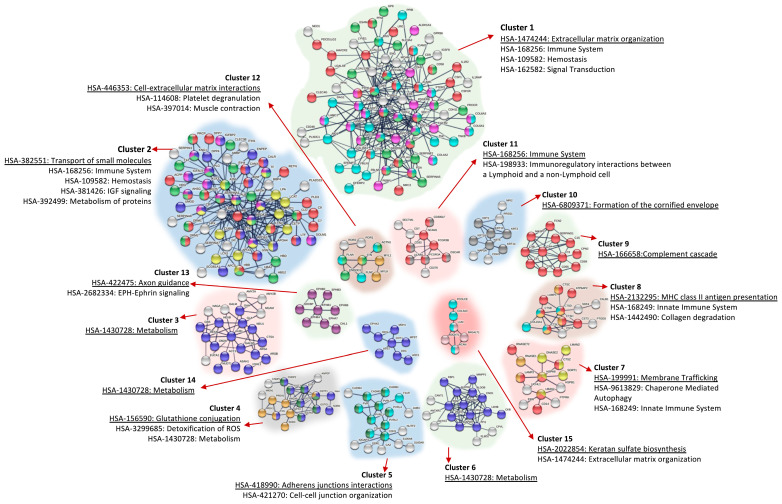
Protein–protein interaction (PPI) network of proteins identified in urine. STRING database PPI network. Interaction sources selected were text mining, experiments, and databases. The interaction score was set as 0.700 (high confidence), and disconnected nodes were omitted. Markov clustering (MCL) with an inflation value of 1.5 was used for clustering purposes. Only the first 15 clusters (out of 32) were depicted; all of them with PPI enrichment *p*-values < 1.0 × 10^−11^. Main reactome pathways under each cluster are highlighted.

As expected, the main pathways enriched in the different clusters are, in general, lines, the same as exposed in the previous section: immune system, haemostasis, metabolism, extracellular matrix organization, and signalling (Figure 4). Cluster 1 included 79 nodes and 248 edges, and the average (avg.) node degree was 6.28 and avg. local clustering coefficient 0.519; the expected number of edges was 18 and the PPI enrichment *p*-value was < 1.0 × 10^−16^. The most characteristic pathway enriched in this cluster was Extracellular Matrix Organization (HSA-1474244), but other important pathways are also included including immune system, haemostasis, and signal transduction (e.g., IGF signalling and signalling by receptor tyrosine kinases) (Figure 4). Clusters 5, 12, and 15 also included proteins with functions in extracellular matrix organization (Figure 4). Cluster 2, with 66 nodes, 190 edges, an avg. node degree of 5.76, and an avg. local clustering coefficient of 0.659 is made of proteins involved in different pathways, but with special influence of proteins with functions on transport of small molecules (HSA:382551). Proteins with similar function were also included in cluster 7 (avg node degree 2.29; avg. local clustering coefficient 0.626). Clusters 3 (avg. node degree 3.82; avg. local clustering coefficient 0.643), cluster 6 (avg. node degree 6.67; avg. local clustering coefficient 0.871), and cluster 14 (avg. node degree 2.00; avg. local clustering coefficient 0.679) are made of proteins involved in Metabolic Pathways (HSA:1430728). On the other hand, clusters 8, 9, and 11 included proteins with immune system functions. Cluster 4 (avg. node degree 4.33; avg. local clustering coefficient 0.655) included proteins with functions in glutathione conjugation and ROS detoxification; cluster 13 included the routes Axon guidance and EPH-Ephrin Signalling, and cluster 10 is mainly made of keratins (Figure 4).

### 3.4. Different miRNAs Previously Implicated in Respiratory Pathology Were Detected in Healthy Urinary Exosomes Using RTqPCR

As exposed in the study design (Figure 1C), the second part of the protocol we developed in the present study included the isolation of exosomes from urine and the analysis of representative miRNAs previously related to respiratory diseases in these samples using a pre-amplification technique (i.e., RTqPCR). This part was performed in parallel to the analysis of the urine proteome of exosome-free samples.

**Figure 5:**
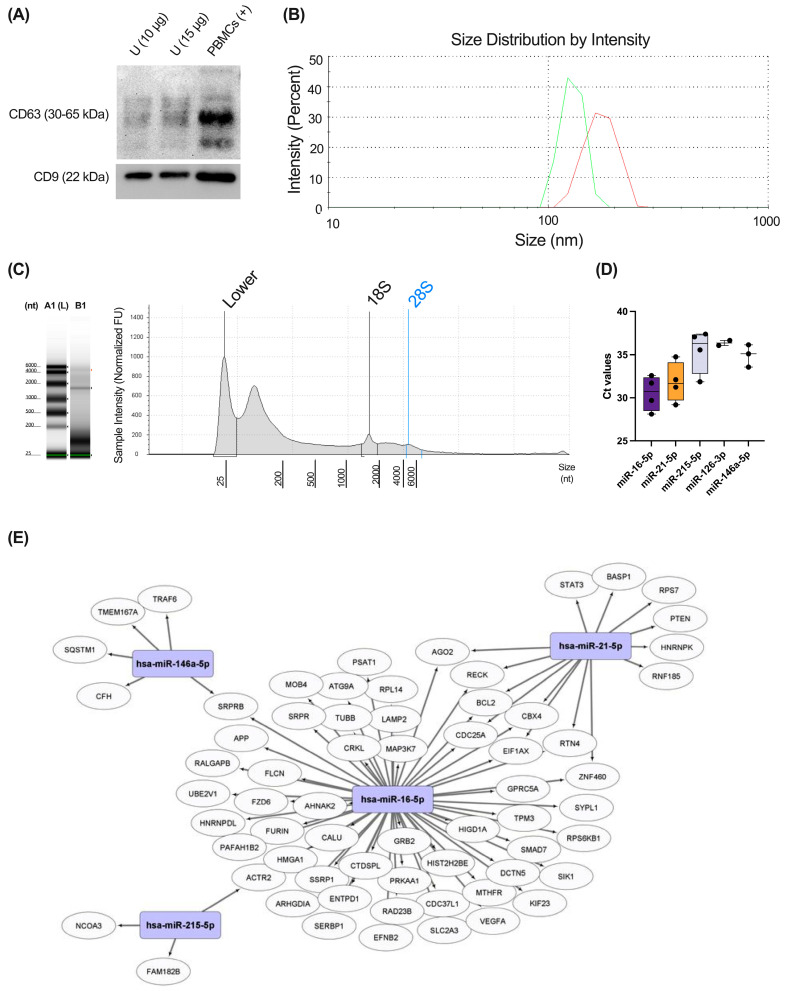
Analyses of urinary exosomal miRNAs previously related to respiratory pathology. (**A**) Expression of the typical exosomal markers CD9 and CD63 in exosomes isolated from urine, (western blot original images can be found in Supplementary Figure S1). (**B**) Size distribution of extracellular vesicles purified in urine samples using dynamic light scattering (DLS). A representative donor is depicted; two replicates (Shown in different colors). (**C**) High-Sensitivity RNA ScreenTape Assays of the isolated RNA from urinary exosomes. A representative donor is depicted. (**D**) RTqPCR analyses of miRNAs previously related to respiratory pathology (miR-16-5p. miR-21-5p. miR-215-5p. miR-126-3p. and miR-146a-5p). Ct values are depicted. N = 4. (**E**) mRNA targets for the different miRNAs studied (hsa-miR-16-5p, has-miR-21-5p, hsa-miR-146a-5p, and hsa-miR-215-5p) expressed in the respiratory system.

Exosomes isolated from four healthy donors were characterized using Western blot, and their size distribution was also confirmed by DLS using the Zetasizer Nano ZS instrument. Our results demonstrated the positivity for the typical exosomal markers CD9 and CD63 (Figure 5A). The presence of vesicles with the expected size of exosomes (90–150 nm) was also observed using DLS analysis (Figure 5B). RNA was isolated from urinary exosomes, and this sample was enriched in miRNAs (median size 25 nt), as demonstrated in High-Sensitivity RNA ScreenTape Assays (Figure 5C). Finally, using RTqPCR analyses, we measured the expression levels of representative miRNAs previously related to respiratory pathology: miR-16-5p, miR-21-5p, miR-126-3p, miR-146a-5p, and miR-215-5p (Figure 5D). The main targets of these miRNAs expressed in the respiratory system are depicted in Figure 5E.

As shown in Figure 5C, miR-16-5p was the highest expressed miRNA in urinary exosomes, followed by miR-21-5p. miR-215-5p was also detected in all samples used, but miR-126-3p and miR-146a-5p were only detected in two and three exosome samples, respectively. miR-103a-3p, a miRNA used as housekeeping in serum samples, was under the detection limit in urine, indicating the different composition of miRNAs in urinary exosomes compared to serum.

### 3.5. Several miRNAs from Urinary Exosomes Were Associated with Specific Molecular Phenotypes of Asthma Using a Multiplex miRNA Expression Assay

Lastly, we performed a proof-of-concept study using a multiplex miRNA expression assay (Nanostring nCounter technology) to characterize differentially expressed urinary exosome-miRNAs between healthy subjects and patients with different molecular phenotypes of asthma (i.e., T2^high^ and T2^low^) (Figure 1C). Characteristics of subjects included in the Nanostring analyses are depicted in Supplementary Table S3.

**Figure 6:**
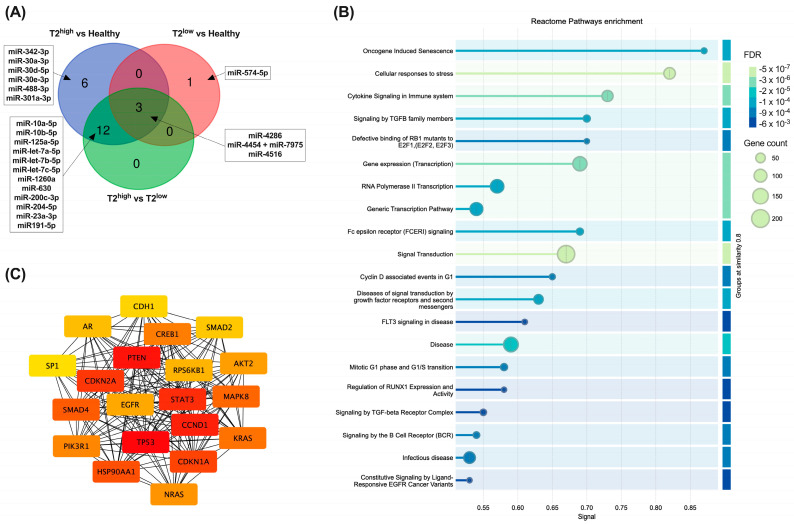
Analyses of urinary exosomal miRNAs using nCounter Nanostring technology. (**A**) Venn diagram of miRNAs with changes between T2^high^, T2^low^, and healthy subjects. (**B**) Reactome overrepresentation analyses of mRNA targets for miRNAs up-regulated in T2^high^ vs. T2^low^ and healthy subjects; the 20 most up-regulated pathways are depicted. (**C**) In total, 20 hub targets according to MCC topological properties of the miRNAs up-regulated in T2^high^ vs. T2^low^ and healthy subjects.

Using this non-amplification technique, we detected 333 miRNAs in urinary exosomes (Supplementary Table S4). Twenty-two miRNAs presented differential abundance among T2^high^, T2^low^, and healthy subjects (Figure 6A; Table 2). Fifteen miRNAs were enriched in T2^high^ compared to T2^low^ and healthy controls (Figure 6A; Table 2). Then, we searched for miRNA targets using miRWalk_miRNA_Targets, selecting only those validated in miRtarBase. Thus, we found 936 mRNA targets of the 15 urinary exosome-miRNAs up-regulated in T2^high^ vs T2^low^ and healthy (Supplementary Table S5). Enrichment analyses of these targets resulted in 123 reactome pathways overrepresented (FDR < 0.05; Supplementary Table S6); the top 20 are depicted in Figure 6B. Many of these pathways are highly related to the immune system, including “HSA-1280215: Cytokine Signaling in Immune System”, “HSA-9006936: Signaling by TGFB family members”, “HSA-983705: Signaling by the B Cell Receptor (BCR)”, or “HSA-5663205: Infectious Disease” (Figure 6B; Supplementary Table S6).

Finally, using MCC topological properties with CytoHubba app (Cytoscape), we have identified the top 20 hub mRNA targets of urinary exosome-miRNAs up-regulated in T2^high^ asthma (Figure 6C). We have also performed pathway enrichment analyses on those targets (Supplementary Figure S2). Interestingly, one of the most enriched reactome pathway was “HSA-6785807: Interleukin-4 and Interleukin-13 Signaling” (Supplementary Figure S2), which is closely related to T2^high^ inflammation.

## 4. Discussion

In recent years, different “omic” technologies have emerged and opened an umbrella of opportunities for disease biomarker discovery, specially using non-invasive and clinically accessible samples such as serum or urine. In this regard, the study of urinary proteome and transcriptome was extensively used in biomarker discovery studies of renal pathologies, but not in respiratory disease. In this work, we have developed a protocol that combines the study of the urinary proteome (exosome-free) with the study of urinary exosome components that can be applied for the molecular biomarker discovery of non-renal disorders. Using this protocol, we could detect many proteins expressed in non-renal tissues (e.g., Liver, N = 324; Brain, N = 202; Placenta, N = 139; or Lung, N = 95), and proteins expressed in the lung were overrepresented (FE = 1.31; FDR = 0.0335). Indeed, we could detect between 10 and 15% of already described biomarkers of respiratory diseases. The proteins detected in our study were classified in different clusters based both on functional categories (Reactome pathways and GO-Biological Processes Categories) and interactome analyses. In general terms, six pathways are highlighted: Immune system, Haemostasis, Extracellular Matrix Organization/Cell adhesion, Signalling, Metabolism, and Cell Death. All of these are important pathways implicated in several respiratory diseases. In parallel, exosomal miRNA was isolated from urine samples, and the expression of several miRNAs previously related to respiratory diseases (i.e., miR-16-5p, miR-21-5p, miR-215-5p, miR-126-3p, and miR-146a-5p) was demonstrated using pre-amplification techniques (i.e., RTqPCR). Finally, 333 miRNAs were detected in urinary exosomes using a non-amplification technique (i.e., nCounter Nanostring); 22 of them were found with changes between T2^high^ asthma, T2^low^ asthma, and healthy subjects. Fifteen of these miRNAs were up-regulated in urinary exosomes from T2^high^ patients, and many of their targets were associated with immune system pathways. Altogether, our study can serve as a basis for the development of studies for discovering biomarkers of several non-renal and particularly respiratory diseases using urine as a non-invasive and clinically accessible sample.

Urine has advantages and disadvantages over other biomarker sources for studying respiratory diseases [33]. Urine is an ideal sample in the respiratory field for studying eicosanoids (Prostaglandins, Thromboxanes, Leukotrienes, and Isoprostanes) using lipidomic approaches [12,33]. Moreover, the use of urine metabolomics in asthma phenotyping was also demonstrated in several studies, especially in children [10,34,35,36]. For example, Mattarucchi et al. described a reduction in urocanic acid release, together with lower methyl-imidazoleacetic acid release in asthmatic children [36]. Different metabolites were also associated with corticosteroid-resistant asthma (e.g., γ-glutamylcysteine, cysteine-glycine, dihydronicotinic acid, 1,2-dihydronaphthalene-1,2-diol, 3,4-dihydro-l-phenylalanine) [35] or used to differentiate uncontrolled asthma from controlled asthma and healthiness (e.g., stearic acid, uric acid, acetylgalactosamine, threitol, aspartic acid, heptadecanoic acid, hypoxanthine, and xanthosine). On the other hand, urinary proteomics is an extensively used approach for the study of renal-/urinary-associated disorders (e.g., diabetic nephropathy, IgA nephropathy, lupus nephritis, membranous nephropathy, segmental glomerulosclerosis, chronic renal insufficiency, etc.) [1,37,38]. Furthermore, different biomarker discovery works were performed using urine in several brain (e.g., Parkinson’s disease, Alzheimer’s disease, Autism, Medulloblastoma, Stroke), and cardiovascular (e.g., Coronary heart disease, hypertension, myocardial infection, diabetes, obesity) disorders, or other non-renal diseases (e.g., rheumatoid arthritis, hepatocellular carcinoma, Rhabdomyolysis, Vitiligo, Irritable Bowel Syndrome, or Kawasaki disease), both in adults and children populations (Supplementary Table S7; [3,4,5,6,7,8,39,40,41,42,43,44,45,46,47,48,49,50,51,52,53,54,55,56]). The urinary proteome of healthy subjects is also known [57]. However, the study of urine proteome associated with respiratory diseases was not extensively addressed.

Although urine is biassed towards systemic changes [33], many of the identified proteins using the exposed protocol are expressed in the lungs (N = 95); indeed, those proteins are overrepresented in our samples compared to the whole human proteome (FE = 1.31; FDR = 0.0335). Different biomarkers of respiratory disease already described in the Disgenet database were also found in our urine proteome samples (Supplementary Table S1). In addition, our group has previously shown changes associated with asthma in the serum levels of many of the proteins detected in urine, i.e., IGFALS, FCN2, HSPG2, CD26/DPP4, and CD14, and suggested their use as potential phenotype/severity biomarkers of this disease [58,59,60,61,62]. To our knowledge, there are no studies in humans using urinary proteomics for asthma or COPD research, but some authors have performed this approach in animal models of disease. In this regard, the group of Qin et al. described some early (e.g., CRAMP, ECOP, HP, F2m AGP1m, and CFB) and late (VDBP, HP, CTSE, PIGR, AAT, TRFE, and HPX) response biomarkers of asthma using an ovalbumin (OVA)-induced mouse model of asthma [63]. Although using healthy individuals, we could detect some of these proteins in our human proteome samples, including HP, F2, CFB, PIGR, or HPX. The same group have recently developed a proteomic biomarker discovery study using urine from a cigarette-smoke (CS)-induced COPD rat model [64]. Using this model, they could detect thirteen proteins with changes in CS-induced COPD rats [64]; we have identified six of them in the exposed human urine proteome: PLAU, PLG, FN1, SCPEP1, PEBP1, F11R. Although not in COPD, Airoldi et al. have identified five proteins with changes in human smokers vs. non-smokers: S100A8, AZGP1, CD59, ITIH4, AMY2A [65]; all of them detected in the present study. Finally, changes in urine proteome were also associated with dyspnoea in USA military personnel after returning from Iraq or Afghanistan [66].

Animal models of other lung diseases were also used for biomarker discovery using urine [67]. In this regard, using an animal model of pulmonary fibrosis (PF), Wu et al. detected 13 proteins in urine from rats treated with Bleomycin (BLM), and also some proteins with changes after prednisone treatment (i.e., CALB1 and FBLN5) [68]. Between them, we have detected COL1A1, UCHL1, CALB1, and FNLN5 in human urine. Regarding lung cancer research, Zhang et al. have identified two proteins, A1BG and LRG1, highly up-regulated in tumour-bearing mice urine, and this result was further confirmed in lung cancer patients [69]. We have identified those two proteins with high confidence in all the samples measured. Another example is the work of Wei and collaborators, in which they have found several proteins with changes at different time points (2, 4, 6, and 9 days) after tail-vein injection of Walker-256 cells in rats (lung cancer metastasis model) [70]. They have validated 20 (17 with human homologues) using parallel reaction monitoring (PRM), and we have detected 14 (82%) of them in our study: LGALS3BP, ORM1, ABHD14B, PIGR, LCN2, GGT1, AMBP, APOE, SERPINA3, EGF, CTSC, GC, ALB, and BTD [70]. Urine was also used for lung cancer biomarker discovery studies in human subjects. For example, Zhang et al. have identified five proteins in urine with relevance for the diagnosis of NSCLC (CLU, KLK1, GSN, LRG1, and SERPINA3) [71], which we detected in this study. In a recent study published by Zhang et al. in 2018, a set of 68 proteins were identified as lung cancer biomarker candidates [72]. We could detect 32.3% of them (N = 22) in our healthy proteome. Of note, several exosomal markers (e.g., PDCD6IP, HSPA8, TSG101, and RAB proteins) have a relatively high abundance in the urine proteome described by Zhang et al. [72], and we are using exosome-free urine in our proteomic protocol.

Another example of the study of biomarkers in pulmonary pathologies using urinary proteomics in humans is obstructive sleep apnoea (OAS). For example, urinary proteomic analyses in children showed different proteins in urine (the most relevant CUBN, COL6A1, OLFM4, ORM1, FABP3, and GC) that facilitated the diagnosis of OAS in this population group [73]. We could detect all of them in adult urine except FABP3. Studying obesity with and without OSA, changes (unadjusted for multiple comparisons) in some peptides belonging to CDH13, FGB, COL1A1, and COL3A1 were also identified [74]. We have detected both CDH13 and COL1A1 in the studied proteome. Urinary proteome studies also have a great potential for respiratory disease biomarker studies using new-born subjects. Thus, Starodubtseva et al. have identified 36 proteins with changes in urine that distinguish new-borns with respiratory pathologies [75]. We detected 29 of them in our study (81%), including AGT, GPX3, SERPINC1, CUBN, SERPINA4, TTR, PIK3IP1, LAMP1, SERPINF1, CD55, IDH1, VASN, THY1, SERPINA1, F2, CSPG4, LRP2, DSG2, REG1A, CDH2, APOD, CDH5, NCAM1, CDH11, HP, UMOD, AGRN, LCN2, and GAPDH.

Finally, respiratory infections are one of the major causes of death and socioeconomic problems worldwide, and they are responsible for exacerbating the chronic respiratory diseases mentioned above. In this regard, “omic” techniques can provide insights into the pathogenesis of these infections. Thus, Bi et al. have demonstrated that urinary proteome is more informative than serum for classifying COVID-19 patients in terms of severity [76]. More examples of this are the studies of Tian et al. [77], in which they demonstrated the presence of immunosuppression in early stages of COVID-19 infection, or the study of Li et al. [78]. Urine was also used for identification of tuberculosis (TB) biomarkers [79]. For example, Young and collaborators have identified seven immune-related proteins discriminators for TB (RBP4, IGKC, ORM1, PTGDS, SECTM1, IGLC2, and AMBP) [80]; all of them are detected in the urine proteome described. Similarly, Liu et al. have provided a panel of biomarkers, including GPX3, NTM, PVR, and HMCN2, as diagnostic biomarkers of TB and also allow us to differentiate active TB from latent TB [81]; four of them were detected in the urine proteome described in this work.

Apart from study of human urinary exosome-free proteome, the protocol we have developed allows us to study urinary exosome content using the same samples. Elliot and collaborators have previously demonstrated the presence of urinary exosomal miRNAs associated with respiratory pathology, particularly IPF [19]. Moreover, they also evidence a disease-modifying ability of those exosomes, triggering a pro-fibrotic phenotype in the lung [19]. As an example of the application of our protocol, we have isolated total RNA content from exosomes and detected several miRNAs (i.e., miR-16-5p, miR-21-5p, miR-126-3p, miR-146a-5p, and miR-215-5p) previously related to respiratory disease [21,82] using RTqPCR. A previous study from our group have demonstrated the association of these miRNAs with asthma severity [21]. Moreover, miR-21-5p and miR-126-3p were increased in T2-high allergic patients compared to T2^high^ non-allergic and T2^low^ patients, highlighting the association between these miRNAs and allergic and T2^high^ inflammation [21]. miR-21 was shown to target IL12p35 and thus favour Th1 differentiation over Th2 inflammation [83]. Hence, this miRNA was found up-regulated in different mouse models of allergic asthma and in serum/plasma from asthma patients [84,85]. In the same manner, miR-126-3p was found increased both in allergic rhinitis and allergic asthma and was related to Th2 inflammation [86]. On the other hand, miR-146a was extensively studied in ageing and related to systemic inflammation [87]. This miRNA has an immunomodulatory role, mainly targeting the NF-kB pathway [87]. Similar to miR-21-5p and miR-126-3p, miR-146a-5p was also found increased in severe asthma [21]. Finally, miR-215-5p is specifically expressed by Th2 cells and previously related to cystic fibrosis patients [88].

We have also used a non-target strategy (Nanostring) to identify potential miRNAs with changes in asthma or different molecular phenotypes of the disease (T2^high^ vs T2^low^). In this study, we confirm the presence of miR-16 and miR-146a, but a pre-amplification of the sample (i.e., RTqPCR) is necessary to detect miR-21-5p, miR-126-3p, and miR-215-5p. We detected 333 miRNAs in urinary exosomes using this nCounter technology, 22 with changes between healthy, T2^high^ and T2^low^ asthma in this proof-of-concept study. Most of the miRNAs with changes were up-regulated in T2^high^ patients, including some of them previously associated with asthma in different samples. For example, the let-7 family, including miR-let-7a, 7b, and 7c, are increased in BAL samples from children with asthma [89]. Plasma levels of miR-Let-7c and miR-1260a (also up-regulated in our T2^high^ patients) were found increased in children with asthma compared to healthy patients [90]. Let-7a was also enriched in serum-free or serum exosomes from adult severe asthma patients vs. healthy donors in a similar way as miR-21 [91]. MiR-let-7a and miR-let-7b function was previously associated with allergic airway inflammation in different experimental models of disease [92,93]. Another miRNA up-regulated in urinary exosome samples from T2^high^ vs healthy in our work, miR-30-5p, was found to be up-regulated in extracellular vesicles from nasal mucus of severe allergic rhinitis compared to healthy patients [94]. miR-23a was also associated with asthma pathogenesis by other authors. Thus, miR-23a was found to be increased in the lung tissue of rats after antigen-induced pulmonary inflammation [95]. Moreover, both TSLP and IL-4, closely associated with T2^high^ inflammation, induced the up-regulation of this miRNA, which resulted in the abrogation of CXCL12 and BCL2 expression in bronchial fibroblasts and smooth muscle cells [95]. Finally, miR-4516 and miR-4286, both up- and down-regulated in urinary exosomes from T2^high^ and T2^low^ asthma patients, respectively, were also previously associated with asthma [96,97]. miR-4286 in whole blood was associated with frequent exacerbations in children with asthma [96]. On the other hand, miR-4516 in serum was found to be up-regulated in adult asthma patients vs. healthy donors [97]. Altogether, our results highlight that urinary exosome miRNAs could be a novel and important source of molecular biomarkers of respiratory pathology.

Our study has several limitations. First, exosomal markers (e.g., PDCD6IP, HSPA8, TSG101, and RAB proteins) have a relatively high abundance in urine samples. We are characterizing exosomal-free urinary proteome. Thus, this could be one of the reasons we detected lower numbers of proteins compared to some of the studies previously mentioned. Furthermore, we used healthy subjects, so we failed to detect some biomarkers that are potentially up-regulated in response to the disease. However, we could detect many of the already published biomarkers for different respiratory diseases, and our urine proteome is enriched in proteins from non-renal tissues, especially the lung, making it a suitable source of respiratory disease biomarkers. We could not detect all miRNAs in all samples studied, and the expression levels of the different miRNAs changed compared to serum. Thus, the study of specific miRNAs in urine needs to be preceded by an expression test in this sample type. In addition, the study of miRNAs in urinary exosomes needs to be performed in autumn, when the miRNAs expression is higher, as shown in our previous study [21].

## 5. Conclusions

In conclusion, we have developed a protocol for the study of non-renal disease biomarkers in urine using proteomic and transcriptomic techniques. Our protocol allows for the study of exosome-free urinary proteome and, at the same time, the study of urinary exosomes (i.e., miRNAs). Thus, this provides different layers of information and increases the potential discovery of molecular biomarkers of disease. This is particularly interesting for heterogeneous respiratory diseases such as asthma or COPD, in which there are no studies in humans.

## Figures and Tables

**Table 1:** Functional annotation clustering analysis using DAVID database for GO-Biological Processes and reactome pathways.

Cluster	Most Relevant Pathway/Biological Process	N	ES	FE	FDR
**Immune system**					
1	*R-HSA-168249: Innate immune system*	122	33.271	3.115	3.42 × 10^−29^
3	*GO:0002250: Adaptive immune system*	47	13.537	3.779	5.97 × 10^−12^
6	*GO:0006958: Complement activation, classical pathway*	31	10.222	9.066	3.46 × 10^−17^
10	*R-HSA-166658: Complement cascade*	17	5.521	7.954	7.88 × 10^−9^
30	*R-HSA-168898: TLR cascades*	8	1.156	1.383	9.21 × 10^−1^
32	*R-HSA-2132295: MHC class II antigen presentation*	8	1.075	1.765	9.08 × 10^−1^
37	*R-HSA-447115: IL-12 family signalling*	6	0.878	2.857	4.08 × 10^−1^
43	*R-HSA-8953897: Cellular responses to stimuli*	21	0.531	0.714	9.78 × 10^−1^
44	*R-HSA-168255: Influenza infection*	4	0.356	0.696	9.30 × 10^−1^
53	*R-HSA-9658195: Leishmania infection*	6	0.010	0.641	9.60 × 10^−1^
**Haemostasis**					
2	*R-HSA-109582: Haemostasis*	70	19.675	3.059	3.70 × 10^−15^
9	*GO:0042730: Fibrinolysis*	10	6.317	19.086	9.80 × 10^−8^
24	*R-HSA-140837: Intrinsic Pathway of Fibrin Clot Formation*	8	2.258	9.439	2.49 × 10^−4^
31	*GO:0072378: Blood coagulation, fibrin clot formation*	4	1.104	20.722	2.16 × 10^−2^
**Extracellular Matrix Organization/Cell adhesion**					
4	*R-HSA-1500931: Cell–cell communication*	26	12.478	5.470	3.69 × 10^−10^
12	*R-HSA-420597: Nectin/Necl trans heterodimerization*	7	3.972	27.137	5.29 × 10^−7^
14	*R-HSA-1474228: Degradation of the extracellular matrix*	22	3.480	4.264	1.15 × 10^−6^
17	*GO:0098742: Cell–Cell adhesion* via *plasma-membrane adhesion molecules*	10	3.030	9.298	7.34 × 10^−5^
40	*R-HSA-199991: Membrane trafficking*	19	0.685	0.813	9.21 × 10^−1^
50	*R-HSA-5619115: Disorders of transmembrane transporters*	5	0.037	0.543	9.84 × 10^−1^
**Signalling**					
5	*R-HSA-381426: Regulation of Insulin-like Growth Factor (IGF) transport and uptake by Insulin-like Growth Factor Binding Proteins (IGFBPs)*	37	10.866	8.033	1.12 × 10^−20^
11	*GO:0007169: Transmembrane receptor PTK signalling pathway*	16	4.315	4.396	2.72 × 10^−4^
26	*R-HSA-3928664: Ephrin signalling*	6	1.963	8.570	7.40 × 10^−3^
31	*R-HSA-5683057: MAPK family signalling cascades*	13	1.104	1.085	9.21 × 10^−1^
38	*R-HSA-6806834: Signalling by MET*	8	0.819	2.748	2.15 × 10^−1^
42	*R-HSA-177929: Signalling by EGFR*	4	0.586	2.171	9.21 × 10^−1^
48	*R-HSA-1257604: PIP3 activates AKT signalling*	5	0.063	0.508	9.90 × 10^−1^
49	*R-HSA-9006931: Signalling by nuclear receptors*	9	0.058	0.817	9.21 × 10^−1^
51	*R-HSA-195721: Signalling by WNT*	4	0.026	0.327	1.00
52	*R-HSA-194315: Signalling by Rho GTPases*	14	0.017	0.537	9.99 × 10^−1^
54	*R-HSA-372790: Signalling by GPCR*	7	0.005	0.268	1.00
**Metabolism**					
7	*R-HSA-5663084: Diseases of carbohydrate metabolism*	11	6.897	8.780	6.73 × 10^−6^
8	*R-HSA-71387: Metabolism of carbohydrates*	46	6.389	4.232	1.68 × 10^−14^
16	*R-HSA-70326: Glucose metabolism*	11	3.382	3.280	2.35 × 10^−2^
18	*R-HSA-2022377: Metabolism of Angiotensinogen to Angiotensins*	7	2.724	10.553	5.62 × 10^−4^
19	*R-HSA-174824: Plasma lipoprotein assembly, remodelling, and clearance*	12	2.604	4.401	1.29 × 10^−3^
20	*GO:0006869: Lipid transport*	10	2.529	4.075	2.38 × 10^−2^
21	*R-HSA-1660662: Glycosphingolipid metabolism*	10	2.513	5.899	6.71 × 10^−4^
22	*GO:0019915: Lipid storage*	4	2.399	5.579	4.00 × 10^−1^
27	*R-HSA-156590: Glutathione conjugation*	8	1.839	6.030	4.52 × 10^−3^
28	*R-HSA-189085: Digestion of dietary carbohydrates*	4	1.551	9.868	6.93 × 10^−2^
29	*R-HSA-159740: Gamma carboxylation of protein precursors*	5	1.278	8.141	3.64 × 10^−1^
35	*R-HSA-3299685: Detoxification of ROS*	7	0.956	5.134	2.65 × 10^−2^
36	*R-HSA-3781860: Diseases associated with N-glycosylation of proteins*	4	0.918	5.427	2.72 × 10^−1^
41	*R-HSA-8953854: Metabolism of RNA*	5	0.594	0.201	1.00
45	*R-HSA-70268: Pyruvate metabolism*	3	0.241	2.626	9.21 × 10^−1^
56	*R-HSA-74160: Gene expression (Transcription)*	12	0.000	0.217	1.00
**Cell death**					
33, 47	*R-HSA-9612973: Autophagy*	6	1.052	1.078	9.21 × 10^−1^
34	*GO:2000352: Negative regulation of endothelial cell apoptotic process*	5	1.025	5.037	2.30 × 10^−1^
46	*R-HSA-109581: Apoptosis*	6	0.207	0.905	9.21 × 10^−1^
**Others**					
13, 23, 25	*R-HSA-1638074: Keratan sulphate/Keratin metabolism*	9	3.877	7.183	4.70 × 10^−4^
15	*R-HSA-1480926: O2/CO2 exchange in erythrocytes*	5	3.435	10.437	1.35 × 10^−2^
39	*R-HSA-1266738: Developmental Biology*	42	0.800	1.01	9.21 × 10^−1^
55	*R-HSA-1640170: Cell cycle*	4	0.000	0.157	1.00

N = number of proteins that belong to the specific pathway; ES = enrichment score for the specific cluster; FE = Fold enrichment of the specific pathway compared to the whole human proteome; FDR = false discovery rate. We identified 56 clusters with a positive enrichment score; all of them summarized into 7 broader categories (shown in bold). The most relevant pathway or biological process for specific clusters within each category is shown in italics.

**Table 2:** Differential expressed miRNAs in urinary exosomes from patients with different molecular phenotypes of asthma.

	*baseMean*	*Log2FC*	*lfcSE*	*Stat*	*p Value*
*Asthma vs. Healthy*					
*hsa-miR-574-5p*	40.5671479	−0.9148423	0.35729808	−2.5604455	0.01045381
*hsa-miR-342-3p*	22.7834699	0.8300255	0.34822973	2.38355728	0.01714622
*hsa-miR-488-3p*	17.6996462	−0.8831223	0.38710618	−2.2813439	0.0225281
*hsa-miR-320e*	94.2924488	1.26604719	0.56756082	2.23068109	0.02570226
*hsa-miR-377-3p*	23.1056833	−0.641396	0.30913106	−2.0748353	0.0380018
*hsa-miR-139-3p*	24.4883407	0.69054879	0.33788118	2.04376221	0.04097704
*hsa-miR-34b-3p*	16.6518662	0.80311135	0.40940465	1.96165662	0.04980248
*T2^high^ vs. Healthy*					
*hsa-miR-4454+hsa-miR-7975*	1871.43143	4.84845886	1.09064723	4.4454877	8.77 × 10^−6^
*hsa-miR-4286*	95.6039239	2.34300078	0.58675153	3.99317369	6.52 × 10^−5^
*hsa-let-7b-5p*	91.2725914	1.89394928	0.53071736	3.56865903	0.00035881
*hsa-miR-200c-3p*	38.446332	1.48357943	0.42808608	3.46561006	0.00052903
*hsa-miR-1260a*	30.3256399	1.6754408	0.51630868	3.24503707	0.00117435
*hsa-let-7a-5p*	60.4218451	2.15717229	0.66622303	3.23791312	0.00120407
*hsa-miR-23a-3p*	63.1060858	1.78852509	0.6104412	2.92988923	0.00339083
*hsa-miR-125a-5p*	20.2466154	1.51553846	0.52845536	2.86786468	0.00413252
*hsa-miR-4516*	67.6961056	2.05065792	0.71652204	2.8619607	0.00421029
*hsa-miR-488-3p*	17.6996462	−1.7776733	0.62174119	−2.8591853	0.00424731
*hsa-miR-204-5p*	67.847085	1.9333541	0.71686145	2.69697041	0.00699735
*hsa-miR-10b-5p*	55.5235107	1.73778902	0.67353223	2.58011265	0.00987681
*hsa-miR-191-5p*	27.4502163	1.3938602	0.56984399	2.44603827	0.01444357
*hsa-let-7c-5p*	21.9687882	1.12353434	0.49590837	2.26560875	0.02347535
*hsa-miR-10a-5p*	44.1971719	1.14162491	0.50780162	2.24817107	0.02456528
*hsa-miR-30d-5p*	74.7454413	1.72851576	0.77089042	2.24223277	0.02494633
*hsa-miR-630*	27.9137898	1.15203733	0.53392982	2.15765685	0.03095452
*hsa-miR-301a-3p*	26.8636724	−1.0874893	0.50811571	−2.1402394	0.03233543
*hsa-miR-342-3p*	22.7834699	1.0648488	0.50082727	2.12617975	0.0334883
*hsa-miR-30e-3p*	24.8830148	1.00361175	0.48709092	2.06041976	0.03935843
*hsa-miR-30a-3p*	21.0812686	0.99494462	0.49195509	2.02242977	0.04313197
*T2^low^ vs. Healthy*					
*hsa-miR-4454+hsa-miR-7975*	1871.43143	−2.5504233	0.93497526	−2.7277976	0.00637587
*hsa-miR-4286*	95.6039239	−1.3041977	0.51370779	−2.538793	0.01112356
*hsa-miR-574-5p*	40.5671479	−1.096622	0.44726633	−2.4518322	0.01421309
*hsa-miR-4516*	67.6961056	−1.3061093	0.61141963	−2.1361913	0.03266382
*T2^high^ vs. T2^low^*					
*hsa-miR-4454+hsa-miR-7975*	1871.43143	7.39888211	1.31874659	5.61054123	2.02 × 10^−8^
*hsa-miR-4286*	95.6039239	3.6471985	0.7116184	5.12521669	2.97 × 10^−7^
*hsa-miR-4516*	67.6961056	3.35676722	0.85783089	3.91308736	9.11 × 10^−5^
*hsa-miR-1260a*	30.3256399	2.3236675	0.60848414	3.8187807	0.00013411
*hsa-let-7a-5p*	60.4218451	2.75193846	0.79141495	3.47723839	0.00050661
*hsa-let-7b-5p*	91.2725914	2.12931239	0.63735641	3.34085034	0.00083522
*hsa-miR-200c-3p*	38.446332	1.62529815	0.50490333	3.21902838	0.00128626
*hsa-miR-191-5p*	27.4502163	1.98309985	0.67706047	2.92898484	0.00340071
*hsa-miR-23a-3p*	63.1060858	1.98414825	0.72983499	2.71862581	0.00655537
*hsa-miR-10b-5p*	55.5235107	2.15897028	0.80699947	2.67530571	0.00746611
*hsa-let-7c-5p*	21.9687882	1.4493829	0.59007958	2.45624986	0.01403955
*hsa-miR-125a-5p*	20.2466154	1.41552655	0.61068218	2.3179431	0.02045241
*hsa-miR-630*	27.9137898	1.35069962	0.64138636	2.10590637	0.03521249
*hsa-miR-10a-5p*	44.1971719	1.26924038	0.6064849	2.09278152	0.03636866
*hsa-miR-204-5p*	67.847085	1.75264746	0.85172349	2.0577658	0.03961262

## Data Availability

The mass spectrometry proteomics data have been deposited to the PRIDE Archive (http://www.ebi.ac.uk/pride/archive/) via the PRIDE partner repository with the data set identifier PXD046113. All data generated or analyzed during this study are included in this published article [and its supplementary information files].

## Supplementary Materials

The following supporting information can be downloaded at: https://www.mdpi.com/article/10.3390/biom15010060/s1, Supplementary Methods; Figure S1: Supplementary uncropped blots; Figure S2: Reactome pathway enrichment analysis of the top 20 hub mRNA targets of urinary exosome-miRNAs up-regulated in T2^high^ asthma; Supplemetary Tables S1–S7. Supplementary Table S1. Lung disease biomarkers (Disgenet database) detected in the urine proteome of study. Supplementary Table S2. Functional annotation clustering analysis using DAVID database for GO-BP and reactome pathways complete. Supplementary Table S3. Demographic, clinical, haematological and biochemical characteristics of Healthy Donors, T2^high^ and T2^low^ asthma phenotypes Supplementary Table S4. Normalized urinary exosome-miRNA expression from Nanostring analyses Supplementary Table S5. Targets for miRNAs up-regulated in T2high vs. T2low urinary exosomes. Supplementary Table S6. Reactome pathways overrepresented within miRNA targets up-regulated in T2high vs T2low patients Supplementary Table S7. Recent urinary proteomic studies (<10 years) in diseases not associated to the renal system (excluding lung diseases).
