# Supplementary material for: Urinary Proteome and Exosome Analysis Protocol for the Discovery of Respiratory Diseases Biomarkers
Source: Biomolecules. 2025 Jan 3;15(1):60. doi: 10.3390/biom15010060 (PMC11762655; doi:10.3390/biom15010060)
Supplement: Supplementary file 1 [file biomolecules-15-00060-s001.zip › Supplementary Figures.pdf]

Urinary Proteome and Exosome Analysis Protocol for the Discovery of Respiratory Diseases Biomarkers

Laura Martelo-Vidal [1,2], Sara Vázquez-Mera [1,2], Pablo Miguéns-Suárez [1,2], Susana Belén Bravo-López [3], Heidi Makrinioti [4], Vicente Domínguez-Arca [5,6], Javier de-Miguel-Díez [7,8,9], Alberto Gómez-Carballa [10,11,12,13], Antonio Salas [10,11,12,13], Francisco Javier González-Barcala [1,2,14,15,†], Francisco Javier Salgado [1,2,*,†] and Juan José Nieto-Fontarigo [1,2]

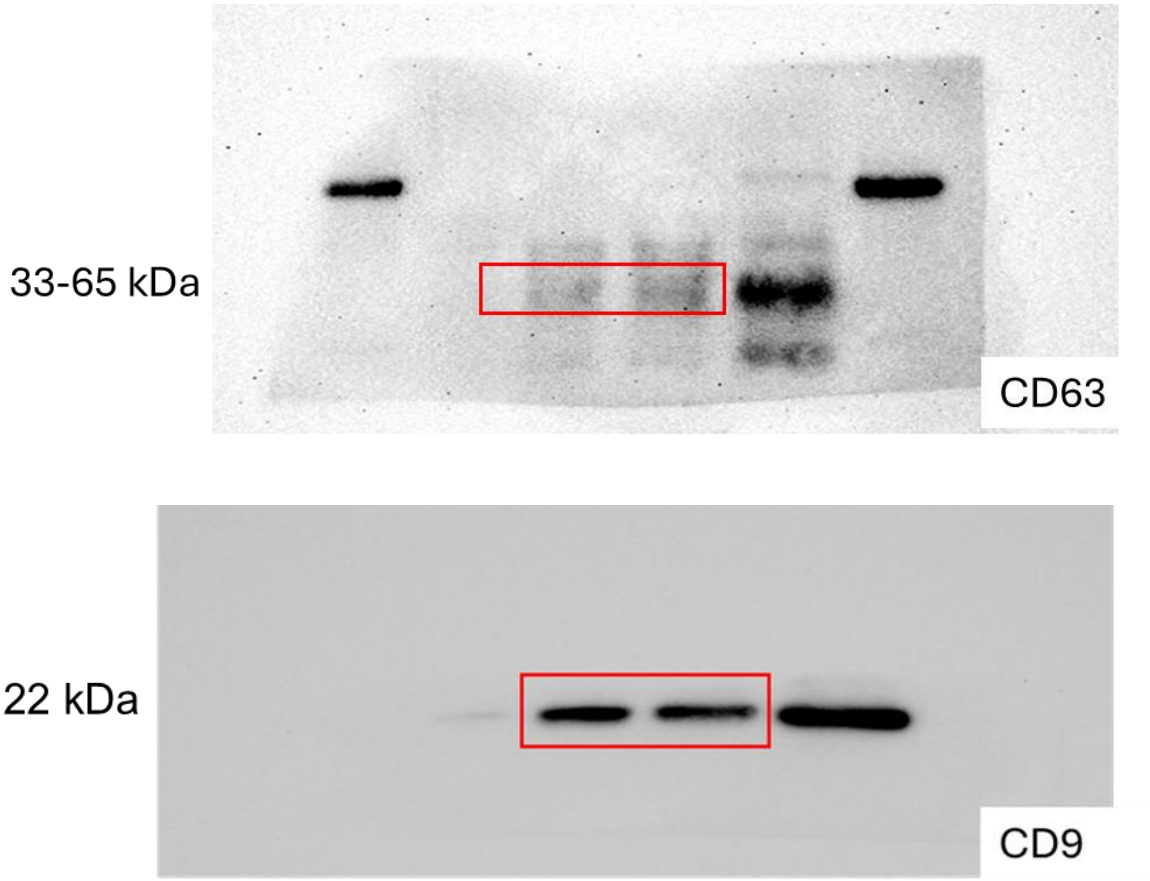

Supplementary Figure S1. Uncropped blots

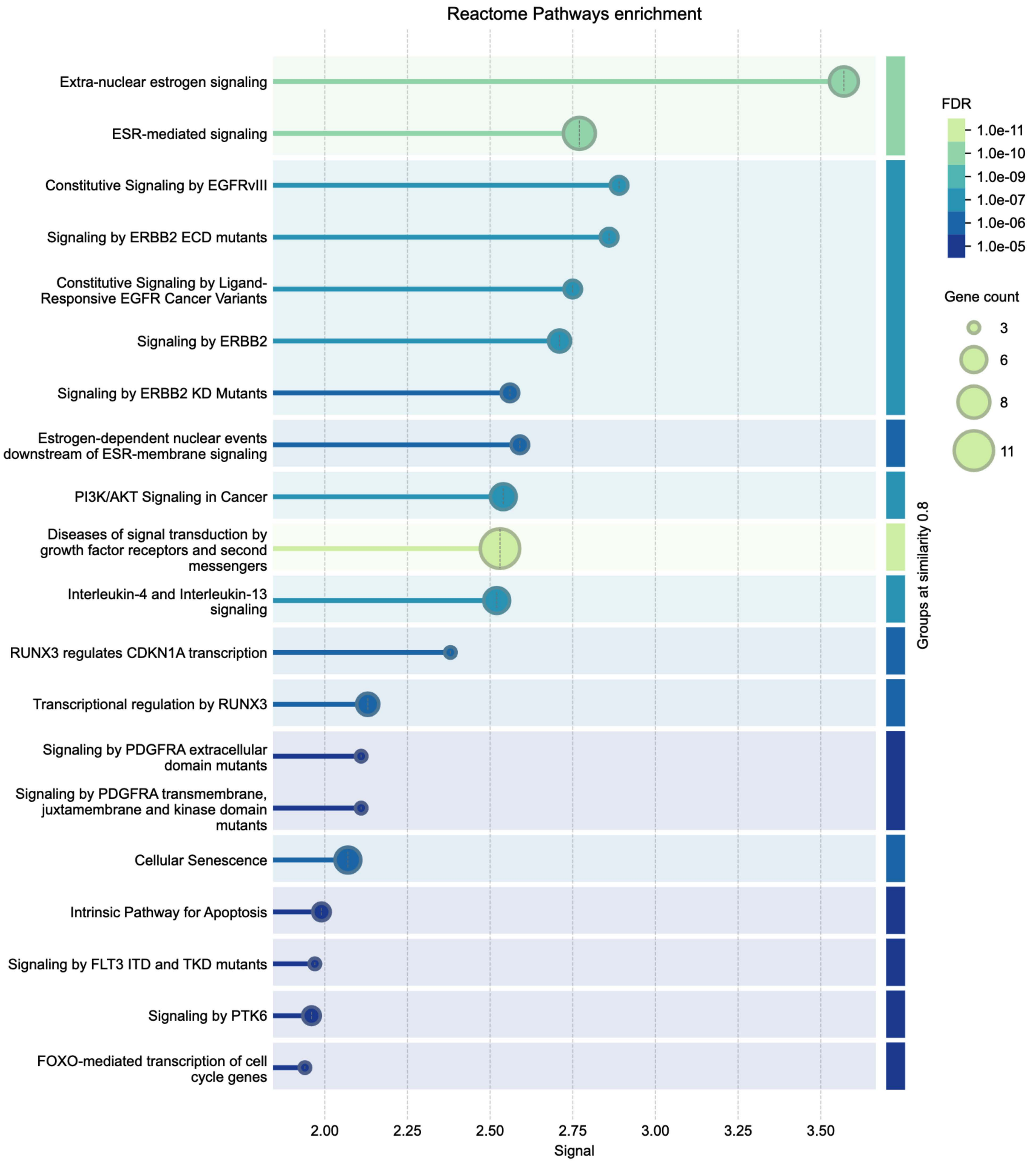

Supplementary Figure S2. Reactome pathway enrichment analysis of the top 20 hub mRNA targets of urinary exo-some-miRNAs up-regulated in T2high asthma
