# Supplementary material for: Urinary Proteome and Exosome Analysis Protocol for the Discovery of Respiratory Diseases Biomarkers
Source: Biomolecules. 2025 Jan 3;15(1):60. doi: 10.3390/biom15010060 (PMC11762655; doi:10.3390/biom15010060)
Supplement: Supplementary file 1 [file biomolecules-15-00060-s001.zip › Supplementary Methods.pdf]

Urinary Proteome and Exosome Analysis Protocol for the Discovery of Respiratory Diseases Biomarkers

Laura Martelo-Vidal [1,2], Sara Vázquez-Mera [1,2], Pablo Miguéns-Suárez [1,2], Susana Belén Bravo-López [3], Heidi Makrinioti [4], Vicente Domínguez-Arca [5,6], Javier de-Miguel-Díez [7,8,9], Alberto Gómez-Carballa [10,11,12,13], Antonio Salas [10,11,12,13], Francisco Javier González-Barcala [1,2,14,15,†], Francisco Javier Salgado [1,2,*,†] and Juan José Nieto-Fontarigo [1,2]

[1] BioLympho Research Group, Department of Biochemistry and Molecular Biology, Faculty of Biology-Biological Research Centre (CIBUS), Universidade de Santiago de Compostela, 15782, Santiago de Compostela, Spain; laura.martelo@rai.usc.es (L.M.-V.); sara.vazquez.mera@rai.usc.es (S.V.-M.); pablo.miguens@rai.usc.es (P.M.-S.); francisco.javier.gonzalez.barcala@sergas.es (F.J.G.-B.); juanjose.nieto.fontarigo@usc.es (J.J.N.-F.)
[2] Translational Research in Airway Diseases Group (TRIAD), Health Research Institute of Santiago de Compostela (IDIS), 15706, Santiago de Compostela, Spain
[3] Proteomic Service, Health Research Institute of Santiago de Compostela (IDIS), 15706, Santiago de Compostela, Spain; susana.belen.bravo.lopez@sergas.es
[4] Department of Emergency Medicine, Massachusetts General Hospital, Harvard Medical School, 02114, Boston, MA, USA; cmakrynioti@mgh.harvard.edu
[5] Grupo de Física de Coloides y Polímeros, Departamento de Física de Partículas, Universidade de Santiago de Compostela, 15782, Santiago de Compostela, Spain; vdominguez@iim.csic.es
[6] Bioprocess Engineering Group, IIM-CSIC, 36208, Vigo, Spain
[7] Respiratory Department, Hospital General Universitario Gregorio Marañón, 28009, Madrid, Spain; javier.miguel@ucm.es
[8] Health Research Institute Gregorio Marañón (IISGM), 28009, Madrid, Spain
[9] Faculty of Medicine, Universidad Complutense de Madrid, 28040, Madrid, Spain
[10] Genetics, Vaccines and Infections Research Group (GenViP), Instituto de Investigación Sanitaria de Santiago, Universidade de Santiago de Compostela, 15706, Santiago de Compostela, Spain; alberto.gomez.carballa@sergas.es (A.G.-C.); antonio.salas@usc.gal (A.S.)
[11] Unidade de Xenética, Instituto de Ciencias Forenses, Facultade de Medicina, Universidade de Santiago de Compostela, 15782, Santiago de Compostela, Spain.
[12] Genética de Poblaciones en Biomedicina (GenPoB) Research Group, Instituto de Investigación Sanitaria (IDIS), Hospital Clínico Universitario de Santiago (SERGAS), 15706, Santiago de Compostela, Galicia, Spain
[13] Centro de Investigación Biomédica en Red de Enfermedades Respiratorias (CIBER-ES), 28029, Madrid, Spain
[14] Department of Respiratory Medicine, University Hospital Complex of Santiago de Compostela, 15706, Santiago de Compostela, Spain
[15] Department of Medicine, Universidade de Santiago de Compostela, 15782, Santiago de Compostela, Spain
* Correspondence: franciscojavier.salgado@usc.es; Tel.: +34-881816936
† These authors have co-directed the work.

Corresponding author

FJ Salgado Castro

Full Professor at University of Santiago de Compostela

Department of Biochemistry and Molecular Biology

Faculty of Biology-Biological Research Centre (CIBUS), Universidade de Santiago de Compostela, Santiago de Compostela, Spain

e-mail: franciscojavier.salgado@usc.es

phone: +34 881816936

Supplementary methods

Urinary proteins quantification

Proteins from exosome-free urine samples were precipitated with methanol-chloroform following a standard protocol (1). Pelleted proteins were resuspended in 100 µL of Rehydration Buffer (7 M Urea, 2 M Thiourea, CHAPS 4% w/v, 500 mM DTT, and 0,5% v/v IPG). Protein concentration was estimated with Bradford Assay according to manufacturer's instructions (Bradford Reagent, Sigma-Aldrich, USA).

Urinary proteome analysis (LC-MS/MS)

For global protein identification and quantification, an equal amount of exosome free urinary proteins (10 µg) was loaded on a 10% SDS-PAGE gel. The run was stopped as soon as the front had penetrated 3 mm into the resolving gel (2, 3). The protein bands were detected by Sypro Ruby fluorescent staining (Lonza, Switzerland), excised, and processed for in-gel, manual tryptic digestion as described elsewhere (4). Gel pieces were reduced with 10 mM dithiothreitol (Sigma-Aldrich, St. Louis, MO) in 50 mM ammonium bicarbonate (Sigma-Aldrich, St. Louis, MO) and alkylated with 55 mM iodoacetamide (Sigma- Aldrich, St. Louis, MO) in 50 mM ammonium bicarbonate. Then, gel pieces were rinsed with 50 mM ammonium bicarbonate in 50% methanol (HPLC grade, Scharlau, Barcelona, Spain), dehydrated by addition of acetonitrile (HPLC grade, Scharlau, Barcelona, Spain), and dried in a SpeedVac. Modified porcine trypsin (Promega, Madison, WI, USA) was added to the dry gel pieces at a final concentration of 20 ng/µl in 20mM ammonium bicarbonate, incubating them at 37 °C for 16 h. After that, peptides were extracted (50% (v/v) ACN/0.1% (v/v) TFA (x3) and ACN (x1)) and separated in a reverse phase liquid column chromatographic (LC) (Nano-LC ultra-System Eksigent).

Eluted peptides were analysed by MS/MS on a TripleTof® 6600 (High Resolution Quadrupole Time of Flight (QTOF instrument)) using a data dependent acquisition (DDA). Protein identification was made using a protein pilot software. The database used was the Human database from UNIPROT indicating variable modifications methionine oxidation and fixed modifications cysteine carbamydometilation (Figure 1B). A complete list of proteins identified in the different samples is provided in as online Supplementary Material.

Exosome characterization

Exosome size distribution was analysed by dynamic light scattering (DLS) using a Zetasizer Nano ZS (Malvern instrument). A 1:100 dilution of exosome suspension in PBS was measured at 25 °C and a period of 10 s was set as equilibration time using a disposable sizing cuvette. Exosome distribution graphs were retrieved from Zetasizer Software (Zetasizer Software v. 7.11, Malvern Analytical).

Exosomal levels of CD9 and CD63 were measured using western blot. Urinary exosomes were lysed using 1X RIPA buffer (25 mM Tris HCl, pH 8.0, 150 mM NaCl, 1% (v/v) NP-40, 1% (w/v) sodium deoxycholate, 0.1% (w/v) SDS), vigorously vortexed, incubated 30 min at 4 °C, sonicated for 30 s and shacked for 15 min in an ice bath. Protein quantification was determined by BCA Protein Assay kit (Thermo Fisher Scientific, Eugene, OR, USA) according to manufacturer´s instructions. 10 and 15 µg of exosomal urinary proteins and exosomal proteins derived from *in vitro*-cultured peripheral blood mononuclear cells (PBMCs as a positive control) were loaded in a 10% SDS-PAGE gel. After electrophoresis, proteins were transferred to a polyvinylidene fluoride membrane and blocked for 1.5 h at RT using a commercial solution (B070-0050, Rockland). After blocking, the membrane

was incubated overnight at 4 °C with primary antibodies (anti-CD9 (1:1,000) (ab263019, Abcam, UK); anti-CD63(1:2,000) (ab134045, Abcam, UK)) diluted in Tris-buffered saline (Tris 20 mM, NaCl 137 mM, pH 7.6) containing 0.1% Tween-20 (Sigma-Aldrich, USA) (TBST), 5% BSA. The membrane was washed five times with TBST followed by incubation with secondary antibody (anti-rabbit-HRP (1:20,000) (4010-05, Southernbiotech, USA)), 1.5 h at RT. It was revealed with ECL chemiluminescence reagent (BosterBio, USA) and visualized in a ChemiDoc Imaging System (Bio-Rad, USA) (Figure 1C).

microRNA (miRNA) isolation from urine exosomes

Two hundred fifty µL of the UC-isolated exosomes were lysed using 750 µL of Trizol-LS (Invitrogen, ThermoFisher Scientific, Massachusetts, USA) and incubated on ice for 30 min. we used Trizol-LS instead of Quiazol like lysis reagent. Then, 200 µL of chloroform was added to the sample and incubated for other 10 min at RT. The mixture was centrifugated (15 min, 12,000 xg, 4 °C) and the upper phase containing RNA (250 to 300 µL) was transferred into a new tube. After adding 1.5 volumes of ethanol, miRNA was isolated using miRNeasy Mini Kit (Qiagen, Germany) modified, and eluted in 50 µL of RNase-free water (Figure 1C).

RT-qPCR

Four µL of exosomal miRNA were reverse transcribed into cDNA using a miRCURY LNA RT Kit (Qiagen, Germany) according to commercial instructions, using SimpliAmp™ Thermal Cycler (Applied Biosystems™, USA). After that, qPCR was performed using the miRCURY SYBR Green PCR kit (Qiagen, Germany), 1:10 cDNA dilution and the following primers: miR-16-5p (YP00205702, Qiagen, Germany), miR-21-5p (YP00204230, Qiagen,

Germany), miR-126-3p (YP00204227, Qiagen, Germany), miR-146a-5p (YP00204688, Qiagen, Germany), miR-215-5p (YP00204598, Qiagen, Germany), miR-103a-5p (YP00204063, Qiagen, Germany). LightCycler®96 thermal cycler (Roche Diagnostics, Basilea, Switzerland) was used with the following cycling conditions: initial activation (2 min; 95 °C), denaturation (10 s; 95 °C) and annealing/extension (60 s; 56 °C) (Figure 1C).

Multiplex miRNA expression analysis.

We used the commercial nCounter® miRNA v3 Expression Panel assay (https://nanostring.com/products/ncounter-assays-panels/immunology/mirna/) to quantify miRNA expression (Figure 1C). This panel allows for the simultaneous quantification of 827 biologically relevant miRNAs. We used 3 µl of total RNA as input and followed standard conditions for both the ligation and hybridization steps.

First, we performed quality control (QC) on the raw data by examining technical parameters to confirm the absence of technical issues, as recommended by the manufacturer. Samples that did not pass technical QC, or had a low number of miRNAs detected, were excluded from the analysis. A background threshold of 20 was established, and only miRNAs with more than 30 counts in at least one sample were included in downstream analysis.

Data normalization was performed using an iterative strategy combining the *DESeq2* (5) and *RUVSeq* (6) packages as described in (7) and using a k value of 1. Ligation control spike-ins, along with the most invariant miRNAs, were used to normalize the samples. The most stable miRNAs for normalization were identified through a naïve differential expression (DE) analysis by comparing (i) asthma vs. healthy controls and (ii) T2high

asthma vs. T2low asthma, and choosing miRNAs meeting these criteria: p-value > 0.1, BaseMean > 100, and |log2FC| < 0.2 in both DE comparisons. This process yielded three invariable miRNAs for downstream normalization: hsa-miR-1183, hsa-miR-1972, and hsa-miR-548ar-5p. Differentially expressed miRNAs were identified using the *DESeq2* (5) package.

1. Wessel D, Flugge UI. A method for the quantitative recovery of protein in dilute solution in the presence of detergents and lipids. Anal Biochem. 1984;138(1):141-3.

2. Bonzon-Kulichenko E, Perez-Hernandez D, Nunez E, Martinez-Acedo P, Navarro P, Trevisan-Herraz M, et al. A robust method for quantitative high-throughput analysis of proteomes by 18O labeling. Mol Cell Proteomics. 2011;10(1):M110 003335.
3. Perez-Hernandez D, Gutierrez-Vazquez C, Jorge I, Lopez-Martin S, Ursa A, Sanchez-Madrid F, et al. The intracellular interactome of tetraspanin-enriched microdomains reveals their function as sorting machineries toward exosomes. J Biol Chem. 2013;288(17):11649-61.
4. Shevchenko A, Wilm M, Vorm O, Mann M. Mass spectrometric sequencing of proteins silver-stained polyacrylamide gels. Anal Chem. 1996;68(5):850-8.
5. Love MI, Huber W, Anders S. Moderated estimation of fold change and dispersion for RNA-seq data with DESeq2. Genome Biol. 2014;15(12):550.
6. Risso D, Ngai J, Speed TP, Dudoit S. Normalization of RNA-seq data using factor analysis of control genes or samples. Nat Biotechnol. 2014;32(9):896-902.
7. Bhattacharya A, Hamilton AM, Furberg H, Pietzak E, Purdue MP, Troester MA, et al. An approach for normalization and quality control for NanoString RNA expression data. Brief Bioinform. 2020.
