# Supplementary material for: Urinary Proteome and Exosome Analysis Protocol for the Discovery of Respiratory Diseases Biomarkers
Source: Biomolecules. 2025 Jan 3;15(1):60. doi: 10.3390/biom15010060 (PMC11762655; doi:10.3390/biom15010060)
Supplement: Supplementary file 1 [file biomolecules-15-00060-s001.zip › Supplementary Tables.pdf]

Urinary Proteome and Exosome Analysis Protocol for the Discovery of Respiratory Diseases Biomarkers

Laura Martelo-Vidal [1,2], Sara Vázquez-Mera [1,2], Pablo Miguéns-Suárez [1,2], Susana Belén Bravo-López [3], Heidi Makrinioti [4], Vicente Domínguez-Arca [5,6], Javier de-Miguel-Díez [7,8,9], Alberto Gómez-Carballa [10,11,12,13], Antonio Salas [10,11,12,13], Francisco Javier González-Barcala [1,2,14,15,†], Francisco Javier Salgado [1,2,*,†] and Juan José Nieto-Fontarigo [1,2]

[1] BioLympho Research Group, Department of Biochemistry and Molecular Biology, Faculty of Biology-Biological Research Centre (CIBUS), Universidade de Santiago de Compostela, 15782, Santiago de Compostela, Spain; laura.martelo@rai.usc.es (L.M.-V.); sara.vazquez.mera@rai.usc.es (S.V.-M.); pablo.miguens@rai.usc.es (P.M.-S.); francisco.javier.gonzalez.barcala@sergas.es (F.J.G.-B.); juanjose.nieto.fontarigo@usc.es (J.J.N.-F.)

[2] Translational Research in Airway Diseases Group (TRIAD), Health Research Institute of Santiago de Compostela (IDIS), 15706, Santiago de Compostela, Spain

[3] Proteomic Service, Health Research Institute of Santiago de Compostela (IDIS), 15706, Santiago de Compostela, Spain; susana.belen.bravo.lopez@sergas.es

[4] Department of Emergency Medicine, Massachusetts General Hospital, Harvard Medical School, 02114, Boston, MA, USA; cmakrynioti@mgh.harvard.edu

[5] Grupo de Física de Coloides y Polímeros, Departamento de Física de Partículas, Universidade de Santiago de Compostela, 15782, Santiago de Compostela, Spain; vdominguez@iim.csic.es

[6] Bioprocess Engineering Group, IIM-CSIC, 36208, Vigo, Spain

[7] Respiratory Department, Hospital General Universitario Gregorio Marañón, 28009, Madrid, Spain; javier.miguel@ucm.es

[8] Health Research Institute Gregorio Marañón (IISGM), 28009, Madrid, Spain

[9] Faculty of Medicine, Universidad Complutense de Madrid, 28040, Madrid, Spain

[10] Genetics, Vaccines and Infections Research Group (GenViP), Instituto de Investigación Sanitaria de Santiago, Universidade de Santiago de Compostela, 15706, Santiago de Compostela, Spain; alberto.gomez.carballa@sergas.es (A.G.-C.); antonio.salas@usc.gal (A.S.)

[11] Unidade de Xenética, Instituto de Ciencias Forenses, Facultade de Medicina, Universidade de Santiago de Compostela, 15782, Santiago de Compostela, Spain.

[12] Genética de Poblaciones en Biomedicina (GenPoB) Research Group, Instituto de Investigación Sanitaria (IDIS), Hospital Clínico Universitario de Santiago (SERGAS), 15706, Santiago de Compostela, Galicia, Spain

[13] Centro de Investigación Biomédica en Red de Enfermedades Respiratorias (CIBER-ES), 28029, Madrid, Spain

[14] Department of Respiratory Medicine, University Hospital Complex of Santiago de Compostela, 15706, Santiago de Compostela, Spain

[15] Department of Medicine, Universidade de Santiago de Compostela, 15782, Santiago de Compostela, Spain

* Correspondence: franciscojavier.salgado@usc.es; Tel.: +34-881816936

† These authors have co-directed the work.

Corresponding author

FJ Salgado Castro
Full Professor at University of Santiago de Compostela
Department of Biochemistry and Molecular Biology
Faculty of Biology-Biological Research Centre (CIBUS), Universidade de Santiago de Compostela, Santiago de Compostela, Spain
e-mail: franciscojavier.salgado@usc.es
phone: +34 881816936

Supplementary Tables

Supplementary Table 1. Lung disease biomarkers (Disgenet database) detected in the urine proteome of study.			
**Disease**	**Biomarkers discovered. (N)**	**Biomarkers detected. N (%)**	**Gene names.**
**Asthma**	302	37 (12.2%)	ACE2;ALB;AMY1A;BSG;C3;CD14;CD33; CST3;DPP4;EGF;F2;FBLN1;FCGR3A;FCGR3B;FN1;GAPDH ;GAS6;GC;GGT1;GOT1; GSTP1;HBA1;HBB;IL13RA1;KNG1;LUM; NCAM1;PECAM1;PLAU;PTGDS;RNASE2; S100A8;SCGB1A1;SERPINA3;SERPING1; TF;TNC
**COPD**	231	30 (13.0%)	ACE2;AGT;ALB;BSG;CD14;CDH1;DPP4; EGF;EPHX2;F2;GC;GGT1;GOT1;GSS; HBA1;HBB;HBG2;HP;ICAM1;MCAM; PECAM1;PIK3IP1;RETN;SCGB1A1; SERPINA1;SERPINA6;SOD3;TTR;TXN; VCAM1
**Lung cancer**	1025	103 (10.1%)	
**NSCLC**	803	82 (10.2%)	AKR1C1;ALB;ALDH1A1;ANPEP;AXL;B2M;CA1;CADM1;C D14;CD276;CD44;CD55; CD9;CDH1;CDH13;CDH2;CDH5;CLU; COL18A1;CP;CSF1;CSF1R;CTSB;CTSD; DDR1;DPP4;EFEMP1;EGF;ENG;ENO1;EZR;F2;FCGR3A;F CGR3B;FLNA;FN1;GAPDH; GAS6;GOT1;GSTP1;HAVCR2;HBA1;HBB; HBG2;HP;HRG;HSPB1;IDH1;IGFBP2;KLK3;KRT5;LAMP1; LGALS3;LGALS3BP;LGALS9; LSAMP;LYVE1;NCAM1;NECTIN4;NEU1; PDCD1LG2;PDGFRB;PECAM1;PKM;PLAU;RBP4;RETN;R OR1;S100A8;S100A9; SCGB1A1;SERPINA1;SLC3A2;SPP1;TF; THBS1;THY1;TNC;TTR;TXN;UCHL1; VCAM1
**SCLC**	230	31 (13.5%)	ALB;BSG;CA1;CD14;CD248;CD44;CD9; CDH1;CDH13;CHL1;CSF1R;DDC;DDR1; FN1;GOT1;GSTP1;HAVCR2;HBA1;HBB; ICOSLG;KLK3;KNG1;KRT5;LGALS3BP; NCAM1;PECAM1;PLAU;PVR;SPP1;THY1; TMPRSS2
**Adenocarcinoma**	438	45 (10.3%)	ACE2;ALDH1A1;ANPEP;APOE;AXL;B2M; BSG;CD44;CD9;CDH1;CDH15;CDH2; CDH3;CDH5;CLU;CTSA;CTSB;DPP4;EGF; ENO1;F11R;FGFR2;FN1;GAS6;GOLM1; GOT1;HBA1;HBB;IDH1;IGFBP2;ITIH4; KLK3;KRT5;MELTF;NCAM1;NEO1; PDCD1LG2;PECAM1;PKM;PLAU;PPIB; REG1A;SERPINA1;SPP1;THBS1
**RIs**	446	55 (12.3%)	
**Tuberculosis**	126	11 (8.7%)	ALB;CD14;CST3;F2;GOT1;GUSB;HBA1; HBB;LAMP1;PDCD1LG2;SERPINC1
**Influenza**	134	12 (8.9%)	ALB;CLMP;F2;FCGR3A;FCGR3B;GGT1; GOT1;HBA1;HBB;HP;KLK1;LAMP1
**COVID-19**	327	49 (15%)	ACE2;AGT;ALB;AMY1A;BSG;C3;C4A; CD14;CDH5;CETP;CST3;CTSB;CTSL; DNASE1;DPP4;EGF;F2;FCGR3A;FCGR3B; GC;GGT1;GOT1;HAVCR2;HBA1;HBB; HBG2;HP;HSPG2;ICAM1;IL6ST;KLK3; KNG1;LCN2;LGALS3;MASP2;NCAM1; PECAM1;PLAU;PLG;PPIB;SCGB1A1;SDC1;SERPING1;SPP 1;TF;TMPRSS2;TNC;TTR; TXN
**ILDs**	47	7 (14.9%)	ALB;GOT1;HBA1;HBB;PPIB;SELENBP1; SPP1

COPD, chronic obstructive pulmonary disease; COVID-19, Coronavirus disease 2019; ILDs, interstitial lung diseases; NSCLC, non-small cell lung carcinoma; RIs, respiratory infections; SCLC, small cell lung carcinoma.

**Supplementary Table 2. Functional annotation clustering analysis using DAVID database for GO-BP and reactome pathways complete.**				
**Category**	**Pathway or GP-Biological Process**	**Count**	**FE**	**FDR**
**Annotation Cluster 1: Enrichment Score: 33.2710**				
REACTOME_PATHWAY	R-HSA-6798695~Neutrophil degranulation	97	5.461	2.15E-42
REACTOME_PATHWAY	R-HSA-168249~Innate Immune System	122	3.115	3.42E-29
REACTOME_PATHWAY	R-HSA-168256~Immune System	161	2.167	3.31E-22
**Annotation Cluster 2: Enrichment Score: 19.6753**				
REACTOME_PATHWAY	R-HSA-114608~Platelet degranulation	39	8.204	4.11E-22
REACTOME_PATHWAY	R-HSA-76005~Response to elevated platelet cytosolic Ca2+	39	7.898	1.51E-21
REACTOME_PATHWAY	R-HSA-109582~Hemostasis	70	3.059	3.70E-15
REACTOME_PATHWAY	R-HSA-76002~Platelet activation. signaling and aggregation	43	4.437	4.10E-14
**Annotation Cluster 3: Enrichment Score: 13.5372**				
GOTERM_BP_DIRECT	GO:0006955~immune response	51	3.759	6.28E-13
GOTERM_BP_DIRECT	GO:0002250~adaptive immune response	47	3.779	5.97E-12
GOTERM_BP_DIRECT	GO:0002377~immunoglobulin production	21	8.101	2.07E-10
**Annotation Cluster 4: Enrichment Score: 12.4779**				
GOTERM_BP_DIRECT	GO:0007156~homophilic cell adhesion via plasma membrane adhesion molecules	32	6.747	3.79E-14
REACTOME_PATHWAY	R-HSA-418990~Adherens junctions interactions	16	13.157	1.00E-11
REACTOME_PATHWAY	R-HSA-446728~Cell junction organization	23	6.859	6.92E-11
REACTOME_PATHWAY	R-HSA-1500931~Cell-Cell communication	26	5.470	3.69E-10
REACTOME_PATHWAY	R-HSA-421270~Cell-cell junction organization	18	7.632	4.50E-09
**Annotation Cluster 5: Enrichment Score: 10.8658**				
REACTOME_PATHWAY	R-HSA-381426~Regulation of Insulin-like Growth Factor (IGF) transport and uptake by Insulin-like Growth Factor Binding Proteins (IGFBPs)	37	8.033	1.12E-20
REACTOME_PATHWAY	R-HSA-8957275~Post-translational protein phosphorylation	31	7.789	1.12E-16
REACTOME_PATHWAY	R-HSA-392499~Metabolism of proteins	91	1.255	0.12523046
REACTOME_PATHWAY	R-HSA-597592~Post-translational protein modification	66	1.246	0.31778086
**Annotation Cluster 6: Enrichment Score: 10.2223**				
GOTERM_BP_DIRECT	GO:0006958~complement activation. classical pathway	31	9.066	3.46E-17
GOTERM_BP_DIRECT	GO:0045087~innate immune response	52	3.159	1.88E-10
GOTERM_BP_DIRECT	GO:0050871~positive regulation of B cell activation	18	6.593	2.26E-07
GOTERM_BP_DIRECT	GO:0006910~phagocytosis. recognition	18	6.527	2.51E-07
GOTERM_BP_DIRECT	GO:0006911~phagocytosis. engulfment	19	5.991	3.17E-07
GOTERM_BP_DIRECT	GO:0050853~B cell receptor signaling pathway	18	4.983	1.27E-05
GOTERM_BP_DIRECT	GO:0042742~defense response to bacterium	24	3.719	1.48E-05
**Annotation Cluster 7: Enrichment Score: 6.897461268668276**				
REACTOME_PATHWAY	R-HSA-2206281~Mucopolysaccharidoses	8	19.736	7.94E-07
REACTOME_PATHWAY	R-HSA-5663084~Diseases of carbohydrate metabolism	11	8.780	6.73E-06
GOTERM_BP_DIRECT	GO:0006027~glycosaminoglycan catabolic process	7	21.154	3.22E-05
**Annotation Cluster 8: Enrichment Score: 6.3894**				
REACTOME_PATHWAY	R-HSA-71387~Metabolism of carbohydrates	46	4.232	1.68E-14

REACTOME_PATHWAY	R-HSA-5668914~Diseases of metabolism	38	4.141	1.87E-11
REACTOME_PATHWAY	R-HSA-1630316~Glycosaminoglycan metabolism	25	5.471	9.57E-10
REACTOME_PATHWAY	R-HSA-975634~Retinoid metabolism and transport	15	9.251	1.29E-08
REACTOME_PATHWAY	R-HSA-6806667~Metabolism of fat-soluble vitamins	15	8.480	4.33E-08
REACTOME_PATHWAY	R-HSA-3560782~Diseases associated with glycosaminoglycan metabolism	14	9.266	5.22E-08
REACTOME_PATHWAY	R-HSA-2024096~HS-GAG degradation	11	13.569	6.91E-08
REACTOME_PATHWAY	R-HSA-9694614~Attachment and Entry	10	15.963	7.71E-08
REACTOME_PATHWAY	R-HSA-1793185~Chondroitin sulfate/dermatan sulfate metabolism	13	7.056	5.27E-06
REACTOME_PATHWAY	R-HSA-4420332~Defective B3GALT6 causes EDSP2 and SEMDJL1	9	12.212	6.88E-06
REACTOME_PATHWAY	R-HSA-3560783~Defective B4GALT7 causes EDS. progeroid type	9	12.212	6.88E-06
REACTOME_PATHWAY	R-HSA-3560801~Defective B3GAT3 causes JDSSDHD	9	12.212	6.88E-06
REACTOME_PATHWAY	R-HSA-1638091~Heparan sulfate/heparin (HS-GAG) metabolism	13	6.414	1.33E-05
REACTOME_PATHWAY	R-HSA-3781865~Diseases of glycosylation	20	3.795	2.69E-05
REACTOME_PATHWAY	R-HSA-196854~Metabolism of vitamins and cofactors	23	3.285	4.08E-05
REACTOME_PATHWAY	R-HSA-1971475~A tetrasaccharide linker sequence is required for GAG synthesis	9	9.394	5.93E-05
REACTOME_PATHWAY	R-HSA-3656237~Defective EXT2 causes exostoses 2	7	13.569	1.12E-04
REACTOME_PATHWAY	R-HSA-3656253~Defective EXT1 causes exostoses 1. TRPS2 and CHDS	7	13.569	1.12E-04
REACTOME_PATHWAY	R-HSA-2187338~Visual phototransduction	15	3.877	5.39E-04
REACTOME_PATHWAY	R-HSA-2022928~HS-GAG biosynthesis	7	6.128	0.01141948
REACTOME_PATHWAY	R-HSA-9694516~SARS-CoV-2 Infection	15	1.428	0.90844452
REACTOME_PATHWAY	R-HSA-9679506~SARS-CoV Infections	15	1.124	0.92134831
REACTOME_PATHWAY	R-HSA-5663205~Infectious disease	31	0.830	0.92134831
REACTOME_PATHWAY	R-HSA-9709957~Sensory Perception	18	0.789	0.92134831
**Annotation Cluster 9: Enrichment Score: 6.3167**				
GOTERM_BP_DIRECT	GO:0042730~fibrinolysis	10	19.086	9.80E-08
GOTERM_BP_DIRECT	GO:0051918~negative regulation of fibrinolysis	8	22.316	2.08E-06
REACTOME_PATHWAY	R-HSA-75205~Dissolution of Fibrin Clot	4	8.350	0.10644489
**Annotation Cluster 10: Enrichment Score: 5.52076**				
REACTOME_PATHWAY	R-HSA-166658~Complement cascade	17	7.954	7.88E-09
REACTOME_PATHWAY	R-HSA-977606~Regulation of Complement cascade	15	8.661	3.31E-08
GOTERM_BP_DIRECT	GO:0006956~complement activation	7	10.154	0.00264344
REACTOME_PATHWAY	R-HSA-166663~Initial triggering of complement	7	8.259	0.00234881
GOTERM_BP_DIRECT	GO:2000427~positive regulation of apoptotic cell clearance	4	24.176	0.01425339
REACTOME_PATHWAY	R-HSA-174577~Activation of C3 and C5	4	13.569	0.02995211
**Annotation Cluster 11: Enrichment Score: 4.3151**				
GOTERM_BP_DIRECT	GO:0033674~positive regulation of kinase activity	13	6.458	6.09E-05
GOTERM_BP_DIRECT	GO:0007169~transmembrane receptor protein tyrosine kinase signaling pathway	16	4.396	2.72E-04

GOTERM_BP_DIRECT	GO:0007275~multicellular organism development	20	3.342	6.44E-04
GOTERM_BP_DIRECT	GO:0018108~peptidyl-tyrosine phosphorylation	15	3.913	0.00176378
GOTERM_BP_DIRECT	GO:0046777~protein autophosphorylation	7	1.402	0.95916073
**Annotation Cluster 12: Enrichment Score: 3.9723**				
REACTOME_PATHWAY	R-HSA-420597~Nectin/Necl trans heterodimerization	7	27.137	5.29E-07
GOTERM_BP_DIRECT	GO:0042271~susceptibility to natural killer cell mediated cytotoxicity	3	36.264	0.05535192
GOTERM_BP_DIRECT	GO:0045954~positive regulation of natural killer cell mediated cytotoxicity	4	5.579	0.38353055
**Annotation Cluster 13: Enrichment Score: 3.8768**				
REACTOME_PATHWAY	R-HSA-2022857~Keratan sulfate degradation	7	14.612	7.00E-05
REACTOME_PATHWAY	R-HSA-1638074~Keratan sulfate/keratin metabolism	9	7.183	4.70E-04
GOTERM_BP_DIRECT	GO:0030203~glycosaminoglycan metabolic process	3	10.879	0.35123326
**Annotation Cluster 14: Enrichment Score: 3.4796**				
REACTOME_PATHWAY	R-HSA-3000178~ECM proteoglycans	17	6.070	4.01E-07
REACTOME_PATHWAY	R-HSA-1474228~Degradation of the extracellular matrix	22	4.264	1.15E-06
REACTOME_PATHWAY	R-HSA-2022090~Assembly of collagen fibrils and other multimeric structures	10	4.449	0.00547875
REACTOME_PATHWAY	R-HSA-1442490~Collagen degradation	10	4.240	0.00754241
REACTOME_PATHWAY	R-HSA-1474290~Collagen formation	11	3.317	0.02190629
REACTOME_PATHWAY	R-HSA-1650814~Collagen biosynthesis and modifying enzymes	9	3.645	0.03645468
REACTOME_PATHWAY	R-HSA-186797~Signaling by PDGF	8	3.743	0.05680603
REACTOME_PATHWAY	R-HSA-8948216~Collagen chain trimerization	7	4.317	0.05680603
REACTOME_PATHWAY	R-HSA-419037~NCAM1 interactions	6	3.877	0.16587363
REACTOME_PATHWAY	R-HSA-375165~NCAM signaling for neurite out-growth	6	2.584	0.52748297
**Annotation Cluster 15: Enrichment Score: 3.4345**				
REACTOME_PATHWAY	R-HSA-2168880~Scavenging of heme from plasma	9	18.787	1.20E-07
GOTERM_BP_DIRECT	GO:0042744~hydrogen peroxide catabolic process	10	12.951	4.27E-06
GOTERM_BP_DIRECT	GO:0098869~cellular oxidant detoxification	14	6.593	1.63E-05
GOTERM_BP_DIRECT	GO:0015670~carbon dioxide transport	6	15.542	0.00153952
REACTOME_PATHWAY	R-HSA-1247673~Erythrocytes take up oxygen and release carbon dioxide	5	15.076	0.00320849
GOTERM_BP_DIRECT	GO:0015671~oxygen transport	5	11.332	0.02447286
REACTOME_PATHWAY	R-HSA-1480926~O2/CO2 exchange in erythrocytes	5	10.437	0.01349327
REACTOME_PATHWAY	R-HSA-1237044~Erythrocytes take up carbon dioxide and release oxygen	5	10.437	0.01349327
GOTERM_BP_DIRECT	GO:0042542~response to hydrogen peroxide	7	4.790	0.07409602
GOTERM_BP_DIRECT	GO:0030185~nitric oxide transport	3	21.758	0.12899995
GOTERM_BP_DIRECT	GO:0010942~positive regulation of cell death	6	4.629	0.14873452
REACTOME_PATHWAY	R-HSA-9707564~Cytoprotection by HMOX1	5	2.120	0.92134831

REACTOME_PATHWAY	R-HSA-9707616~Heme signaling	4	2.310	0.92134831
REACTOME_PATHWAY	R-HSA-983231~Factors involved in megakaryocyte development and platelet production	4	0.646	0.94969129
**Annotation Cluster 16: Enrichment Score: 3.3820**				
REACTOME_PATHWAY	R-HSA-70263~Gluconeogenesis	10	7.982	5.80E-05
GOTERM_BP_DIRECT	GO:0006096~glycolytic process	9	7.590	0.00124075
REACTOME_PATHWAY	R-HSA-70326~Glucose metabolism	11	3.280	0.02351158
GOTERM_BP_DIRECT	GO:0061621~canonical glycolysis	4	9.066	0.14585036
REACTOME_PATHWAY	R-HSA-70171~Glycolysis	8	3.058	0.14203893
**Annotation Cluster 17: Enrichment Score: 3.0297**				
GOTERM_BP_DIRECT	GO:0098742~cell-cell adhesion via plasma-membrane adhesion molecules	10	9.298	7.34E-05
GOTERM_BP_DIRECT	GO:0016339~calcium-dependent cell-cell adhesion via plasma membrane cell adhesion molecules	8	6.747	0.0070855
GOTERM_BP_DIRECT	GO:0007043~cell-cell junction assembly	6	5.579	0.09018165
GOTERM_BP_DIRECT	GO:0034332~adherens junction organization	5	6.715	0.11507192
GOTERM_BP_DIRECT	GO:0000902~cell morphogenesis	5	2.108	0.95916073
**Annotation Cluster 18: Enrichment Score: 2.7236**				
REACTOME_PATHWAY	R-HSA-2022377~Metabolism of Angiotensinogen to Angiotensins	7	10.553	5.62E-04
GOTERM_BP_DIRECT	GO:0002003~angiotensin maturation	4	14.505	0.05512615
GOTERM_BP_DIRECT	GO:0003081~regulation of systemic arterial blood pressure by renin-angiotensin	3	18.132	0.16210379
REACTOME_PATHWAY	R-HSA-2980736~Peptide hormone metabolism	9	2.684	0.16749211
**Annotation Cluster 19: Enrichment Score: 2.6041**				
GOTERM_BP_DIRECT	GO:0010873~positive regulation of cholesterol esterification	6	24.176	1.43E-04
GOTERM_BP_DIRECT	GO:0034375~high-density lipoprotein particle remodeling	7	15.865	2.08E-04
GOTERM_BP_DIRECT	GO:0043691~reverse cholesterol transport	7	14.103	4.21E-04
GOTERM_BP_DIRECT	GO:1905920~positive regulation of CoA-transferase activity	5	22.665	0.00206935
GOTERM_BP_DIRECT	GO:0034372~very-low-density lipoprotein particle remodeling	5	20.146	0.00321503
REACTOME_PATHWAY	R-HSA-174824~Plasma lipoprotein assembly. remodeling. and clearance	12	4.401	0.00129329
GOTERM_BP_DIRECT	GO:0033700~phospholipid efflux	5	15.110	0.00913712
GOTERM_BP_DIRECT	GO:0042157~lipoprotein metabolic process	6	9.890	0.01042464
REACTOME_PATHWAY	R-HSA-8964058~HDL remodeling	5	13.569	0.0049454
REACTOME_PATHWAY	R-HSA-8963898~Plasma lipoprotein assembly	6	8.570	0.00740293
GOTERM_BP_DIRECT	GO:0033344~cholesterol efflux	6	8.059	0.02383701
GOTERM_BP_DIRECT	GO:0006869~lipid transport	10	4.075	0.02383701
REACTOME_PATHWAY	R-HSA-8963899~Plasma lipoprotein remodeling	7	5.756	0.01505384

GOTERM_BP_DIRECT	GO:0008203~cholesterol metabolic process	9	3.980	0.04932051
GOTERM_BP_DIRECT	GO:0046470~phosphatidylcholine metabolic process	5	8.634	0.05673779
GOTERM_BP_DIRECT	GO:0030301~cholesterol transport	5	8.242	0.06571567
GOTERM_BP_DIRECT	GO:0034380~high-density lipoprotein particle assembly	4	12.088	0.08434752
REACTOME_PATHWAY	R-HSA-8963888~Chylomicron assembly	4	10.855	0.05516588
REACTOME_PATHWAY	R-HSA-8963901~Chylomicron remodeling	4	10.855	0.05516588
GOTERM_BP_DIRECT	GO:0042158~lipoprotein biosynthetic process	3	21.758	0.12899995
GOTERM_BP_DIRECT	GO:0030300~regulation of intestinal cholesterol absorption	3	21.758	0.12899995
GOTERM_BP_DIRECT	GO:0042632~cholesterol homeostasis	9	3.138	0.13955113
GOTERM_BP_DIRECT	GO:0034374~low-density lipoprotein particle remodeling	4	9.066	0.14585036
REACTOME_PATHWAY	R-HSA-8964043~Plasma lipoprotein clearance	6	4.401	0.10652331
GOTERM_BP_DIRECT	GO:0034384~high-density lipoprotein particle clearance	3	15.542	0.20489079
GOTERM_BP_DIRECT	GO:0070328~triglyceride homeostasis	4	4.029	0.64058919
GOTERM_BP_DIRECT	GO:0046889~positive regulation of lipid biosynthetic process	3	5.440	0.72699105
GOTERM_BP_DIRECT	GO:0006656~phosphatidylcholine biosynthetic process	3	4.352	0.88712356
GOTERM_BP_DIRECT	GO:0015914~phospholipid transport	3	2.590	0.95916073
REACTOME_PATHWAY	R-HSA-382551~Transport of small molecules	26	0.968	0.92134831
REACTOME_PATHWAY	R-HSA-1989781~PPARA activates gene expression	3	0.696	0.93267727
REACTOME_PATHWAY	R-HSA-400206~Regulation of lipid metabolism by PPARalpha	3	0.684	0.93670418
**Annotation Cluster 20: Enrichment Score: 2.5294**				
GOTERM_BP_DIRECT	GO:0034375~high-density lipoprotein particle remodeling	7	15.865	2.08E-04
GOTERM_BP_DIRECT	GO:0006869~lipid transport	10	4.075	0.02383701
GOTERM_BP_DIRECT	GO:0006641~triglyceride metabolic process	6	6.044	0.06689853
REACTOME_PATHWAY	R-HSA-9029569~NR1H3 & NR1H2 regulate gene expression linked to cholesterol transport and efflux	4	2.934	0.86261178
REACTOME_PATHWAY	R-HSA-9024446~NR1H2 and NR1H3-mediated signaling	4	2.310	0.92134831
**Annotation Cluster 21: Enrichment Score: 2.5126240349384594**				
REACTOME_PATHWAY	R-HSA-1660662~Glycosphingolipid metabolism	10	5.899	6.71E-04
GOTERM_BP_DIRECT	GO:0006689~ganglioside catabolic process	4	20.722	0.0216499
REACTOME_PATHWAY	R-HSA-428157~Sphingolipid metabolism	10	3.015	0.06238882
REACTOME_PATHWAY	R-HSA-556833~Metabolism of lipids	27	0.986	0.92134831
**Annotation Cluster 22: Enrichment Score: 2.3987**				
GOTERM_BP_DIRECT	GO:0051651~maintenance of location in cell	4	36.264	0.00399966

GOTERM_BP_DIRECT	GO:0006689~ganglioside catabolic process	4	20.722	0.0216499
GOTERM_BP_DIRECT	GO:0019915~lipid storage	4	5.579	0.40001038
GOTERM_BP_DIRECT	GO:0050885~neuromuscular process controlling balance	4	3.086	0.85282585
**Annotation Cluster 23: Enrichment Score: 2.3956**				
REACTOME_PATHWAY	R-HSA-1638074~Keratan sulfate/keratin metabolism	9	7.183	4.70E-04
REACTOME_PATHWAY	R-HSA-3656244~Defective B4GALT1 causes B4GALT1-CDG (CDG-2d)	3	10.176	0.25244728
REACTOME_PATHWAY	R-HSA-2022854~Keratan sulfate biosynthesis	4	3.877	0.52748297
**Annotation Cluster 24: Enrichment Score: 2.2580**				
REACTOME_PATHWAY	R-HSA-140837~Intrinsic Pathway of Fibrin Clot Formation	8	9.439	2.49E-04
REACTOME_PATHWAY	R-HSA-9651496~Defects of contact activation system (CAS) and kallikrein/kinin system (KKS)	3	5.088	0.67932923
REACTOME_PATHWAY	R-HSA-9671793~Diseases of hemostasis	3	5.088	0.67932923
**Annotation Cluster 25: Enrichment Score: 1.9774**				
GOTERM_BP_DIRECT	GO:0045109~intermediate filament organization	8	4.396	0.05535192
REACTOME_PATHWAY	R-HSA-6809371~Formation of the cornified envelope	13	2.714	0.03614695
GOTERM_BP_DIRECT	GO:0031424~keratinization	7	3.297	0.25514478
REACTOME_PATHWAY	R-HSA-6805567~Keratinization	13	1.649	0.59757022
**Annotation Cluster 26: Enrichment Score: 1.9631**				
REACTOME_PATHWAY	R-HSA-3928664~Ephrin signaling	6	8.570	0.00740293
REACTOME_PATHWAY	R-HSA-3928662~EPHB-mediated forward signaling	7	4.523	0.04700565
REACTOME_PATHWAY	R-HSA-3928665~EPH-ephrin mediated repulsion of cells	7	3.725	0.10620092
GOTERM_BP_DIRECT	GO:0048013~ephrin receptor signaling pathway	6	4.266	0.19027877
REACTOME_PATHWAY	R-HSA-2682334~EPH-Ephrin signaling	9	2.655	0.17457936
GOTERM_BP_DIRECT	GO:0021952~central nervous system projection neuron axonogenesis	3	9.890	0.39049178
GOTERM_BP_DIRECT	GO:0031290~retinal ganglion cell axon guidance	3	5.726	0.72699105
**Annotation Cluster 27: Enrichment Score: 1.8385**				
REACTOME_PATHWAY	R-HSA-156590~Glutathione conjugation	8	6.030	0.00452422
REACTOME_PATHWAY	R-HSA-174403~Glutathione synthesis and recycling	4	9.046	0.08588025
REACTOME_PATHWAY	R-HSA-211859~Biological oxidations	16	1.956	0.15675358
REACTOME_PATHWAY	R-HSA-156580~Phase II - Conjugation of compounds	10	2.490	0.16827147
REACTOME_PATHWAY	R-HSA-9753281~Paracetamol ADME	5	4.679	0.18135075
REACTOME_PATHWAY	R-HSA-5423646~Aflatoxin activation and detoxification	4	5.713	0.24672806
GOTERM_BP_DIRECT	GO:0006750~glutathione biosynthetic process	3	7.771	0.51481289
REACTOME_PATHWAY	R-HSA-9748784~Drug ADME	8	2.193	0.47981971

**Annotation Cluster 28: Enrichment Score: 1.5513**				
REACTOME_PATHWAY	R-HSA-189085~Digestion of dietary carbohydrate	4	9.868	0.06928416
REACTOME_PATHWAY	R-HSA-8935690~Digestion	4	4.934	0.33711269
REACTOME_PATHWAY	R-HSA-8963743~Digestion and absorption	4	4.020	0.49325659
**Annotation Cluster 29: Enrichment Score: 1.2779**				
REACTOME_PATHWAY	R-HSA-159763~Transport of gamma-carboxylated protein precursors from the endoplasmic reticulum to the Golgi apparatus	3	9.046	0.31189491
REACTOME_PATHWAY	R-HSA-159740~Gamma-carboxylation of protein precursors	3	8.141	0.3636533
REACTOME_PATHWAY	R-HSA-159782~Removal of aminoterminal propeptides from gamma-carboxylated proteins	3	8.141	0.3636533
REACTOME_PATHWAY	R-HSA-159854~Gamma-carboxylation. transport. and amino-terminal cleavage of proteins	3	7.401	0.41543207
REACTOME_PATHWAY	R-HSA-163841~Gamma carboxylation. hypusine formation and arylsulfatase activation	5	3.231	0.46634254
**Annotation Cluster 30**: **Enrichment Score: 1.1558**				
REACTOME_PATHWAY	R-HSA-5602498~MyD88 deficiency (TLR2/4)	5	7.982	0.03597097
REACTOME_PATHWAY	R-HSA-5603041~IRAK4 deficiency (TLR2/4)	5	7.538	0.04329923
REACTOME_PATHWAY	R-HSA-5686938~Regulation of TLR by endogenous ligand	5	6.461	0.06928416
REACTOME_PATHWAY	R-HSA-1236975~Antigen processing-Cross presentation	10	2.584	0.14203893
REACTOME_PATHWAY	R-HSA-5260271~Diseases of Immune System	5	4.377	0.2153673
REACTOME_PATHWAY	R-HSA-5602358~Diseases associated with the TLR signaling cascade	5	4.377	0.2153673
REACTOME_PATHWAY	R-HSA-1236974~ER-Phagosome pathway	8	2.412	0.35102482
REACTOME_PATHWAY	R-HSA-166058~MyD88:MAL(TIRAP) cascade initiated on plasma membrane	6	1.612	0.92134831
REACTOME_PATHWAY	R-HSA-168188~Toll Like Receptor TLR6:TLR2 Cascade	6	1.612	0.92134831
REACTOME_PATHWAY	R-HSA-168179~Toll Like Receptor TLR1:TLR2 Cascade	6	1.566	0.92134831
REACTOME_PATHWAY	R-HSA-181438~Toll Like Receptor 2 (TLR2) Cascade	6	1.566	0.92134831
REACTOME_PATHWAY	R-HSA-168898~Toll-like Receptor Cascades	8	1.383	0.92134831
REACTOME_PATHWAY	R-HSA-166016~Toll Like Receptor 4 (TLR4) Cascade	6	1.206	0.92134831
REACTOME_PATHWAY	R-HSA-983169~Class I MHC mediated antigen processing & presentation	11	0.783	0.92134831
**Annotation Cluster 31**: **Enrichment Score: 1.1040**				
GOTERM_BP_DIRECT	GO:0070527~platelet aggregation	8	6.447	0.00856107
GOTERM_BP_DIRECT	GO:0072378~blood coagulation. fibrin clot formation	4	20.722	0.0216499
GOTERM_BP_DIRECT	GO:0051592~response to calcium ion	6	3.509	0.34838755
REACTOME_PATHWAY	R-HSA-6802948~Signaling by high-kinase activity BRAF mutants	5	3.769	0.31747592
REACTOME_PATHWAY	R-HSA-76009~Platelet Aggregation (Plug Formation)	5	3.479	0.38930527

REACTOME_PATHWAY	R-HSA-5674135~MAP2K and MAPK activation	5	3.392	0.40853152
REACTOME_PATHWAY	R-HSA-354192~Integrin signaling	4	4.020	0.49325659
REACTOME_PATHWAY	R-HSA-9649948~Signaling downstream of RAS mutants	5	2.887	0.58043944
REACTOME_PATHWAY	R-HSA-6802946~Signaling by moderate kinase activity BRAF mutants	5	2.887	0.58043944
REACTOME_PATHWAY	R-HSA-6802949~Signaling by RAS mutants	5	2.887	0.58043944
REACTOME_PATHWAY	R-HSA-6802955~Paradoxical activation of RAF signaling by kinase inactive BRAF	5	2.887	0.58043944
REACTOME_PATHWAY	R-HSA-372708~p130Cas linkage to MAPK signaling for integrins	3	5.427	0.62743398
REACTOME_PATHWAY	R-HSA-354194~GRB2:SOS provides linkage to MAPK signaling for Integrins	3	5.427	0.62743398
REACTOME_PATHWAY	R-HSA-9656223~Signaling by RAF1 mutants	4	2.524	0.92134831
REACTOME_PATHWAY	R-HSA-6802952~Signaling by BRAF and RAF1 fusions	5	2.025	0.92134831
REACTOME_PATHWAY	R-HSA-6802957~Oncogenic MAPK signaling	5	1.615	0.92134831
REACTOME_PATHWAY	R-HSA-5684996~MAPK1/MAPK3 signaling	12	1.139	0.92134831
REACTOME_PATHWAY	R-HSA-5683057~MAPK family signaling cascades	13	1.085	0.92134831
REACTOME_PATHWAY	R-HSA-5673001~RAF/MAP kinase cascade	11	1.066	0.92134831
REACTOME_PATHWAY	R-HSA-5663202~Diseases of signal transduction by growth factor receptors and second messengers	10	0.627	0.9806317
**Annotation Cluster 32: Enrichment Score: 1.0753**				
GOTERM_BP_DIRECT	GO:0051603~proteolysis involved in cellular protein catabolic process	5	3.700	0.45975795
GOTERM_BP_DIRECT	GO:0097067~cellular response to thyroid hormone stimulus	3	6.399	0.64058919
REACTOME_PATHWAY	R-HSA-2132295~MHC class II antigen presentation	8	1.765	0.90844452
**Annotation Cluster 33: Enrichment Score: 1.0524**				
REACTOME_PATHWAY	R-HSA-9613829~Chaperone Mediated Autophagy	5	6.168	0.07969575
REACTOME_PATHWAY	R-HSA-9615710~Late endosomal microautophagy	4	3.101	0.78194397
REACTOME_PATHWAY	R-HSA-9612973~Autophagy	6	1.078	0.92134831
**Annotation Cluster 34: Enrichment Score: 1.0250**				
GOTERM_BP_DIRECT	GO:2000352~negative regulation of endothelial cell apoptotic process	5	5.037	0.23048197
GOTERM_BP_DIRECT	GO:1902042~negative regulation of extrinsic apoptotic signaling pathway via death domain receptors	3	3.885	0.95116698
GOTERM_BP_DIRECT	GO:0045907~positive regulation of vasoconstriction	3	2.863	0.95916073
**Annotation Cluster 35**: **Enrichment Score: 0.9560**				
REACTOME_PATHWAY	R-HSA-3299685~Detoxification of Reactive Oxygen Species	7	5.134	0.02648676
GOTERM_BP_DIRECT	GO:0045454~cell redox homeostasis	4	3.373	0.77191989
REACTOME_PATHWAY	R-HSA-5628897~TP53 Regulates Metabolic Genes	4	1.248	0.92134831
REACTOME_PATHWAY	R-HSA-3700989~Transcriptional Regulation by TP53	5	0.375	0.99938814

**Annotation Cluster 36**: **Enrichment Score: 0.9184**				
REACTOME_PATHWAY	R-HSA-4341670~Defective NEU1 causes sialidosis	3	27.137	0.04621361
REACTOME_PATHWAY	R-HSA-3781860~Diseases associated with N-glycosylation of proteins	4	5.427	0.27152706
REACTOME_PATHWAY	R-HSA-4085001~Sialic acid metabolism	3	2.467	0.92134831
REACTOME_PATHWAY	R-HSA-446219~Synthesis of substrates in N-glycan biosythesis	3	1.292	0.92134831
REACTOME_PATHWAY	R-HSA-446193~Biosynthesis of the N-glycan precursor (dolichol lipid-linked oligosaccharide. LLO) and transfer to a nascent protein	3	1.044	0.92134831
**Annotation Cluster 37**: **Enrichment Score: 0.8775**				
REACTOME_PATHWAY	R-HSA-447115~Interleukin-12 family signaling	6	2.857	0.40798969
REACTOME_PATHWAY	R-HSA-8950505~Gene and protein expression by JAK-STAT signaling after Interleukin-12 stimulation	4	2.857	0.90432831
REACTOME_PATHWAY	R-HSA-9020591~Interleukin-12 signaling	4	2.310	0.92134831
**Annotation Cluster 38**: **Enrichment Score: 0.8187**				
REACTOME_PATHWAY	R-HSA-6806834~Signaling by MET	8	2.748	0.2153673
REACTOME_PATHWAY	R-HSA-8874081~MET activates PTK2 signaling	3	2.714	0.92134831
REACTOME_PATHWAY	R-HSA-8875878~MET promotes cell motility	3	1.986	0.92134831
**Annotation Cluster 39**: **Enrichment Score: 0.7999**				
REACTOME_PATHWAY	R-HSA-373760~L1CAM interactions	10	2.280	0.24688442
REACTOME_PATHWAY	R-HSA-422475~Axon guidance	26	1.278	0.90844452
REACTOME_PATHWAY	R-HSA-9675108~Nervous system development	26	1.223	0.92134831
REACTOME_PATHWAY	R-HSA-1266738~Developmental Biology	42	1.011	0.92134831
**Annotation Cluster 40**: **Enrichment Score: 0.6849**				
REACTOME_PATHWAY	R-HSA-5694530~Cargo concentration in the ER	5	4.112	0.24975329
REACTOME_PATHWAY	R-HSA-446203~Asparagine N-linked glycosylation	17	1.518	0.58043944
REACTOME_PATHWAY	R-HSA-204005~COPII-mediated vesicle transport	5	1.995	0.92134831
REACTOME_PATHWAY	R-HSA-948021~Transport to the Golgi and subsequent modification	10	1.467	0.92134831
REACTOME_PATHWAY	R-HSA-199977~ER to Golgi Anterograde Transport	7	1.234	0.92134831
REACTOME_PATHWAY	R-HSA-199991~Membrane Trafficking	19	0.813	0.92134831
**Annotation Cluster 41**: **Enrichment Score: 0.5943**				
GOTERM_BP_DIRECT	GO:0042026~protein refolding	4	5.802	0.35242574
REACTOME_PATHWAY	R-HSA-450408~AUF1 (hnRNP D0) binds and destabilizes mRNA	4	1.938	0.92134831
REACTOME_PATHWAY	R-HSA-450531~Regulation of mRNA stability by proteins that bind AU-rich elements	5	1.542	0.92134831
REACTOME_PATHWAY	R-HSA-8953854~Metabolism of RNA	5	0.201	0.99999998
**Annotation Cluster 42**: **Enrichment Score: 0.5862**				

REACTOME_PATHWAY	R-HSA-5637815~Signaling by Ligand-Responsive EGFR Variants in Cancer	3	4.285	0.86261178
REACTOME_PATHWAY	R-HSA-1236382~Constitutive Signaling by Ligand-Responsive EGFR Cancer Variants	3	4.285	0.86261178
REACTOME_PATHWAY	R-HSA-1643713~Signaling by EGFR in Cancer	3	3.256	0.92134831
REACTOME_PATHWAY	R-HSA-177929~Signaling by EGFR	4	2.171	0.92134831
REACTOME_PATHWAY	R-HSA-1236394~Signaling by ERBB4	4	1.872	0.92134831
REACTOME_PATHWAY	R-HSA-1227986~Signaling by ERBB2	3	1.628	0.92134831
**Annotation Cluster 43**: **Enrichment Score: 0.5311**				
REACTOME_PATHWAY	R-HSA-9711123~Cellular response to chemical stress	14	1.958	0.21827556
REACTOME_PATHWAY	R-HSA-2262752~Cellular responses to stress	21	0.727	0.97196318
REACTOME_PATHWAY	R-HSA-8953897~Cellular responses to stimuli	21	0.714	0.97755343
**Annotation Cluster 44**: **Enrichment Score: 0.35635640927216744**				
REACTOME_PATHWAY	R-HSA-901042~Calnexin/calreticulin cycle	3	3.131	0.92134831
REACTOME_PATHWAY	R-HSA-532668~N-glycan trimming in the ER and Calnexin/Calreticulin cycle	3	2.326	0.92134831
REACTOME_PATHWAY	R-HSA-168255~Influenza Infection	4	0.696	0.930114
**Annotation Cluster 45: Enrichment Score: 0.24057789151344597**				
REACTOME_PATHWAY	R-HSA-70268~Pyruvate metabolism	3	2.626	0.92134831
REACTOME_PATHWAY	R-HSA-71406~Pyruvate metabolism and Citric Acid (TCA) cycle	3	1.480	0.92134831
REACTOME_PATHWAY	R-HSA-1428517~The citric acid (TCA) cycle and respiratory electron transport	3	0.457	0.9904768
**Annotation Cluster 46**: **Enrichment Score: 0.20730027029300585**				
REACTOME_PATHWAY	R-HSA-111465~Apoptotic cleavage of cellular proteins	3	2.142	0.92134831
REACTOME_PATHWAY	R-HSA-75153~Apoptotic execution phase	3	1.566	0.92134831
REACTOME_PATHWAY	R-HSA-5357801~Programmed Cell Death	7	0.905	0.92134831
REACTOME_PATHWAY	R-HSA-109581~Apoptosis	6	0.905	0.92134831
**Annotation Cluster 47: Enrichment Score: 0.0979**				
REACTOME_PATHWAY	R-HSA-9612973~Autophagy	6	1.078	0.92134831
REACTOME_PATHWAY	R-HSA-9663891~Selective autophagy	3	1.005	0.92134831
REACTOME_PATHWAY	R-HSA-1632852~Macroautophagy	3	0.599	0.96281876
**Annotation Cluster 48: Enrichment Score: 0.06346**				
REACTOME_PATHWAY	R-HSA-6811558~PI5P. PP2A and IER3 Regulate PI3K/AKT Signaling	4	1.024	0.92134831
REACTOME_PATHWAY	R-HSA-2219530~Constitutive Signaling by Aberrant PI3K in Cancer	3	1.044	0.92134831
REACTOME_PATHWAY	R-HSA-199418~Negative regulation of the PI3K/AKT network	4	0.961	0.92134831
REACTOME_PATHWAY	R-HSA-2219528~PI3K/AKT Signaling in Cancer	3	0.783	0.92134831
REACTOME_PATHWAY	R-HSA-1257604~PIP3 activates AKT signaling	5	0.508	0.98988988

REACTOME_PATHWAY	R-HSA-9006925~Intracellular signaling by second messengers	5	0.439	0.99697793
**Annotation Cluster 49**: **Enrichment Score: 0.0583**				
REACTOME_PATHWAY	R-HSA-9009391~Extra-nuclear estrogen signaling	3	1.057	0.92134831
REACTOME_PATHWAY	R-HSA-9006931~Signaling by Nuclear Receptors	9	0.817	0.92134831
REACTOME_PATHWAY	R-HSA-8939211~ESR-mediated signaling	4	0.487	0.98979318
**Annotation Cluster 50**: **Enrichment Score: 0.0373**				
REACTOME_PATHWAY	R-HSA-5619102~SLC transporter disorders	3	0.831	0.92134831
REACTOME_PATHWAY	R-HSA-5619115~Disorders of transmembrane transporters	5	0.771	0.92134831
REACTOME_PATHWAY	R-HSA-425407~SLC-mediated transmembrane transport	5	0.543	0.98380629
**Annotation Cluster 51**: **Enrichment Score: 0.0263**				
REACTOME_PATHWAY	R-HSA-4086400~PCP/CE pathway	3	0.885	0.92134831
REACTOME_PATHWAY	R-HSA-3858494~Beta-catenin independent WNT signaling	3	0.558	0.97296659
REACTOME_PATHWAY	R-HSA-195721~Signaling by WNT	4	0.327	0.99967557
**Annotation Cluster 52: Enrichment Score: 0.0172**				
REACTOME_PATHWAY	R-HSA-195258~RHO GTPase Effectors	10	0.830	0.92134831
REACTOME_PATHWAY	R-HSA-194315~Signaling by Rho GTPases	14	0.537	0.99882064
REACTOME_PATHWAY	R-HSA-9716542~Signaling by Rho GTPases. Miro GTPases and RHOBTB3	14	0.525	0.99918306
REACTOME_PATHWAY	R-HSA-9012999~RHO GTPase cycle	6	0.363	0.99981727
**Annotation Cluster 53**: **Enrichment Score: 0.0096**				
REACTOME_PATHWAY	R-HSA-9658195~Leishmania infection	6	0.641	0.95978585
REACTOME_PATHWAY	R-HSA-9662851~Anti-inflammatory response favouring Leishmania parasite infection	3	0.482	0.98718772
REACTOME_PATHWAY	R-HSA-9664433~Leishmania parasite growth and survival	3	0.482	0.98718772
**Annotation Cluster 54**: **Enrichment Score: 0.0048**				
GOTERM_BP_DIRECT	GO:0007204~positive regulation of cytosolic calcium ion concentration	3	0.684	0.95916073
REACTOME_PATHWAY	R-HSA-375276~Peptide ligand-binding receptors	4	0.540	0.98038126
REACTOME_PATHWAY	R-HSA-416476~G alpha (q) signalling events	3	0.377	0.99734024
REACTOME_PATHWAY	R-HSA-373076~Class A/1 (Rhodopsin-like receptors)	4	0.324	0.99970608
REACTOME_PATHWAY	R-HSA-418594~G alpha (i) signalling events	3	0.256	0.99992409
REACTOME_PATHWAY	R-HSA-500792~GPCR ligand binding	5	0.291	0.99997844
REACTOME_PATHWAY	R-HSA-388396~GPCR downstream signalling	6	0.257	0.99999949
REACTOME_PATHWAY	R-HSA-372790~Signaling by GPCR	7	0.268	0.99999976
**Annotation Cluster 55**: **Enrichment Score: 2.2955E-4**				
REACTOME_PATHWAY	R-HSA-68882~Mitotic Anaphase	3	0.345	0.99865776

REACTOME_PATHWAY	R-HSA-2555396~Mitotic Metaphase and Anaphase	3	0.344	0.99870314
REACTOME_PATHWAY	R-HSA-68886~M Phase	3	0.195	0.99999795
REACTOME_PATHWAY	R-HSA-69278~Cell Cycle. Mitotic	3	0.145	0.99999999
REACTOME_PATHWAY	R-HSA-1640170~Cell Cycle	4	0.157	1
**Annotation Cluster 56: Enrichment Score: 6.6452E-5**				
REACTOME_PATHWAY	R-HSA-3700989~Transcriptional Regulation by TP53	5	0.375	0.99938814
REACTOME_PATHWAY	R-HSA-212436~Generic Transcription Pathway	12	0.263	1
REACTOME_PATHWAY	R-HSA-73857~RNA Polymerase II Transcription	12	0.239	1
REACTOME_PATHWAY	R-HSA-74160~Gene expression (Transcription)	12	0.217	
FE, Fold Enrichment				

Supplementary Table 3. Demographic, clinical, haematological and biochemical characteristics of Healthy Donors, $T2^{High}$ and $T2^{Low}$ asthma phenotypes

	HC	$T2^{High}$	$T2^{Low}$	p-value
**N**	4	4	4	
**Demographic characteristics**				
Age	58 (5.2)	60.25 (8.0)	62.33 (9.0)	0.958
Sex (M/F)	1/3	1/3	1/3	
**Haematological and biochemical variables**				
Eosinophils (x10^3/µL)	156.7 (95.04)	282.5 (212.0)*	86.67(25.17)	**0.008**
Neutrophils (x10^3/µL)	3337 (1241)	3055 (950.1)	3173 (1162)	0.971
Lymphocytes (x10^3/µL)	2093 (911.3)	1725 (686.4)	2093 (371.1)	0.716
Monocytes (x10^3/µL)	336.7 (151.4)	327.5 (35.9)	370.0 (70.0)	
Basophils (x10^3/µL)	30.00 (10.0)	40.00 (21.6)	33.33 (5.8)	0.917
TNF-α (pg/mL)	5.7(0.4)	8.700 (2.6)	6 (1.7)	0.159
**Pulmonary function**				
FEV1 (%)	-	84.25 (13.3)	113.0 (37.3)	0.400
FVC (%)	-	102.0 (11.7)	127.0 (30.5)	0.400
FEV1/FVC (%)	-	64.66 (9.5)	68.84 (9.5)	0.628
**Asthma biomarkers**				
IgE (IU/mL)	16.00 (1.4)	269.0 (188.2)	13.67 (12.4)	0.061
FeNO (ppb)	-	55.8 (53.4)	13.00 (11.3)	0.143

U-Mann Whitney test was used for comparisons between T2high and T2low. Kruskal-Wallis test followed by Dunn´s multiple comparison test was used in the three group comparisons. P-values are depicted. * p<0.05 in T2high vs T2low using Dunn´s multiple comparison test

Supplementary Table 4. Normalized urinary exosome-miRNA expression from Nanostring analyses

	T2low S1	T2low S2	T2low S3	T2high S1	T2high S2	HC S1	HC S2	HC S3
**hsa-let-7a-5p**	4,343	5,205	6,063	5,534	7,639	4,989	5,498	5,547
**hsa-let-7b-5p**	5,505	6,114	6,715	6,477	7,945	5,623	5,966	6,324
**hsa-let-7c-5p**	4,134	4,525	4,718	4,528	5,642	4,461	4,250	4,696
**hsa-let-7d-5p**	5,285	4,846	5,109	4,956	5,439	5,143	4,693	5,175
**hsa-let-7e-5p**	4,437	4,798	4,693	4,652	5,100	4,501	4,559	4,383
**hsa-let-7f-5p**	4,499	4,961	4,727	4,345	4,689	5,084	4,666	4,113
**hsa-let-7g-5p**	5,474	5,296	5,013	5,267	5,666	5,139	5,231	5,178
**hsa-miR-100-5p**	4,856	5,136	5,018	4,937	5,438	5,051	5,002	4,836
**hsa-miR-103a-3p**	5,108	4,658	4,629	4,659	4,033	4,555	4,430	4,266
**hsa-miR-107**	5,138	5,746	5,455	5,804	5,637	4,938	5,399	5,433
**hsa-miR-10a-5p**	4,530	5,182	6,153	5,535	6,408	5,049	5,359	5,427
**hsa-miR-10b-5p**	3,978	5,266	6,386	5,787	7,054	4,835	5,728	5,699
**hsa-miR-1178-3p**	4,575	4,555	4,427	4,852	4,686	4,377	4,984	5,032
**hsa-miR-1183**	6,192	6,060	6,423	6,285	6,046	5,908	6,621	5,753
**hsa-miR-1197**	4,661	4,671	4,804	4,722	4,024	4,915	4,554	5,079
**hsa-miR-122-5p**	4,758	4,378	4,951	5,115	4,811	4,337	4,610	5,445
**hsa-miR-1226-3p**	5,142	4,783	4,797	4,974	4,804	4,689	4,779	4,903
**hsa-miR-1234-3p**	4,396	4,916	4,564	4,708	4,627	4,905	4,867	5,027
**hsa-miR-1236-3p**	4,723	4,249	4,259	4,921	4,565	3,427	4,371	4,410
**hsa-miR-1245b-5p**	4,211	3,987	4,477	4,388	4,617	4,367	4,148	5,044
**hsa-miR-1246**	5,455	5,200	5,628	5,215	4,959	5,294	5,531	4,644
**hsa-miR-1253**	5,542	5,656	5,062	5,655	5,559	5,115	5,798	5,384
**hsa-miR-1254**	4,658	4,854	4,077	4,445	4,860	4,552	4,245	4,396
**hsa-miR-1255a**	4,646	5,110	3,682	4,402	4,742	4,808	4,386	4,681
**hsa-miR-1255b-5p**	4,551	4,882	4,699	4,525	4,439	5,085	5,043	4,540
**hsa-miR-125a-5p**	4,012	4,428	4,905	4,725	5,454	3,934	4,029	4,488
**hsa-miR-125b-5p**	3,946	5,123	6,373	5,309	7,118	4,998	5,091	5,143
**hsa-miR-1260a**	3,982	4,859	4,861	4,728	6,494	4,647	4,592	4,977
**hsa-miR-1261**	4,748	4,732	4,767	4,981	4,690	4,051	4,908	4,194
**hsa-miR-1264**	4,716	5,394	4,956	5,142	5,383	5,008	5,259	5,026
**hsa-miR-1268b**	4,908	4,697	4,889	4,892	4,696	4,792	5,085	4,896
**hsa-miR-1269a**	5,442	5,615	5,366	5,545	5,436	5,297	5,480	5,314
**hsa-miR-127-3p**	5,013	4,979	4,877	5,044	5,304	5,007	5,050	5,276
**hsa-miR-1270**	4,875	5,046	5,029	4,978	4,903	4,789	4,986	4,587
**hsa-miR-1272**	5,095	4,310	4,580	4,342	4,197	4,766	4,322	4,323
**hsa-miR-1273c**	5,187	5,068	4,809	4,939	4,565	4,698	4,676	4,416
**hsa-miR-1283**	1,556	5,068	3,923	3,621	2,094	3,047	4,113	1,508
**hsa-miR-1285-3p**	4,844	5,134	5,262	5,029	4,694	5,375	5,145	5,267

**hsa-miR-1285-5p**	4,821	5,412	5,594	5,515	5,847	4,960	5,518	5,171
**hsa-miR-1286**	5,525	5,801	5,833	5,777	4,948	5,697	5,662	5,715
**hsa-miR-1287-5p**	4,874	5,186	4,926	5,111	5,000	4,792	5,190	4,796
**hsa-miR-1288-3p**	5,068	4,243	4,680	4,469	4,284	4,600	4,801	4,225
**hsa-miR-1289**	4,239	5,051	5,073	4,776	4,568	5,018	5,019	4,748
**hsa-miR-129-2-3p**	5,001	4,503	4,569	4,650	4,499	4,742	4,289	4,977
**hsa-miR-1293**	4,762	5,040	4,998	4,767	4,688	5,205	5,027	4,766
**hsa-miR-1295a**	5,036	5,302	5,324	5,255	4,953	5,089	5,278	4,970
**hsa-miR-1296-3p**	4,421	4,489	4,788	4,285	4,741	5,112	5,102	4,454
**hsa-miR-1296-5p**	4,452	5,261	4,961	4,689	5,136	5,262	4,803	4,713
**hsa-miR-1297**	5,010	4,677	4,772	4,611	4,902	5,138	4,685	4,933
**hsa-miR-1299**	5,160	4,689	4,824	4,778	4,855	5,425	5,349	5,413
**hsa-miR-1302**	4,790	5,082	3,744	4,494	4,994	4,655	4,571	4,510
**hsa-miR-1304-3p**	5,464	5,100	4,992	5,244	4,685	4,819	5,097	4,874
**hsa-miR-1304-5p**	4,872	4,467	4,504	4,650	4,749	4,849	5,423	4,911
**hsa-miR-1305**	4,818	5,059	4,857	4,921	4,954	4,764	5,033	4,570
**hsa-miR-1307-5p**	5,417	5,139	5,401	5,337	4,852	5,288	5,138	5,453
**hsa-miR-1322**	4,822	4,902	5,176	5,015	4,909	4,691	4,898	4,653
**hsa-miR-134-5p+hsa-miR-6728-5p**	4,999	4,793	4,632	4,324	5,000	4,995	3,874	4,240
**hsa-miR-136-5p**	4,707	5,064	4,939	4,616	4,502	5,137	4,794	4,387
**hsa-miR-138-5p**	5,011	5,394	5,496	5,377	4,951	5,359	5,147	5,516
**hsa-miR-139-3p**	5,046	5,352	4,642	5,035	4,952	4,431	4,682	4,150
**hsa-miR-140-3p**	5,051	5,142	4,772	5,086	4,621	4,510	5,014	4,410
**hsa-miR-141-3p**	5,151	5,942	5,763	5,727	5,135	5,461	5,487	5,412
**hsa-miR-143-3p**	4,532	5,052	4,672	4,657	4,129	4,804	4,926	4,279
**hsa-miR-145-5p**	5,239	4,662	4,665	4,862	4,955	4,740	4,856	4,886
**hsa-miR-1469**	4,447	4,817	4,965	4,379	4,126	4,862	4,635	3,812
**hsa-miR-146a-5p**	5,110	4,718	4,802	4,798	5,043	5,006	5,002	4,963
**hsa-miR-147a**	4,809	4,670	4,949	4,806	4,801	4,890	5,178	4,831
**hsa-miR-149-5p**	4,807	5,037	4,786	4,773	5,409	4,843	5,128	4,394
**hsa-miR-153-3p**	4,556	4,522	4,052	4,700	4,355	4,215	4,767	4,848
**hsa-miR-1537-3p**	4,607	4,784	4,887	4,762	5,324	4,781	4,588	4,856
**hsa-miR-155-5p**	5,403	4,758	4,729	4,927	4,566	4,677	5,438	4,394
**hsa-miR-16-5p**	4,552	4,772	4,549	4,517	5,293	4,999	4,629	4,906
**hsa-miR-181a-2-3p**	4,311	4,284	4,413	4,566	4,847	4,345	4,314	5,038
**hsa-miR-181a-5p**	4,534	4,524	4,696	4,590	5,480	4,731	4,433	4,971
**hsa-miR-181d-3p**	5,028	5,189	4,678	5,202	5,136	5,022	5,823	5,311
**hsa-miR-182-3p**	4,825	4,691	4,031	4,470	4,273	4,152	3,931	4,057
**hsa-miR-1827**	5,226	5,078	5,415	5,365	4,911	5,060	5,148	5,358
**hsa-miR-183-5p**	5,237	5,169	5,231	5,332	5,130	4,926	5,065	5,155
**hsa-miR-186-5p**	5,036	5,502	5,157	5,442	5,258	5,132	5,035	5,570
**hsa-miR-188-5p**	5,272	5,467	5,646	5,463	5,638	5,748	5,848	5,721

**hsa-miR-191-5p**	3,829	4,765	4,962	4,573	6,216	4,555	5,107	4,303
**hsa-miR-193a-3p**	5,017	4,749	4,940	4,982	5,049	4,853	5,267	4,998
**hsa-miR-195-5p**	4,702	4,387	5,028	4,542	4,956	5,312	4,910	5,286
**hsa-miR-196a-5p**	5,124	5,271	4,700	4,969	5,329	5,209	5,064	5,098
**hsa-miR-197-3p**	5,176	5,010	4,613	4,982	5,093	4,667	4,600	4,835
**hsa-miR-197-5p**	4,910	4,742	4,502	4,511	5,101	5,251	4,691	5,105
**hsa-miR-1972**	5,319	5,743	6,065	5,811	6,037	5,781	5,693	6,001
**hsa-miR-1976**	4,782	4,756	5,028	4,764	4,852	4,939	4,746	4,796
**hsa-miR-198**	5,318	5,316	5,269	5,259	5,262	5,486	5,610	5,355
**hsa-miR-199a-3p+hsa-miR-199b-3p**	4,883	4,678	5,040	4,731	4,625	4,822	4,382	4,672
**hsa-miR-199a-5p**	5,607	5,456	5,500	5,554	5,405	5,387	5,417	5,479
**hsa-miR-199b-5p**	4,746	4,782	4,450	4,789	4,902	4,909	4,937	5,307
**hsa-miR-200a-3p**	4,552	4,045	4,958	4,520	5,226	4,414	4,632	4,542
**hsa-miR-200c-3p**	4,585	5,472	5,408	5,509	6,385	4,739	4,980	5,317
**hsa-miR-202-3p**	5,106	4,136	4,529	4,079	4,376	4,942	4,283	4,194
**hsa-miR-203a-3p**	4,918	4,843	4,977	4,512	4,626	5,158	4,610	4,337
**hsa-miR-204-5p**	4,853	5,256	6,846	6,081	7,192	4,867	5,667	5,555
**hsa-miR-2053**	4,989	4,400	4,997	4,816	4,436	5,067	5,109	5,342
**hsa-miR-206**	4,513	4,544	5,199	4,946	4,627	4,390	4,675	4,718
**hsa-miR-208b-3p**	5,375	5,018	5,094	5,182	5,640	5,148	5,062	5,389
**hsa-miR-210-5p**	4,956	4,963	4,756	4,488	4,899	5,025	3,899	4,395
**hsa-miR-211-5p**	4,640	4,757	4,508	4,942	4,908	4,248	4,720	4,796
**hsa-miR-2110**	5,007	4,773	4,708	4,985	5,004	4,589	4,908	4,927
**hsa-miR-212-3p**	5,423	6,221	5,481	5,845	5,694	5,773	5,704	5,913
**hsa-miR-216a-5p**	5,913	5,719	5,749	5,740	5,174	5,602	5,543	5,457
**hsa-miR-216b-5p**	5,435	4,909	4,890	5,064	4,563	4,764	5,244	4,602
**hsa-miR-217**	4,775	5,163	4,878	4,944	4,996	4,976	5,166	4,790
**hsa-miR-219b-3p**	4,648	4,694	5,052	4,916	5,045	4,900	4,756	5,312
**hsa-miR-223-3p**	4,767	4,898	3,875	4,615	4,284	4,094	4,862	3,900
**hsa-miR-224-5p**	5,035	4,616	5,415	4,698	4,691	5,051	5,113	4,270
**hsa-miR-23a-3p**	4,990	5,587	6,287	5,884	7,374	5,255	5,458	5,757
**hsa-miR-23b-3p**	4,564	4,727	4,900	4,897	5,347	4,725	5,118	5,016
**hsa-miR-23c**	4,677	5,363	5,019	5,073	4,852	5,288	5,087	5,324
**hsa-miR-25-3p**	4,205	5,053	4,720	4,779	5,184	4,458	3,952	4,698
**hsa-miR-28-3p**	4,474	4,684	5,350	4,936	4,902	5,047	5,417	5,159
**hsa-miR-28-5p**	5,465	5,753	5,228	5,431	5,402	5,562	5,626	5,303
**hsa-miR-296-3p**	4,793	4,731	4,519	4,721	4,357	4,866	5,196	4,876
**hsa-miR-299-5p**	5,174	4,717	5,182	5,094	4,801	4,784	4,668	5,114
**hsa-miR-29b-3p**	4,742	4,287	4,584	4,429	5,295	4,872	4,685	4,933
**hsa-miR-29c-3p**	4,602	4,610	4,963	4,740	5,404	4,783	4,761	4,897
**hsa-miR-301a-3p**	4,993	4,643	5,320	4,762	4,119	5,367	4,924	5,077
**hsa-miR-301a-5p**	4,968	5,084	4,928	5,120	5,374	5,112	5,469	5,327

**hsa-miR-301b-3p**	4,879	4,712	4,774	4,907	5,291	4,866	5,481	5,054
**hsa-miR-301b-5p**	4,879	4,765	5,236	4,886	4,907	5,168	4,961	5,134
**hsa-miR-302a-5p**	5,017	4,691	5,097	5,110	4,901	4,756	5,246	5,112
**hsa-miR-302b-3p**	5,088	4,801	4,422	4,729	4,425	5,164	5,012	5,260
**hsa-miR-302c-3p**	5,030	4,685	4,742	4,693	4,628	4,713	4,603	4,490
**hsa-miR-302d-3p**	7,085	6,930	7,100	6,852	6,875	7,392	7,466	6,966
**hsa-miR-302e**	5,251	5,020	4,960	4,649	5,048	5,409	5,558	4,320
**hsa-miR-30a-3p**	4,600	4,441	4,441	4,850	5,191	3,976	4,530	4,724
**hsa-miR-30a-5p**	4,441	5,134	6,098	5,186	6,532	5,082	5,122	4,808
**hsa-miR-30b-5p**	5,152	5,439	5,565	5,576	5,775	5,121	5,821	5,239
**hsa-miR-30d-5p**	4,345	5,723	7,019	5,822	7,633	5,541	5,810	5,447
**hsa-miR-30e-3p**	4,677	4,795	5,056	4,888	5,487	4,474	4,547	4,504
**hsa-miR-30e-5p**	4,327	4,651	4,462	4,611	5,185	4,401	4,565	4,619
**hsa-miR-3131**	4,865	4,782	4,242	4,732	4,495	4,353	5,061	4,299
**hsa-miR-3144-3p**	4,835	4,346	4,581	4,700	4,685	4,776	5,145	5,151
**hsa-miR-3147**	4,756	5,114	4,539	4,844	4,618	4,811	5,029	4,694
**hsa-miR-3161**	5,159	5,247	4,823	5,087	4,907	5,067	5,216	4,985
**hsa-miR-3180**	5,320	5,201	5,239	5,401	5,136	5,008	5,184	5,320
**hsa-miR-3180-5p**	4,497	4,765	4,312	4,466	4,212	4,417	4,631	4,026
**hsa-miR-3182**	5,124	4,402	4,933	4,787	4,851	4,674	4,682	4,802
**hsa-miR-320a**	4,388	4,606	4,428	4,603	4,353	4,448	4,776	4,581
**hsa-miR-320c**	5,510	5,148	5,164	5,208	4,799	5,314	5,409	5,189
**hsa-miR-320d**	4,980	4,567	4,470	4,646	4,955	4,737	4,916	4,763
**hsa-miR-320e**	4,745	7,834	6,252	6,400	6,489	5,829	4,942	5,392
**hsa-miR-323a-3p**	4,799	4,638	4,451	4,420	4,276	4,960	5,078	4,435
**hsa-miR-323b-3p**	4,732	4,752	4,924	4,733	4,686	4,896	4,535	4,857
**hsa-miR-324-3p**	4,961	4,884	4,511	4,677	4,907	4,942	5,212	4,598
**hsa-miR-324-5p**	4,910	4,911	4,342	4,634	4,904	4,646	4,605	4,413
**hsa-miR-325**	5,297	5,320	5,260	5,277	5,043	5,367	5,430	5,291
**hsa-miR-337-3p**	4,771	4,447	4,318	4,627	5,248	4,865	4,862	5,417
**hsa-miR-337-5p**	4,937	4,839	4,839	4,973	5,049	4,682	4,732	4,945
**hsa-miR-340-5p**	5,558	5,319	5,279	5,415	5,255	5,193	5,050	5,360
**hsa-miR-342-3p**	5,129	4,450	4,986	4,881	5,260	4,212	4,081	4,466
**hsa-miR-342-5p**	4,657	4,791	4,834	4,833	4,746	4,630	4,794	4,690
**hsa-miR-345-5p**	5,143	4,924	4,594	4,766	4,946	4,838	5,178	4,429
**hsa-miR-346**	5,190	5,000	5,162	5,092	5,135	5,081	4,678	5,236
**hsa-miR-34a-5p**	4,833	5,351	5,015	5,045	5,262	5,334	5,426	5,131
**hsa-miR-34b-3p**	4,498	4,522	4,482	4,624	4,682	3,789	4,119	3,847
**hsa-miR-361-3p**	5,013	4,985	4,883	4,805	5,479	4,862	4,662	4,542
**hsa-miR-3613-3p**	5,368	4,511	4,601	5,013	4,629	4,563	5,024	5,106
**hsa-miR-3614-5p**	5,348	5,154	5,321	5,236	5,044	5,370	5,201	5,405
**hsa-miR-3615**	4,737	4,837	4,505	4,949	5,005	4,479	4,719	5,023

**hsa-miR-363-3p**	5,712	6,178	6,041	6,185	5,880	5,601	5,972	5,778
**hsa-miR-365a-3p+hsa-miR-365b-3p**	5,529	4,962	5,398	5,187	5,539	5,533	5,461	5,528
**hsa-miR-365b-5p**	4,242	4,908	4,594	4,582	4,689	5,101	4,967	5,046
**hsa-miR-367-3p**	5,023	4,623	4,845	4,803	4,954	4,729	4,448	4,893
**hsa-miR-369-3p**	5,213	5,116	4,750	5,024	5,380	5,139	4,821	5,339
**hsa-miR-3690**	4,911	4,581	5,130	4,654	5,130	4,898	5,007	4,393
**hsa-miR-370-3p**	5,225	4,992	5,075	5,127	5,214	5,159	5,058	5,381
**hsa-miR-374b-5p**	4,991	4,697	4,685	4,621	4,033	4,861	4,715	4,510
**hsa-miR-374c-5p**	4,666	4,847	4,755	4,669	4,577	4,577	4,307	4,336
**hsa-miR-376a-3p**	5,130	5,307	5,619	5,457	5,580	5,531	5,452	5,851
**hsa-miR-376c-3p**	4,450	4,934	4,439	4,311	4,674	5,147	4,604	4,504
**hsa-miR-377-3p**	4,767	4,584	4,480	4,390	4,365	5,264	5,002	4,944
**hsa-miR-378d**	4,980	4,348	4,538	4,735	4,746	4,559	4,364	5,159
**hsa-miR-378e**	5,773	5,283	5,447	5,496	5,495	5,504	5,529	5,649
**hsa-miR-378f**	4,554	4,893	4,838	4,832	4,700	4,875	4,459	5,143
**hsa-miR-378g**	5,072	4,433	4,859	4,593	4,627	5,138	5,086	4,907
**hsa-miR-379-5p**	5,020	5,206	4,604	5,042	5,094	4,622	4,796	4,695
**hsa-miR-382-5p**	5,120	4,854	4,584	4,914	4,630	5,073	5,260	5,280
**hsa-miR-384**	5,245	4,846	4,903	4,936	4,443	5,171	5,212	5,115
**hsa-miR-3928-3p**	4,976	4,576	5,069	4,825	5,098	4,950	4,570	5,143
**hsa-miR-3934-5p**	4,605	4,829	5,245	4,889	4,843	5,035	4,730	5,103
**hsa-miR-409-3p**	4,942	4,680	4,549	5,048	4,365	4,514	4,835	5,289
**hsa-miR-412-3p**	4,656	5,097	4,241	4,726	4,911	4,811	4,824	4,865
**hsa-miR-421**	5,343	5,143	5,430	5,284	5,572	5,572	5,047	5,873
**hsa-miR-423-3p**	4,996	4,732	4,752	4,806	4,740	4,902	4,831	4,973
**hsa-miR-423-5p**	5,240	5,506	5,083	5,279	5,006	5,482	5,292	5,496
**hsa-miR-4286**	5,099	5,503	5,717	5,640	8,747	5,640	5,864	6,254
**hsa-miR-432-5p**	4,888	4,535	4,839	4,668	4,685	4,906	4,755	4,881
**hsa-miR-4421**	5,148	4,737	4,686	4,986	4,360	4,761	4,804	5,178
**hsa-miR-4425**	4,882	4,943	5,693	4,847	4,798	5,138	5,241	4,157
**hsa-miR-4431**	4,921	5,436	4,968	4,928	4,748	5,253	5,213	4,600
**hsa-miR-4443**	4,903	5,188	4,850	4,941	5,666	5,194	4,976	5,169
**hsa-miR-4454+hsa-miR-7975**	5,207	7,707	8,150	8,403	13,530	6,803	7,965	9,729
**hsa-miR-4461**	5,007	4,214	4,825	4,746	4,902	4,727	4,988	5,097
**hsa-miR-4488**	4,876	4,801	5,104	5,014	4,908	4,668	4,584	4,916
**hsa-miR-449c-5p**	4,370	4,863	4,931	4,856	4,362	4,703	5,020	4,749
**hsa-miR-450a-1-3p**	4,529	4,338	4,735	4,143	4,111	4,957	4,650	4,136
**hsa-miR-4516**	4,332	5,944	5,006	5,858	7,545	5,014	4,567	6,905
**hsa-miR-451a**	4,955	4,555	4,307	4,587	4,490	4,599	4,507	4,705
**hsa-miR-4536-3p**	4,951	4,907	5,237	5,183	4,853	4,651	4,864	4,906
**hsa-miR-4536-5p**	4,755	5,326	5,284	5,401	5,174	4,841	4,942	5,319
**hsa-miR-4755-5p**	5,038	4,757	4,964	4,819	4,435	4,787	4,845	4,521

**hsa-miR-4787-3p**	4,742	4,944	4,443	4,327	4,563	4,919	4,104	4,158
**hsa-miR-484**	5,177	4,974	4,331	5,013	4,905	4,477	4,976	4,773
**hsa-miR-488-3p**	4,460	3,899	4,567	4,023	3,310	5,124	4,288	4,975
**hsa-miR-489-3p**	4,983	5,761	5,200	5,138	5,089	5,427	5,579	4,619
**hsa-miR-491-5p**	5,361	3,969	5,257	4,714	4,683	4,865	4,629	5,026
**hsa-miR-493-3p**	5,218	4,710	4,860	4,948	5,182	4,791	5,012	4,913
**hsa-miR-494-3p**	4,852	4,515	4,893	4,895	4,746	4,620	4,596	5,109
**hsa-miR-495-3p**	5,777	6,038	5,728	5,942	5,171	5,722	5,668	5,835
**hsa-miR-496**	5,005	5,129	5,070	5,259	5,313	4,734	5,099	5,016
**hsa-miR-499a-5p**	4,566	4,904	4,337	4,603	4,360	5,059	4,673	5,175
**hsa-miR-499b-3p**	4,622	4,541	4,638	4,544	4,739	4,459	4,019	4,492
**hsa-miR-5010-3p**	4,670	4,528	4,983	4,758	4,739	4,413	4,221	4,564
**hsa-miR-502-3p**	4,298	4,755	4,143	4,292	4,348	5,004	4,517	4,872
**hsa-miR-502-5p**	4,948	4,454	4,918	4,682	4,623	4,666	4,477	4,630
**hsa-miR-503-3p**	4,670	4,637	4,560	4,610	4,622	4,848	4,894	4,853
**hsa-miR-505-3p**	4,784	4,713	4,763	4,871	4,564	4,787	4,747	5,145
**hsa-miR-506-3p**	4,974	4,789	4,876	4,742	4,506	4,957	4,495	4,819
**hsa-miR-506-5p**	4,870	5,003	4,988	5,135	4,862	4,656	5,135	4,845
**hsa-miR-508-5p**	4,890	4,950	5,034	4,880	5,260	5,098	4,520	5,151
**hsa-miR-509-3-5p**	5,259	5,214	4,806	4,955	4,997	5,162	5,058	4,842
**hsa-miR-509-5p**	5,023	4,216	4,188	4,375	4,750	4,579	4,342	4,710
**hsa-miR-513a-3p**	5,209	4,593	4,842	4,699	4,684	5,375	5,151	5,265
**hsa-miR-513a-5p**	4,535	4,955	4,451	4,500	4,201	5,041	4,206	4,880
**hsa-miR-513c-5p**	4,968	5,083	5,536	5,338	4,907	5,021	5,717	5,039
**hsa-miR-514a-3p**	4,883	4,730	4,586	4,908	4,568	4,823	5,018	5,281
**hsa-miR-514b-5p**	5,534	5,336	5,397	5,309	4,747	5,427	5,169	5,238
**hsa-miR-516b-5p**	4,746	4,418	5,140	4,864	4,999	4,709	5,244	4,878
**hsa-miR-517a-3p**	5,108	5,277	5,338	5,113	5,223	5,191	5,160	4,787
**hsa-miR-518c-3p**	5,093	4,824	4,206	4,365	4,563	4,814	4,878	3,940
**hsa-miR-5196-5p**	4,641	4,580	4,941	4,685	4,999	4,916	5,147	4,811
**hsa-miR-519c-3p**	5,369	5,291	5,703	5,546	5,644	5,584	5,953	5,759
**hsa-miR-519d-3p**	4,869	5,427	4,820	5,006	4,682	5,138	5,115	4,856
**hsa-miR-520a-5p**	4,995	4,597	4,862	4,756	4,500	4,958	4,866	4,968
**hsa-miR-520c-3p**	4,263	5,129	5,016	4,630	4,896	4,840	4,234	4,336
**hsa-miR-520d-3p**	4,954	4,296	4,359	4,559	4,804	4,555	5,062	4,657
**hsa-miR-520f-3p**	4,896	4,951	4,831	4,571	4,571	4,946	4,420	4,243
**hsa-miR-520h**	4,868	5,006	4,758	5,013	4,199	4,799	4,703	5,093
**hsa-miR-522-3p**	4,670	5,157	4,607	4,982	4,437	4,717	4,642	4,997
**hsa-miR-525-5p**	4,526	4,971	3,989	4,658	5,128	4,692	4,967	4,953
**hsa-miR-526a+hsa-miR-518c-5p+hsa-miR-518d-5p**	5,077	5,274	4,919	4,899	4,799	5,358	4,926	4,972
**hsa-miR-526b-5p**	5,481	5,278	4,922	5,182	5,334	4,992	4,927	4,917

**hsa-miR-542-3p**	4,852	4,922	4,727	4,857	4,955	4,994	4,915	5,108
**hsa-miR-543**	5,226	4,975	5,149	5,200	5,304	5,154	5,284	5,437
**hsa-miR-548aa+hsa-miR-548t-3p**	4,903	4,601	4,465	4,808	4,735	4,388	4,422	4,840
**hsa-miR-548ah-5p**	5,464	5,232	5,311	5,149	5,253	5,566	5,261	5,300
**hsa-miR-548al**	5,345	5,250	5,470	5,425	5,369	5,038	4,880	5,263
**hsa-miR-548ar-3p**	5,131	4,910	4,995	4,936	4,999	5,129	5,046	5,053
**hsa-miR-548ar-5p**	5,879	5,696	5,959	5,792	5,742	5,834	5,696	5,798
**hsa-miR-548d-5p**	4,822	4,992	5,156	4,942	4,627	4,755	4,373	4,649
**hsa-miR-548e-5p**	5,819	5,476	5,732	5,767	5,096	5,773	5,537	6,214
**hsa-miR-548i**	4,411	4,481	4,382	4,581	3,923	3,884	4,142	4,030
**hsa-miR-548j-3p**	5,040	4,705	4,815	4,774	4,744	4,797	4,196	4,918
**hsa-miR-548k**	4,825	4,793	5,328	5,156	5,407	4,901	5,703	5,084
**hsa-miR-548n**	4,013	4,977	4,421	4,582	4,354	5,008	4,837	5,157
**hsa-miR-548v**	4,896	4,923	4,840	4,968	5,260	5,127	5,313	5,345
**hsa-miR-548z+hsa-miR-548h-3p**	5,016	4,758	4,607	4,977	4,863	4,392	5,029	4,642
**hsa-miR-556-5p**	4,322	4,494	4,172	4,523	3,822	4,024	4,710	4,112
**hsa-miR-566**	5,002	4,560	5,027	4,765	5,091	5,157	4,746	5,288
**hsa-miR-574-5p**	4,833	5,399	4,778	5,049	5,236	5,479	6,679	5,038
**hsa-miR-575**	4,568	4,433	5,320	4,593	5,178	5,008	4,674	4,783
**hsa-miR-576-3p**	4,447	4,800	4,164	4,543	4,898	4,784	4,563	5,046
**hsa-miR-576-5p**	5,074	4,991	5,257	4,749	4,495	5,159	4,217	4,555
**hsa-miR-577**	4,777	5,114	4,999	4,767	5,052	5,017	4,898	4,431
**hsa-miR-579-5p**	4,925	4,050	4,318	4,254	4,635	4,975	4,825	4,976
**hsa-miR-580-3p**	4,849	4,373	4,729	4,685	5,089	4,323	4,758	4,368
**hsa-miR-584-3p**	5,280	5,186	5,269	5,324	5,219	5,114	5,180	5,309
**hsa-miR-584-5p**	4,977	5,067	5,297	5,117	5,049	5,216	5,126	5,251
**hsa-miR-585-3p**	4,814	5,181	4,478	4,818	4,848	4,954	4,478	5,027
**hsa-miR-587**	4,879	4,622	5,175	4,622	4,902	5,144	4,683	4,737
**hsa-miR-590-5p**	4,488	5,696	5,331	5,336	4,900	5,136	5,466	5,017
**hsa-miR-598-3p**	6,086	6,833	6,541	6,561	6,220	6,209	6,431	5,991
**hsa-miR-603**	5,299	4,897	4,675	5,078	4,945	4,948	5,232	5,296
**hsa-miR-604**	4,733	4,450	4,502	4,916	4,744	3,970	4,290	4,795
**hsa-miR-605-5p**	5,281	5,289	5,545	5,210	5,255	5,437	5,421	5,006
**hsa-miR-607**	4,698	5,111	4,948	5,019	5,332	4,703	4,880	4,751
**hsa-miR-610**	4,898	4,759	4,410	4,566	5,264	4,890	4,833	4,702
**hsa-miR-612**	4,716	4,753	4,775	4,524	4,693	5,356	5,286	4,881
**hsa-miR-613**	4,904	4,930	4,734	4,539	4,909	5,292	4,951	4,645
**hsa-miR-615-3p**	4,769	4,732	4,326	4,771	4,355	4,708	4,631	5,194
**hsa-miR-617**	4,456	4,864	4,779	4,695	4,738	4,610	4,903	4,325
**hsa-miR-625-5p**	5,018	5,014	4,667	4,924	4,638	4,748	5,030	4,645
**hsa-miR-626**	4,829	4,865	4,847	4,724	5,186	4,897	4,693	4,664
**hsa-miR-627-3p**	5,084	3,713	4,934	4,597	4,794	4,584	4,954	4,982

**hsa-miR-627-5p**	4,876	4,656	4,888	4,611	5,086	5,002	5,068	4,578
**hsa-miR-630**	4,150	4,902	5,217	4,766	6,011	4,807	4,428	4,844
**hsa-miR-631**	4,303	5,107	4,684	4,715	4,683	4,768	4,765	4,554
**hsa-miR-639**	4,663	4,951	4,684	4,712	5,043	4,662	4,309	4,555
**hsa-miR-640**	4,829	5,376	4,818	4,877	4,564	4,791	5,091	4,039
**hsa-miR-648**	5,146	4,183	4,173	4,182	4,565	4,653	3,985	4,409
**hsa-miR-649**	4,426	4,635	5,318	4,405	4,748	5,286	5,017	4,371
**hsa-miR-6503-3p**	5,084	4,637	4,136	4,509	4,568	4,878	4,788	4,806
**hsa-miR-6503-5p**	4,876	4,756	4,943	4,856	4,847	4,779	4,775	4,799
**hsa-miR-651-5p**	5,420	5,416	5,074	5,337	4,959	5,182	5,244	5,194
**hsa-miR-654-3p**	4,912	4,982	4,628	4,842	4,804	5,009	5,010	5,017
**hsa-miR-654-5p**	5,213	4,913	4,750	4,982	4,695	4,570	4,989	4,462
**hsa-miR-655-3p**	5,169	4,637	4,449	4,549	4,797	4,862	4,817	4,526
**hsa-miR-656-3p**	5,113	5,195	4,753	5,162	5,222	4,998	4,720	5,476
**hsa-miR-660-3p**	4,930	4,498	4,458	4,769	4,434	4,812	4,791	5,375
**hsa-miR-664a-3p**	4,946	5,052	5,189	5,200	5,335	4,826	5,133	5,014
**hsa-miR-665**	4,665	4,790	4,717	4,740	4,634	4,790	5,050	4,695
**hsa-miR-671-5p**	4,951	4,476	4,053	4,316	4,369	4,787	4,404	4,660
**hsa-miR-6721-5p**	5,414	4,764	5,351	5,040	5,181	4,887	4,905	4,670
**hsa-miR-758-3p+hsa-miR-411-3p**	5,649	5,134	5,215	5,215	4,567	5,258	5,353	4,998
**hsa-miR-758-5p**	4,648	4,280	5,247	4,421	4,127	4,942	4,408	4,460
**hsa-miR-760**	4,433	4,867	4,984	4,631	4,630	4,697	4,180	4,381
**hsa-miR-761**	5,396	5,086	5,256	5,131	5,176	5,190	5,284	4,925
**hsa-miR-764**	4,485	4,705	4,959	4,893	5,044	4,514	4,884	4,762
**hsa-miR-765**	4,975	4,745	5,081	4,838	4,747	5,223	5,074	5,151
**hsa-miR-769-5p**	4,623	4,784	4,865	4,758	4,215	4,831	4,544	4,872
**hsa-miR-770-5p**	4,455	4,816	5,005	4,699	4,277	4,440	4,024	4,224
**hsa-miR-873-3p**	7,200	7,566	7,678	7,497	7,267	7,098	7,386	6,805
**hsa-miR-876-3p**	5,077	4,801	4,915	4,772	4,631	5,217	5,066	4,973
**hsa-miR-887-5p**	4,448	5,136	4,256	4,656	5,041	4,574	4,126	4,653
**hsa-miR-892a**	4,444	4,763	5,139	4,802	4,854	4,728	4,668	4,701
**hsa-miR-892b**	4,408	4,778	4,861	4,769	5,059	4,642	5,342	4,492
**hsa-miR-92a-3p**	5,279	4,415	4,951	4,629	5,408	4,983	4,366	4,894
**hsa-miR-92b-3p**	4,720	4,383	4,727	4,851	4,365	4,774	4,709	5,597
**hsa-miR-93-5p**	5,219	4,961	5,369	5,080	5,055	5,125	5,236	4,865
**hsa-miR-933**	5,176	4,699	4,531	4,610	4,201	4,941	4,621	4,687
**hsa-miR-936**	4,561	4,786	5,018	4,885	4,438	4,851	5,099	4,932
**hsa-miR-939-5p**	5,432	5,244	5,176	5,167	5,100	5,266	4,937	5,122
**hsa-miR-96-5p**	4,821	5,084	4,757	4,824	5,330	5,252	5,260	5,093
**hsa-miR-99a-5p**	3,725	4,488	5,945	4,706	6,409	4,742	4,517	4,691
**hsa-miR-99b-5p**	5,317	5,426	5,306	5,262	6,010	5,342	5,064	5,222

**LIG_POS_A**	12,950	13,102	13,013	12,996	13,135	13,092	13,002	13,032
**LIG_POS_B**	10,009	10,146	10,108	10,063	10,157	10,150	10,084	10,081
**LIG_POS_C**	7,731	7,579	7,649	7,743	7,815	7,551	7,872	7,709

Supplementary Table 5. Targets for miRNAs up-regulated in $T2^{high}$ vs. T2low urinary exosomes.		
**Entrezid**	**Genesymbol**	**Description**
**158**	ADSL	Homo sapiens adenylosuccinate lyase (ADSL), transcript variant 1,mRNA.
**367**	AR	Homo sapiens androgen receptor (AR), transcript variant 1, mRNA.
**1029**	CDKN2A	Homo sapiens cyclin dependent kinase inhibitor 2A (CDKN2A),transcript variant 1, mRNA.
**1289**	COL5A1	Homo sapiens collagen type V alpha 1 chain (COL5A1), transcriptvariant 1, mRNA.
**2395**	FXN	Homo sapiens frataxin (FXN), transcript variant 1, mRNA; nucleargene for mitochondrial product.
**2710**	GK	Homo sapiens glycerol kinase (GK), transcript variant 2, mRNA.
**2908**	NR3C1	Homo sapiens nuclear receptor subfamily 3 group C member 1 (NR3C1),transcript variant 1, mRNA.
**3690**	ITGB3	Homo sapiens integrin subunit beta 3 (ITGB3), mRNA.
**3757**	KCNH2	Homo sapiens potassium voltage-gated channel subfamily H member 2(KCNH2), transcript variant 1, mRNA.
**4771**	NF2	Homo sapiens NF2, moesin-ezrin-radixin like (MERLIN) tumorsuppressor (NF2), transcript variant 1, mRNA.
**5213**	PFKM	Homo sapiens phosphofructokinase, muscle (PFKM), transcript variant4, mRNA.
**5230**	1 PGK	Homo sapiens phosphoglycerate kinase 1 (PGK1), mRNA.
**5728**	PTEN	Homo sapiens phosphatase and tensin homolog (PTEN), transcriptvariant 1, mRNA.
**6310**	ATXN1	Homo sapiens ataxin 1 (ATXN1), transcript variant 1, mRNA.
**6949**	TCOF1	Homo sapiens treacle ribosome biogenesis factor 1 (TCOF1),transcript variant 2, mRNA.
**7248**	TSC1	Homo sapiens TSC complex subunit 1 (TSC1), transcript variant 1,mRNA.
**1026**	CDKN1A	Homo sapiens cyclin dependent kinase inhibitor 1A (CDKN1A),transcript variant 1, mRNA.
**1536**	CYBB	Homo sapiens cytochrome b-245 beta chain (CYBB), mRNA.
**3077**	HFE	Homo sapiens homeostatic iron regulator (HFE), transcript variant1, mRNA.
**5077**	PAX3	Homo sapiens paired box 3 (PAX3), transcript variant PAX3A, mRNA.
**6821**	SUOX	Homo sapiens sulfite oxidase (SUOX), transcript variant 1, mRNA;nuclear gene for mitochondrial product.
**351**	APP	Homo sapiens amyloid beta precursor protein (APP), transcriptvariant 1, mRNA.
**546**	ATRX	Homo sapiens ATRX chromatin remodeler (ATRX), transcript variant 1,mRNA.
**3239**	HOXD13	Homo sapiens homeobox D13 (HOXD13), mRNA.
**3949**	LDLR	Homo sapiens low density lipoprotein receptor (LDLR), transcriptvariant 1, mRNA.
**7157**	TP53	Homo sapiens tumor protein p53 (TP53), transcript variant 1, mRNA.
**1406**	CRX	Homo sapiens cone-rod homeobox (CRX), mRNA.
**3570**	IL6R	Homo sapiens interleukin 6 receptor (IL6R), transcript variant 1,mRNA.
**2215**	FCGR3B	Homo sapiens Fc gamma receptor IIIb (FCGR3B), transcript variant 2,mRNA.
**1234**	CCR5	Homo sapiens C-C motif chemokine receptor 5 (CCR5), transcriptvariant A, mRNA.
**3485**	IGFBP2	Homo sapiens insulin like growth factor binding protein 2 (IGFBP2),transcript variant 1, mRNA.
**3488**	IGFBP5	Homo sapiens insulin like growth factor binding protein 5 (IGFBP5),mRNA.
**5054**	SERPINE1	Homo sapiens serpin family E member 1 (SERPINE1), transcriptvariant 2, mRNA.
**960**	CD44	Homo sapiens CD44 molecule (Indian blood group) (CD44), transcriptvariant 1, mRNA.
**966**	CD59	Homo sapiens CD59 molecule (CD59 blood group) (CD59), transcriptvariant 2, mRNA.
**4891**	SLC11A2	Homo sapiens solute carrier family 11 member 2 (SLC11A2),transcript variant 4, mRNA.
**596**	BCL2	Homo sapiens BCL2 apoptosis regulator (BCL2), transcript variantalpha, mRNA.
**6648**	SOD2	Homo sapiens superoxide dismutase 2 (SOD2), transcript variant 1,mRNA; nuclear gene for mitochondrial product.
**2936**	GSR	Homo sapiens glutathione-disulfide reductase (GSR), transcriptvariant 1, mRNA; nuclear gene for mitochondrial product.

**3589**	IL11	Homo sapiens interleukin 11 (IL11), transcript variant 1, mRNA.
**476**	ATP1A1	Homo sapiens ATPase Na+/K+ transporting subunit alpha 1 (ATP1A1),transcript variant 1, mRNA.
**1544**	CYP1A2	Homo sapiens cytochrome P450 family 1 subfamily A member 2(CYP1A2), mRNA.
**3480**	IGF1R	Homo sapiens insulin like growth factor 1 receptor (IGF1R),transcript variant 1, mRNA.
**5534**	PPP3R1	Homo sapiens protein phosphatase 3 regulatory subunit B, alpha(PPP3R1), mRNA.
**5618**	PRLR	Homo sapiens prolactin receptor (PRLR), transcript variant 1, mRNA.
**5914**	RARA	Homo sapiens retinoic acid receptor alpha (RARA), transcriptvariant 1, mRNA.
**5916**	RARG	Homo sapiens retinoic acid receptor gamma (RARG), transcriptvariant 1, mRNA.
**6165**	RPL35A	Homo sapiens ribosomal protein L35a (RPL35A), transcript variant 2,mRNA.
**490**	ATP2B1	Homo sapiens ATPase plasma membrane Ca2+ transporting 1 (ATP2B1),transcript variant 1, mRNA.
**408263**	FNDC9	Homo sapiens fibronectin type III domain containing 9 (FNDC9),mRNA.
**28511**	NKIRAS2	Homo sapiens NFKB inhibitor interacting Ras like 2 (NKIRAS2),transcript variant 1, mRNA.
**4047**	LSS	Homo sapiens lanosterol synthase (LSS), transcript variant 2, mRNA.
**339665**	SLC35E4	Homo sapiens solute carrier family 35 member E4 (SLC35E4),transcript variant 1, mRNA.
**10160**	FARP1	Homo sapiens FERM, ARH/RhoGEF and pleckstrin domain protein 1(FARP1), transcript variant 2, mRNA.
**57509**	MTUS1	Homo sapiens microtubule associated scaffold protein 1 (MTUS1),transcript variant 1, mRNA.
**55862**	ECHDC1	Homo sapiens ethylmalonyl-CoA decarboxylase 1 (ECHDC1), transcriptvariant 1, mRNA.
**517**	ATP5MC2	Homo sapiens ATP synthase membrane subunit c locus 2 (ATP5MC2),transcript variant 1, mRNA; nuclear gene for mitochondrial product.
**150368**	PHETA2	Homo sapiens PH domain containing endocytic trafficking adaptor 2(PHETA2), mRNA.
**51125**	GOLGA7	Homo sapiens golgin A7 (GOLGA7), transcript variant 2, mRNA.
**9659**	PDE4DIP	Homo sapiens phosphodiesterase 4D interacting protein (PDE4DIP),transcript variant 4, mRNA.
**65267**	WNK3	Homo sapiens WNK lysine deficient protein kinase 3 (WNK3),transcript variant 2, mRNA.
**4087**	SMAD2	Homo sapiens SMAD family member 2 (SMAD2), transcript variant 2,mRNA.
**29780**	PARVB	Homo sapiens parvin beta (PARVB), transcript variant 1, mRNA.
**8444**	DYRK3	Homo sapiens dual specificity tyrosine phosphorylation regulatedkinase 3 (DYRK3), transcript variant 2, mRNA.
**445815**	PALM2AKAP2	Homo sapiens PALM2 and AKAP2 fusion (PALM2AKAP2), transcriptvariant 5, mRNA.
**4345**	CD200	Homo sapiens CD200 molecule (CD200), transcript variant 2, mRNA.
**342132**	ZNF774	Homo sapiens zinc finger protein 774 (ZNF774), mRNA.
**389421**	LIN28B	Homo sapiens lin-28 homolog B (LIN28B), mRNA.
**219527**	LRRC55	Homo sapiens leucine rich repeat containing 55 (LRRC55), mRNA.
**6720**	SREBF1	Homo sapiens sterol regulatory element binding transcription factor1 (SREBF1), transcript variant 1, mRNA.
**205**	AK4	Homo sapiens adenylate kinase 4 (AK4), transcript variant 1, mRNA;nuclear gene for mitochondrial product.
**345557**	PLCXD3	Homo sapiens phosphatidylinositol specific phospholipase C X domaincontaining 3 (PLCXD3), mRNA.
**7341**	SUMO1	Homo sapiens small ubiquitin like modifier 1 (SUMO1), transcriptvariant 2, mRNA.
**6613**	SUMO2	Homo sapiens small ubiquitin like modifier 2 (SUMO2), transcriptvariant 2, mRNA.
**9807**	IP6K1	Homo sapiens inositol hexakisphosphate kinase 1 (IP6K1), transcriptvariant 2, mRNA.
**55914**	ERBIN	Homo sapiens erbb2 interacting protein (ERBIN), transcript variant7, mRNA.
**55339**	WDR33	Homo sapiens WD repeat domain 33 (WDR33), transcript variant 2,mRNA.
**55114**	ARHGAP17	Homo sapiens Rho GTPase activating protein 17 (ARHGAP17),transcript variant 1, mRNA.
**79712**	GTDC1	Homo sapiens glycosyltransferase like domain containing 1 (GTDC1),transcript variant 1, mRNA.
**9527**	GOSR1	Homo sapiens golgi SNAP receptor complex member 1 (GOSR1),transcript variant 3, mRNA.

**4915**	NTRK2	Homo sapiens neurotrophic receptor tyrosine kinase 2 (NTRK2),transcript variant b, mRNA.
**3321**	IGSF3	Homo sapiens immunoglobulin superfamily member 3 (IGSF3),transcript variant 2, mRNA.
**54014**	BRWD1	Homo sapiens bromodomain and WD repeat domain containing 1 (BRWD1),transcript variant 3, mRNA.
**79760**	GEMIN7	Homo sapiens gem nuclear organelle associated protein 7 (GEMIN7),transcript variant 2, mRNA.
**80036**	TRPM3	Homo sapiens transient receptor potential cation channel subfamilyM member 3 (TRPM3), transcript variant 8, mRNA.
**51114**	ZDHHC9	Homo sapiens zinc finger DHHC-type palmitoyltransferase 9 (ZDHHC9),transcript variant 2, mRNA.
**493869**	GPX8	Homo sapiens glutathione peroxidase 8 (putative) (GPX8), transcriptvariant 1, mRNA.
**55740**	ENAH	Homo sapiens ENAH actin regulator (ENAH), transcript variant 1,mRNA.
**169200**	TMEM64	Homo sapiens transmembrane protein 64 (TMEM64), transcript variant1, mRNA.
**9818**	NUP58	Homo sapiens nucleoporin 58 (NUP58), transcript variant 2, mRNA.
**96459**	FNIP1	Homo sapiens folliculin interacting protein 1 (FNIP1), transcriptvariant 2, mRNA.
**401409**	RAB19	Homo sapiens RAB19, member RAS oncogene family (RAB19), mRNA.
**152503**	SH3D19	Homo sapiens SH3 domain containing 19 (SH3D19), transcript variant1, mRNA.
**135293**	PM20D2	Homo sapiens peptidase M20 domain containing 2 (PM20D2), mRNA.
**169981**	SPIN3	Homo sapiens spindlin family member 3 (SPIN3), transcript variant1, mRNA.
**199953**	TMEM201	Homo sapiens transmembrane protein 201 (TMEM201), transcriptvariant 2, mRNA.
**200205**	IBA57	Homo sapiens iron-sulfur cluster assembly factor IBA57 (IBA57),transcript variant 1, mRNA; nuclear gene for mitochondrial product.
**340485**	ACER2	Homo sapiens alkaline ceramidase 2 (ACER2), mRNA.
**345778**	MTX3	Homo sapiens metaxin 3 (MTX3), transcript variant 2, mRNA.
**9709**	HERPUD1	Homo sapiens homocysteine inducible ER protein with ubiquitin likedomain 1 (HERPUD1), transcript variant 2, mRNA.
**122830**	NAA30	Homo sapiens N-alpha-acetyltransferase 30, NatC catalytic subunit(NAA30), mRNA.
**134218**	DNAJC21	Homo sapiens DnaJ heat shock protein family (Hsp40) member C21(DNAJC21), transcript variant 2, mRNA.
**9570**	GOSR2	Homo sapiens golgi SNAP receptor complex member 2 (GOSR2),transcript variant C, mRNA.
**339324**	ZNF260	Homo sapiens zinc finger protein 260 (ZNF260), transcript variant1, mRNA.
**27185**	DISC1	Homo sapiens DISC1 scaffold protein (DISC1), transcript variant Lv,mRNA.
**338879**	RNASE10	Homo sapiens ribonuclease A family member 10 (inactive) (RNASE10),transcript variant 2, mRNA.
**391059**	FRRS1	Homo sapiens ferric chelate reductase 1 (FRRS1), transcript variant1, mRNA.
**860**	RUNX2	Homo sapiens RUNX family transcription factor 2 (RUNX2), transcriptvariant 2, mRNA.
**117584**	RFFL	Homo sapiens ring finger and FYVE like domain containing E3ubiquitin protein ligase (RFFL), transcript variant 2, mRNA.
**3320**	HSP90AA1	Homo sapiens heat shock protein 90 alpha family class A member 1(HSP90AA1), transcript variant 1, mRNA.
**80207**	OPA3	Homo sapiens outer mitochondrial membrane lipid metabolismregulator OPA3 (OPA3), transcript variant 1, mRNA.
**26135**	SERBP1	Homo sapiens SERPINE1 mRNA binding protein 1 (SERBP1), transcriptvariant 1, mRNA.
**821**	CANX	Homo sapiens calnexin (CANX), transcript variant 2, mRNA.
**80381**	CD276	Homo sapiens CD276 molecule (CD276), transcript variant 1, mRNA.
**605**	BCL7A	Homo sapiens BAF chromatin remodeling complex subunit BCL7A(BCL7A), transcript variant 2, mRNA.
**196527**	ANO6	Homo sapiens anoctamin 6 (ANO6), transcript variant 1, mRNA.
**7422**	VEGFA	Homo sapiens vascular endothelial growth factor A (VEGFA),transcript variant 1, mRNA.
**10658**	CELF1	Homo sapiens CUGBP Elav-like family member 1 (CELF1), transcriptvariant 3, mRNA.
**6229**	RPS24	Homo sapiens ribosomal protein S24 (RPS24), transcript variant c,mRNA.

**222553**	SLC35F1	Homo sapiens solute carrier family 35 member F1 (SLC35F1), mRNA.
**283219**	KCTD21	Homo sapiens potassium channel tetramerization domain containing 21(KCTD21), mRNA.
**84068**	SLC10A7	Homo sapiens solute carrier family 10 member 7 (SLC10A7),transcript variant 2, mRNA.
**389432**	SAMD5	Homo sapiens sterile alpha motif domain containing 5 (SAMD5), mRNA.
**6693**	SPN	Homo sapiens sialophorin (SPN), transcript variant 1, mRNA.
**23543**	RBFOX2	Homo sapiens RNA binding fox-1 homolog 2 (RBFOX2), transcriptvariant 1, mRNA.
**57222**	ERGIC1	Homo sapiens endoplasmic reticulum-golgi intermediate compartment 1(ERGIC1), mRNA.
**79807**	GSTCD	Homo sapiens glutathione S-transferase C-terminal domain containing(GSTCD), transcript variant 1, mRNA.
**91746**	YTHDC1	Homo sapiens YTH domain containing 1 (YTHDC1), transcript variant1, mRNA.
**7756**	ZNF207	Homo sapiens zinc finger protein 207 (ZNF207), transcript variant2, mRNA.
**26100**	WIPI2	Homo sapiens WD repeat domain, phosphoinositide interacting 2(WIPI2), transcript variant 3, mRNA.
**23597**	ACOT9	Homo sapiens acyl-CoA thioesterase 9 (ACOT9), transcript variant 2,mRNA; nuclear gene for mitochondrial product.
**7311**	UBA52	Homo sapiens ubiquitin A-52 residue ribosomal protein fusionproduct 1 (UBA52), transcript variant 1, mRNA.
**6241**	RRM2	Homo sapiens ribonucleotide reductase regulatory subunit M2 (RRM2),transcript variant 2, mRNA.
**554313**	H4C15	Homo sapiens H4 clustered histone 15 (H4C15), transcript variant 1,mRNA.
**64093**	SMOC1	Homo sapiens SPARC related modular calcium binding 1 (SMOC1),transcript variant 1, mRNA.
**64855**	NIBAN2	Homo sapiens niban apoptosis regulator 2 (NIBAN2), transcriptvariant 2, mRNA.
**116987**	AGAP1	Homo sapiens ArfGAP with GTPase domain, ankyrin repeat and PHdomain 1 (AGAP1), transcript variant 1, mRNA.
**9453**	GGPS1	Homo sapiens geranylgeranyl diphosphate synthase 1 (GGPS1),transcript variant 2, mRNA.
**9097**	USP14	Homo sapiens ubiquitin specific peptidase 14 (USP14), transcriptvariant 2, mRNA.
**8663**	EIF3C	Homo sapiens eukaryotic translation initiation factor 3 subunit C(EIF3C), transcript variant 2, mRNA.
**56882**	CDC42SE1	Homo sapiens CDC42 small effector 1 (CDC42SE1), transcript variant1, mRNA.
**6340**	SCNN1G	Homo sapiens sodium channel epithelial 1 subunit gamma (SCNN1G),mRNA.
**9870**	AREL1	Homo sapiens apoptosis resistant E3 ubiquitin protein ligase 1(AREL1), mRNA.
**2274**	FHL2	Homo sapiens four and a half LIM domains 2 (FHL2), transcriptvariant 5, mRNA.
**256356**	GK5	Homo sapiens glycerol kinase 5 (GK5), transcript variant 1, mRNA.
**83999**	KREMEN1	Homo sapiens kringle containing transmembrane protein 1 (KREMEN1),transcript variant 3, mRNA.
**998**	CDC42	Homo sapiens cell division cycle 42 (CDC42), transcript variant 3,mRNA.
**644815**	FAM83G	Homo sapiens family with sequence similarity 83 member G (FAM83G),mRNA.
**23089**	PEG10	Homo sapiens paternally expressed 10 (PEG10), transcript variant 1,mRNA.
**3955**	LFNG	Homo sapiens LFNG O-fucosylpeptide3-beta-N-acetylglucosaminyltransferase (LFNG), transcript variant
**64282**	TENT4B	Homo sapiens terminal nucleotidyltransferase 4B (TENT4B),transcript variant 1, mRNA.
**5789**	PTPRD	Homo sapiens protein tyrosine phosphatase receptor type D (PTPRD),transcript variant 5, mRNA.
**9581**	PREPL	Homo sapiens prolyl endopeptidase like (PREPL), transcript variant4, mRNA.
**5660**	PSAP	Homo sapiens prosaposin (PSAP), transcript variant 2, mRNA.
**23589**	CARHSP1	Homo sapiens calcium regulated heat stable protein 1 (CARHSP1),transcript variant 2, mRNA.
**7170**	TPM3	Homo sapiens tropomyosin 3 (TPM3), transcript variant Tpm3.2, mRNA.
**65979**	PHACTR4	Homo sapiens phosphatase and actin regulator 4 (PHACTR4),transcript variant 1, mRNA.
**29128**	UHRF1	Homo sapiens ubiquitin like with PHD and ring finger domains 1(UHRF1), transcript variant 1, mRNA.
**55861**	DBNDD2	Homo sapiens dysbindin domain containing 2 (DBNDD2), transcriptvariant 1, mRNA.
**8555**	CDC14B	Homo sapiens cell division cycle 14B (CDC14B), transcript variant3, mRNA.

**9612**	NCOR2	Homo sapiens nuclear receptor corepressor 2 (NCOR2), transcriptvariant 2, mRNA.
**137886**	UBXN2B	Homo sapiens UBX domain protein 2B (UBXN2B), transcript variant 1,mRNA.
**55665**	URGCP	Homo sapiens upregulator of cell proliferation (URGCP), transcriptvariant 3, mRNA.
**22807**	IKZF2	Homo sapiens IKAROS family zinc finger 2 (IKZF2), transcriptvariant 2, mRNA.
**22862**	FNDC3A	Homo sapiens fibronectin type III domain containing 3A (FNDC3A),transcript variant 1, mRNA.
**1729**	DIAPH1	Homo sapiens diaphanous related formin 1 (DIAPH1), transcriptvariant 2, mRNA.
**10052**	GJC1	Homo sapiens gap junction protein gamma 1 (GJC1), transcriptvariant 2, mRNA.
**6672**	SP100	Homo sapiens SP100 nuclear antigen (SP100), transcript variant 1,mRNA.
**23361**	ZNF629	Homo sapiens zinc finger protein 629 (ZNF629), transcript variant1, mRNA.
**23025**	UNC13A	Homo sapiens unc-13 homolog A (UNC13A), transcript variant 1, mRNA.
**27324**	TOX3	Homo sapiens TOX high mobility group box family member 3 (TOX3),transcript variant 1, mRNA.
**79068**	FTO	Homo sapiens FTO alpha-ketoglutarate dependent dioxygenase (FTO),transcript variant 3, mRNA.
**10838**	ZNF275	Homo sapiens zinc finger protein 275 (ZNF275), transcript variant2, mRNA.
**84747**	UNC119B	Homo sapiens unc-119 lipid binding chaperone B (UNC119B), mRNA.
**55216**	NKAPD1	Homo sapiens NKAP domain containing 1 (NKAPD1), transcript variant2, mRNA.
**6925**	TCF4	Homo sapiens transcription factor 4 (TCF4), transcript variant 1,mRNA.
**84923**	FAM104A	Homo sapiens family with sequence similarity 104 member A(FAM104A), transcript variant 1, mRNA.
**57604**	TRMT9B	Homo sapiens tRNA methyltransferase 9B (putative) (TRMT9B),transcript variant 2, mRNA.
**143244**	EIF5AL1	Homo sapiens eukaryotic translation initiation factor 5A like 1(EIF5AL1), mRNA.
**147660**	ZNF578	Homo sapiens zinc finger protein 578 (ZNF578), transcript variant1, mRNA.
**58**	ACTA1	Homo sapiens actin alpha 1, skeletal muscle (ACTA1), mRNA.
**201931**	TMEM192	Homo sapiens transmembrane protein 192 (TMEM192), mRNA.
**91768**	CABLES1	Homo sapiens Cdk5 and Abl enzyme substrate 1 (CABLES1), transcriptvariant 2, mRNA.
**55041**	PLEKHB2	Homo sapiens pleckstrin homology domain containing B2 (PLEKHB2),transcript variant 3, mRNA.
**10613**	ERLIN1	Homo sapiens ER lipid raft associated 1 (ERLIN1), transcriptvariant 2, mRNA.
**80020**	FOXRED2	Homo sapiens FAD dependent oxidoreductase domain containing 2(FOXRED2), transcript variant 2, mRNA.
**80143**	SIKE1	Homo sapiens suppressor of IKBKE 1 (SIKE1), transcript variant 1,mRNA.
**54852**	PAQR5	Homo sapiens progestin and adipoQ receptor family member 5 (PAQR5),transcript variant 1, mRNA.
**55186**	SLC25A36	Homo sapiens solute carrier family 25 member 36 (SLC25A36),transcript variant 1, mRNA.
**205147**	AMER3	Homo sapiens APC membrane recruitment protein 3 (AMER3), transcriptvariant 2, mRNA.
**4204**	MECP2	Homo sapiens methyl-CpG binding protein 2 (MECP2), transcriptvariant 2, mRNA.
**1075**	CTSC	Homo sapiens cathepsin C (CTSC), transcript variant 3, mRNA.
**26099**	SZRD1	Homo sapiens SUZ RNA binding domain containing 1 (SZRD1),transcript variant 1, mRNA.
**5324**	PLAG1	Homo sapiens PLAG1 zinc finger (PLAG1), transcript variant 2, mRNA.
**80232**	WDR26	Homo sapiens WD repeat domain 26 (WDR26), transcript variant 2,mRNA.
**167691**	LCA5	Homo sapiens lebercilin LCA5 (LCA5), transcript variant 2, mRNA.
**84614**	ZBTB37	Homo sapiens zinc finger and BTB domain containing 37 (ZBTB37),transcript variant 1, mRNA.
**4267**	CD99	Homo sapiens CD99 molecule (Xg blood group) (CD99), transcriptvariant 2, mRNA.
**10295**	BCKDK	Homo sapiens branched chain keto acid dehydrogenase kinase (BCKDK),transcript variant 2, mRNA; nuclear gene for mitochondrial product.
**132**	ADK	Homo sapiens adenosine kinase (ADK), transcript variant 1, mRNA.
**60386**	SLC25A19	Homo sapiens solute carrier family 25 member 19 (SLC25A19),transcript variant 1, mRNA; nuclear gene for mitochondrial product.

**80228**	ORAI2	Homo sapiens ORAI calcium release-activated calcium modulator 2(ORAI2), transcript variant 1, mRNA.
**27113**	BBC3	Homo sapiens BCL2 binding component 3 (BBC3), transcript variant 1,mRNA.
**23468**	CBX5	Homo sapiens chromobox 5 (CBX5), transcript variant 2, mRNA.
**7637**	ZNF84	Homo sapiens zinc finger protein 84 (ZNF84), transcript variant 2,mRNA.
**1408**	CRY2	Homo sapiens cryptochrome circadian regulator 2 (CRY2), transcriptvariant 2, mRNA.
**81848**	SPRY4	Homo sapiens sprouty RTK signaling antagonist 4 (SPRY4), transcriptvariant 2, mRNA.
**3977**	LIFR	Homo sapiens LIF receptor subunit alpha (LIFR), transcript variant1, mRNA.
**6007**	RHD	Homo sapiens Rh blood group D antigen (RHD), transcript variant 2,mRNA.
**6935**	ZEB1	Homo sapiens zinc finger E-box binding homeobox 1 (ZEB1),transcript variant 1, mRNA.
**7037**	TFRC	Homo sapiens transferrin receptor (TFRC), transcript variant 2,mRNA.
**1742**	DLG4	Homo sapiens discs large MAGUK scaffold protein 4 (DLG4),transcript variant 2, mRNA.
**9583**	ENTPD4	Homo sapiens ectonucleoside triphosphate diphosphohydrolase 4(ENTPD4), transcript variant 2, mRNA.
**10087**	CERT1	Homo sapiens ceramide transporter 1 (CERT1), transcript variant 3,mRNA.
**10413**	YAP1	Homo sapiens Yes1 associated transcriptional regulator (YAP1),transcript variant 1, mRNA.
**1977**	EIF4E	Homo sapiens eukaryotic translation initiation factor 4E (EIF4E),transcript variant 3, mRNA.
**26034**	IPCEF1	Homo sapiens interaction protein for cytohesin exchange factors 1(IPCEF1), transcript variant 1, mRNA.
**23382**	AHCYL2	Homo sapiens adenosylhomocysteinase like 2 (AHCYL2), transcriptvariant 2, mRNA.
**79071**	ELOVL6	Homo sapiens ELOVL fatty acid elongase 6 (ELOVL6), transcriptvariant 2, mRNA.
**8634**	RTCA	Homo sapiens RNA 3'-terminal phosphate cyclase (RTCA), transcriptvariant 1, mRNA.
**7046**	TGFBR1	Homo sapiens transforming growth factor beta receptor 1 (TGFBR1),transcript variant 2, mRNA.
**25946**	ZNF385A	Homo sapiens zinc finger protein 385A (ZNF385A), transcript variant1, mRNA.
**4208**	MEF2C	Homo sapiens myocyte enhancer factor 2C (MEF2C), transcript variant2, mRNA.
**84240**	ZCCHC9	Homo sapiens zinc finger CCHC-type containing 9 (ZCCHC9),transcript variant 2, mRNA.
**27102**	EIF2AK1	Homo sapiens eukaryotic translation initiation factor 2 alphakinase 1 (EIF2AK1), transcript variant 2, mRNA.
**4134**	MAP4	Homo sapiens microtubule associated protein 4 (MAP4), transcriptvariant 4, mRNA.
**54619**	CCNJ	Homo sapiens cyclin J (CCNJ), transcript variant 1, mRNA.
**7410**	VAV2	Homo sapiens vav guanine nucleotide exchange factor 2 (VAV2),transcript variant 1, mRNA.
**55573**	CDV3	Homo sapiens CDV3 homolog (CDV3), transcript variant 1, mRNA.
**91392**	ZNF502	Homo sapiens zinc finger protein 502 (ZNF502), transcript variant2, mRNA.
**9653**	HS2ST1	Homo sapiens heparan sulfate 2-O-sulfotransferase 1 (HS2ST1),transcript variant 2, mRNA.
**159371**	SLC35G1	Homo sapiens solute carrier family 35 member G1 (SLC35G1),transcript variant 1, mRNA.
**5090**	PBX3	Homo sapiens PBX homeobox 3 (PBX3), transcript variant 2, mRNA.
**100129792**	CCDC152	Homo sapiens coiled-coil domain containing 152 (CCDC152), mRNA.
**4238**	MFAP3	Homo sapiens microfibril associated protein 3 (MFAP3), transcriptvariant 2, mRNA.
**6883**	TAF12	Homo sapiens TATA-box binding protein associated factor 12 (TAF12),transcript variant 1, mRNA.
**3298**	HSF2	Homo sapiens heat shock transcription factor 2 (HSF2), transcriptvariant 2, mRNA.
**11082**	ESM1	Homo sapiens endothelial cell specific molecule 1 (ESM1),transcript variant 2, mRNA.
**64225**	ATL2	Homo sapiens atlastin GTPase 2 (ATL2), transcript variant 2, mRNA.
**7534**	YWHAZ	Homo sapiens tyrosine 3-monooxygenase/tryptophan 5-monooxygenaseactivation protein zeta (YWHAZ), transcript variant 3, mRNA.
**9825**	SPATA2	Homo sapiens spermatogenesis associated 2 (SPATA2), transcriptvariant 2, mRNA.
**6857**	SYT1	Homo sapiens synaptotagmin 1 (SYT1), transcript variant 2, mRNA.

**23670**	CEMIP2	Homo sapiens cell migration inducing hyaluronidase 2 (CEMIP2),transcript variant 2, mRNA.
**27**	ABL2	Homo sapiens ABL proto-oncogene 2, non-receptor tyrosine kinase(ABL2), transcript variant d, mRNA.
**896**	CCND3	Homo sapiens cyclin D3 (CCND3), transcript variant 1, mRNA.
**5358**	PLS3	Homo sapiens plastin 3 (PLS3), transcript variant 2, mRNA.
**55719**	SLF2	Homo sapiens SMC5-SMC6 complex localization factor 2 (SLF2),transcript variant 2, mRNA.
**1388**	ATF6B	Homo sapiens activating transcription factor 6 beta (ATF6B),transcript variant 2, mRNA.
**30000**	TNPO2	Homo sapiens transportin 2 (TNPO2), transcript variant 3, mRNA.
**9802**	DAZAP2	Homo sapiens DAZ associated protein 2 (DAZAP2), transcript variant2, mRNA.
**441151**	TMEM151B	Homo sapiens transmembrane protein 151B (TMEM151B), mRNA.
**342371**	ATXN1L	Homo sapiens ataxin 1 like (ATXN1L), mRNA.
**54715**	RBFOX1	Homo sapiens RNA binding fox-1 homolog 1 (RBFOX1), transcriptvariant 5, mRNA.
**23439**	ATP1B4	Homo sapiens ATPase Na+/K+ transporting family member beta 4(ATP1B4), transcript variant 1, mRNA.
**9214**	FCMR	Homo sapiens Fc mu receptor (FCMR), transcript variant 3, mRNA.
**56681**	SAR1A	Homo sapiens secretion associated Ras related GTPase 1A (SAR1A),transcript variant 1, mRNA.
**79094**	CHAC1	Homo sapiens ChaC glutathione specificgamma-glutamylcyclotransferase 1 (CHAC1), transcript variant 2,
**627**	BDNF	Homo sapiens brain derived neurotrophic factor (BDNF), transcriptvariant 7, mRNA.
**2113**	ETS1	Homo sapiens ETS proto-oncogene 1, transcription factor (ETS1),transcript variant 1, mRNA.
**10982**	MAPRE2	Homo sapiens microtubule associated protein RP/EB family member 2(MAPRE2), transcript variant 2, mRNA.
**148867**	SLC30A7	Homo sapiens solute carrier family 30 member 7 (SLC30A7),transcript variant 2, mRNA.
**729438**	CASTOR2	Homo sapiens cytosolic arginine sensor for mTORC1 subunit 2(CASTOR2), mRNA.
**1375**	CPT1B	Homo sapiens carnitine palmitoyltransferase 1B (CPT1B), transcriptvariant 5, mRNA.
**7171**	TPM4	Homo sapiens tropomyosin 4 (TPM4), transcript variant 2, mRNA.
**1021**	CDK6	Homo sapiens cyclin dependent kinase 6 (CDK6), transcript variant2, mRNA.
**83856**	FSD1L	Homo sapiens fibronectin type III and SPRY domain containing 1 like(FSD1L), transcript variant 3, mRNA.
**22834**	ZNF652	Homo sapiens zinc finger protein 652 (ZNF652), transcript variant1, mRNA.
**9869**	SETDB1	Homo sapiens SET domain bifurcated histone lysine methyltransferase1 (SETDB1), transcript variant 1, mRNA.
**9805**	SCRN1	Homo sapiens secernin 1 (SCRN1), transcript variant 1, mRNA.
**2289**	FKBP5	Homo sapiens FKBP prolyl isomerase 5 (FKBP5), transcript variant 2,mRNA.
**51005**	AMDHD2	Homo sapiens amidohydrolase domain containing 2 (AMDHD2),transcript variant 2, mRNA.
**23181**	DIP2A	Homo sapiens disco interacting protein 2 homolog A (DIP2A),transcript variant 6, mRNA.
**55117**	SLC6A15	Homo sapiens solute carrier family 6 member 15 (SLC6A15),transcript variant 3, mRNA.
**9904**	RBM19	Homo sapiens RNA binding motif protein 19 (RBM19), transcriptvariant 3, mRNA.
**60592**	SCOC	Homo sapiens short coiled-coil protein (SCOC), transcript variant6, mRNA.
**64779**	MTHFSD	Homo sapiens methenyltetrahydrofolate synthetase domain containing(MTHFSD), transcript variant 1, mRNA.
**389206**	BEND4	Homo sapiens BEN domain containing 4 (BEND4), transcript variant 2,mRNA.
**201266**	SLC39A11	Homo sapiens solute carrier family 39 member 11 (SLC39A11),transcript variant 1, mRNA.
**8867**	SYNJ1	Homo sapiens synaptojanin 1 (SYNJ1), transcript variant 3, mRNA.
**25886**	POC1A	Homo sapiens POC1 centriolar protein A (POC1A), transcript variant2, mRNA.
**56245**	C21orf62	Homo sapiens chromosome 21 open reading frame 62 (C21orf62),transcript variant 1, mRNA.
**494115**	RBMXL1	Homo sapiens RBMX like 1 (RBMXL1), transcript variant 1, mRNA.

**9468**	PCYT1B	Homo sapiens phosphate cytidylyltransferase 1B, choline (PCYT1B),transcript variant 2, mRNA.
**51200**	CPA4	Homo sapiens carboxypeptidase A4 (CPA4), transcript variant 2,mRNA.
**155061**	ZNF746	Homo sapiens zinc finger protein 746 (ZNF746), transcript variant1, mRNA.
**80344**	DCAF11	Homo sapiens DDB1 and CUL4 associated factor 11 (DCAF11),transcript variant 3, mRNA.
**26137**	ZBTB20	Homo sapiens zinc finger and BTB domain containing 20 (ZBTB20),transcript variant 1, mRNA.
**651746**	ANKRD33B	Homo sapiens ankyrin repeat domain 33B (ANKRD33B), mRNA.
**27161**	AGO2	Homo sapiens argonaute RISC catalytic component 2 (AGO2),transcript variant 2, mRNA.
**149466**	C1orf210	Homo sapiens chromosome 1 open reading frame 210 (C1orf210),transcript variant 2, mRNA.
**10795**	ZNF268	Homo sapiens zinc finger protein 268 (ZNF268), transcript variant2, mRNA.
**2067**	ERCC1	Homo sapiens ERCC excision repair 1, endonuclease non-catalyticsubunit (ERCC1), transcript variant 3, mRNA.
**4720**	NDUFS2	Homo sapiens NADH:ubiquinone oxidoreductase core subunit S2(NDUFS2), transcript variant 2, mRNA; nuclear gene for
**153241**	CEP120	Homo sapiens centrosomal protein 120 (CEP120), transcript variant2, mRNA.
**51744**	CD244	Homo sapiens CD244 molecule (CD244), transcript variant 2, mRNA.
**331**	XIAP	Homo sapiens X-linked inhibitor of apoptosis (XIAP), transcriptvariant 1, mRNA.
**4026**	LPP	Homo sapiens LIM domain containing preferred translocation partnerin lipoma (LPP), transcript variant 2, mRNA.
**50999**	TMED5	Homo sapiens transmembrane p24 trafficking protein 5 (TMED5),transcript variant 2, mRNA.
**1906**	EDN1	Homo sapiens endothelin 1 (EDN1), transcript variant 2, mRNA.
**10099**	TSPAN3	Homo sapiens tetraspanin 3 (TSPAN3), transcript variant 3, mRNA.
**55787**	TXLNG	Homo sapiens taxilin gamma (TXLNG), transcript variant 2, mRNA.
**23380**	SRGAP2	Homo sapiens SLIT-ROBO Rho GTPase activating protein 2 (SRGAP2),transcript variant 3, mRNA.
**60468**	BACH2	Homo sapiens BTB domain and CNC homolog 2 (BACH2), transcriptvariant 2, mRNA.
**79036**	KXD1	Homo sapiens KxDL motif containing 1 (KXD1), transcript variant 1,mRNA.
**55762**	ZNF701	Homo sapiens zinc finger protein 701 (ZNF701), transcript variant1, mRNA.
**23163**	GGA3	Homo sapiens golgi associated, gamma adaptin ear containing, ARFbinding protein 3 (GGA3), transcript variant 1, mRNA.
**26051**	PPP1R16B	Homo sapiens protein phosphatase 1 regulatory subunit 16B(PPP1R16B), transcript variant 2, mRNA.
**27343**	POLL	Homo sapiens DNA polymerase lambda (POLL), transcript variant 1,mRNA.
**10046**	MAMLD1	Homo sapiens mastermind like domain containing 1 (MAMLD1),transcript variant 1, mRNA.
**5520**	PPP2R2A	Homo sapiens protein phosphatase 2 regulatory subunit Balpha(PPP2R2A), transcript variant 2, mRNA.
**6809**	STX3	Homo sapiens syntaxin 3 (STX3), transcript variant 2, mRNA.
**586**	BCAT1	Homo sapiens branched chain amino acid transaminase 1 (BCAT1),transcript variant 2, mRNA.
**7029**	TFDP2	Homo sapiens transcription factor Dp-2 (TFDP2), transcript variant1, mRNA.
**501**	ALDH7A1	Homo sapiens aldehyde dehydrogenase 7 family member A1 (ALDH7A1),transcript variant 1, mRNA.
**2992**	GYG1	Homo sapiens glycogenin 1 (GYG1), transcript variant 2, mRNA.
**23245**	ASTN2	Homo sapiens astrotactin 2 (ASTN2), transcript variant 5, mRNA.
**84961**	FBXL20	Homo sapiens F-box and leucine rich repeat protein 20 (FBXL20),transcript variant 2, mRNA.
**571**	BACH1	Homo sapiens BTB domain and CNC homolog 1 (BACH1), transcriptvariant 2, mRNA.
**3551**	IKBKB	Homo sapiens inhibitor of nuclear factor kappa B kinase subunitbeta (IKBKB), transcript variant 2, mRNA.
**5000**	ORC4	Homo sapiens origin recognition complex subunit 4 (ORC4),transcript variant 4, mRNA.
**598**	BCL2L1	Homo sapiens BCL2 like 1 (BCL2L1), transcript variant 2, mRNA.
**23534**	TNPO3	Homo sapiens transportin 3 (TNPO3), transcript variant 2, mRNA.
**55654**	TMEM127	Homo sapiens transmembrane protein 127 (TMEM127), transcriptvariant 2, mRNA.

**5524**	PTPA	Homo sapiens protein phosphatase 2 phosphatase activator (PTPA),transcript variant 6, mRNA.
**6506**	SLC1A2	Homo sapiens solute carrier family 1 member 2 (SLC1A2), transcriptvariant 2, mRNA.
**80829**	ZFP91	Homo sapiens ZFP91 zinc finger protein, atypical E3 ubiquitinligase (ZFP91), transcript variant 2, mRNA.
**432**	ASGR1	Homo sapiens asialoglycoprotein receptor 1 (ASGR1), transcriptvariant 2, mRNA.
**4170**	MCL1	Homo sapiens MCL1 apoptosis regulator, BCL2 family member (MCL1),transcript variant 3, mRNA.
**9936**	CD302	Homo sapiens CD302 molecule (CD302), transcript variant 2, mRNA.
**8453**	CUL2	Homo sapiens cullin 2 (CUL2), transcript variant 4, mRNA.
**5451**	POU2F1	Homo sapiens POU class 2 homeobox 1 (POU2F1), transcript variant 2,mRNA.
**29974**	A1CF	Homo sapiens APOBEC1 complementation factor (A1CF), transcriptvariant 4, mRNA.
**22887**	FOXJ3	Homo sapiens forkhead box J3 (FOXJ3), transcript variant 2, mRNA.
**9296**	ATP6V1F	Homo sapiens ATPase H+ transporting V1 subunit F (ATP6V1F),transcript variant 2, mRNA.
**80031**	SEMA6D	Homo sapiens semaphorin 6D (SEMA6D), transcript variant 7, mRNA.
**84516**	DCTN5	Homo sapiens dynactin subunit 5 (DCTN5), transcript variant 2,mRNA.
**83607**	AMMECR1L	Homo sapiens AMMECR1 like (AMMECR1L), transcript variant 2, mRNA.
**163590**	TOR1AIP2	Homo sapiens torsin 1A interacting protein 2 (TOR1AIP2), transcriptvariant 3, mRNA.
**8729**	GBF1	Homo sapiens golgi brefeldin A resistant guanine nucleotideexchange factor 1 (GBF1), transcript variant 2, mRNA.
**56919**	DHX33	Homo sapiens DEAH-box helicase 33 (DHX33), transcript variant 2,mRNA.
**79017**	GGCT	Homo sapiens gamma-glutamylcyclotransferase (GGCT), transcriptvariant 2, mRNA.
**599**	BCL2L2	Homo sapiens BCL2 like 2 (BCL2L2), transcript variant 2, mRNA.
**5238**	PGM3	Homo sapiens phosphoglucomutase 3 (PGM3), transcript variant 1,mRNA.
**71**	ACTG1	Homo sapiens actin gamma 1 (ACTG1), transcript variant 1, mRNA.
**4712**	NDUFB6	Homo sapiens NADH:ubiquinone oxidoreductase subunit B6 (NDUFB6),transcript variant 3, mRNA.
**10771**	ZMYND11	Homo sapiens zinc finger MYND-type containing 11 (ZMYND11),transcript variant 4, mRNA.
**7327**	UBE2G2	Homo sapiens ubiquitin conjugating enzyme E2 G2 (UBE2G2),transcript variant 3, mRNA.
**4084**	MXD1	Homo sapiens MAX dimerization protein 1 (MXD1), transcript variant2, mRNA.
**1523**	CUX1	Homo sapiens cut like homeobox 1 (CUX1), transcript variant 4,mRNA.
**57715**	SEMA4G	Homo sapiens semaphorin 4G (SEMA4G), transcript variant 2, mRNA.
**4194**	MDM4	Homo sapiens MDM4 regulator of p53 (MDM4), transcript variant 2,mRNA.
**6728**	SRP19	Homo sapiens signal recognition particle 19 (SRP19), transcriptvariant 2, mRNA.
**818**	CAMK2G	Homo sapiens calcium/calmodulin dependent protein kinase II gamma(CAMK2G), transcript variant 7, mRNA.
**84914**	ZNF587	Homo sapiens zinc finger protein 587 (ZNF587), transcript variant2, mRNA.
**8840**	CCN4	Homo sapiens cellular communication network factor 4 (CCN4),transcript variant 3, mRNA.
**64359**	NXN	Homo sapiens nucleoredoxin (NXN), transcript variant 2, mRNA.
**57506**	MAVS	Homo sapiens mitochondrial antiviral signaling protein (MAVS),transcript variant 3, mRNA; nuclear gene for mitochondrial product.
**51100**	SH3GLB1	Homo sapiens SH3 domain containing GRB2 like, endophilin B1(SH3GLB1), transcript variant 2, mRNA.
**10618**	TGOLN2	Homo sapiens trans-golgi network protein 2 (TGOLN2), transcriptvariant 2, A allele, mRNA.
**8854**	ALDH1A2	Homo sapiens aldehyde dehydrogenase 1 family member A2 (ALDH1A2),transcript variant 4, mRNA.
**5452**	POU2F2	Homo sapiens POU class 2 homeobox 2 (POU2F2), transcript variant 1,mRNA.
**1789**	DNMT3B	Homo sapiens DNA methyltransferase 3 beta (DNMT3B), transcriptvariant 7, mRNA.
**9689**	BZW1	Homo sapiens basic leucine zipper and W2 domains 1 (BZW1),transcript variant 1, mRNA.
**197407**	ZNF48	Homo sapiens zinc finger protein 48 (ZNF48), transcript variant 2,mRNA.

**771**	CA12	Homo sapiens carbonic anhydrase 12 (CA12), transcript variant 1,mRNA.
**905**	CCNT2	Homo sapiens cyclin T2 (CCNT2), transcript variant a, mRNA.
**5295**	PIK3R1	Homo sapiens phosphoinositide-3-kinase regulatory subunit 1(PIK3R1), transcript variant 4, mRNA.
**84285**	EIF1AD	Homo sapiens eukaryotic translation initiation factor 1A domaincontaining (EIF1AD), transcript variant 1, mRNA.
**55916**	NXT2	Homo sapiens nuclear transport factor 2 like export factor 2(NXT2), transcript variant 2, mRNA.
**10768**	AHCYL1	Homo sapiens adenosylhomocysteinase like 1 (AHCYL1), transcriptvariant 2, mRNA.
**8165**	AKAP1	Homo sapiens A-kinase anchoring protein 1 (AKAP1), transcriptvariant 2, mRNA; nuclear gene for mitochondrial product.
**208**	AKT2	Homo sapiens AKT serine/threonine kinase 2 (AKT2), transcriptvariant 2, mRNA.
**1871**	E2F3	Homo sapiens E2F transcription factor 3 (E2F3), transcript variant2, mRNA.
**51203**	NUSAP1	Homo sapiens nucleolar and spindle associated protein 1 (NUSAP1),transcript variant 4, mRNA.
**26953**	RANBP6	Homo sapiens RAN binding protein 6 (RANBP6), transcript variant 2,mRNA.
**79731**	NARS2	Homo sapiens asparaginyl-tRNA synthetase 2, mitochondrial (NARS2),transcript variant 2, mRNA; nuclear gene for mitochondrial product.
**1073**	CFL2	Homo sapiens cofilin 2 (CFL2), transcript variant 5, mRNA.
**7165**	TPD52L2	Homo sapiens TPD52 like 2 (TPD52L2), transcript variant 7, mRNA.
**6689**	SPIB	Homo sapiens Spi-B transcription factor (SPIB), transcript variant2, mRNA.
**4782**	NFIC	Homo sapiens nuclear factor I C (NFIC), transcript variant 1, mRNA.
**6667**	SP1	Homo sapiens Sp1 transcription factor (SP1), transcript variant 3,mRNA.
**10914**	PAPOLA	Homo sapiens poly(A) polymerase alpha (PAPOLA), transcript variant2, mRNA.
**9066**	SYT7	Homo sapiens synaptotagmin 7 (SYT7), transcript variant 1, mRNA.
**84952**	CGNL1	Homo sapiens cingulin like 1 (CGNL1), transcript variant 1, mRNA.
**148281**	SYT6	Homo sapiens synaptotagmin 6 (SYT6), transcript variant 1, mRNA.
**23216**	TBC1D1	Homo sapiens TBC1 domain family member 1 (TBC1D1), transcriptvariant 2, mRNA.
**990**	CDC6	Homo sapiens cell division cycle 6 (CDC6), mRNA.
**390**	RND3	Homo sapiens Rho family GTPase 3 (RND3), transcript variant 1,mRNA.
**4628**	MYH10	Homo sapiens myosin heavy chain 10 (MYH10), transcript variant 1,mRNA.
**115908**	CTHRC1	Homo sapiens collagen triple helix repeat containing 1 (CTHRC1),transcript variant 2, mRNA.
**29934**	SNX12	Homo sapiens sorting nexin 12 (SNX12), transcript variant 1, mRNA.
**2107**	ETF1	Homo sapiens eukaryotic translation termination factor 1 (ETF1),transcript variant 2, mRNA.
**2744**	GLS	Homo sapiens glutaminase (GLS), transcript variant 2, mRNA; nucleargene for mitochondrial product.
**126526**	C19orf47	Homo sapiens chromosome 19 open reading frame 47 (C19orf47),transcript variant 1, mRNA.
**9692**	PRORP	Homo sapiens protein only RNase P catalytic subunit (PRORP),transcript variant 2, mRNA; nuclear gene for mitochondrial product.
**51571**	CYRIB	Homo sapiens CYFIP related Rac1 interactor B (CYRIB), transcriptvariant 1, mRNA.
**55143**	CDCA8	Homo sapiens cell division cycle associated 8 (CDCA8), transcriptvariant 1, mRNA.
**3976**	LIF	Homo sapiens LIF interleukin 6 family cytokine (LIF), transcriptvariant 2, mRNA.
**8452**	CUL3	Homo sapiens cullin 3 (CUL3), transcript variant 2, mRNA.
**266629**	SEC14L3	Homo sapiens SEC14 like lipid binding 3 (SEC14L3), transcriptvariant 2, mRNA.
**22806**	IKZF3	Homo sapiens IKAROS family zinc finger 3 (IKZF3), transcriptvariant 7, mRNA.
**27037**	TRMT2A	Homo sapiens tRNA methyltransferase 2 homolog A (TRMT2A),transcript variant 3, mRNA.
**51479**	ANKFY1	Homo sapiens ankyrin repeat and FYVE domain containing 1 (ANKFY1),transcript variant 3, mRNA.
**2036**	EPB41L1	Homo sapiens erythrocyte membrane protein band 4.1 like 1(EPB41L1), transcript variant 3, mRNA.
**81570**	CLPB	Homo sapiens caseinolytic mitochondrial matrix peptidase chaperonesubunit B (CLPB), transcript variant 2, mRNA.

**5962**	RDX	Homo sapiens radixin (RDX), transcript variant 1, mRNA.
**5715**	PSMD9	Homo sapiens proteasome 26S subunit, non-ATPase 9 (PSMD9),transcript variant 2, mRNA.
**5170**	PDPK1	Homo sapiens 3-phosphoinositide dependent protein kinase 1 (PDPK1),transcript variant 3, mRNA.
**1045**	CDX2	Homo sapiens caudal type homeobox 2 (CDX2), transcript variant 1,mRNA.
**9784**	SNX17	Homo sapiens sorting nexin 17 (SNX17), transcript variant 2, mRNA.
**9055**	PRC1	Homo sapiens protein regulator of cytokinesis 1 (PRC1), transcriptvariant 4, mRNA.
**80227**	PAAF1	Homo sapiens proteasomal ATPase associated factor 1 (PAAF1),transcript variant 1, mRNA.
**157567**	ANKRD46	Homo sapiens ankyrin repeat domain 46 (ANKRD46), transcript variant1, mRNA.
**5717**	PSMD11	Homo sapiens proteasome 26S subunit, non-ATPase 11 (PSMD11),transcript variant 1, mRNA.
**11214**	AKAP13	Homo sapiens A-kinase anchoring protein 13 (AKAP13), transcriptvariant 4, mRNA.
**10299**	MARCHF6	Homo sapiens membrane associated ring-CH-type finger 6 (MARCHF6),transcript variant 2, mRNA.
**90550**	MCU	Homo sapiens mitochondrial calcium uniporter (MCU), transcriptvariant 2, mRNA; nuclear gene for mitochondrial product.
**11270**	NRM	Homo sapiens nurim (NRM), transcript variant 2, mRNA.
**54801**	HAUS6	Homo sapiens HAUS augmin like complex subunit 6 (HAUS6), transcriptvariant 2, mRNA.
**8609**	KLF7	Homo sapiens Kruppel like factor 7 (KLF7), transcript variant 2,mRNA.
**4209**	MEF2D	Homo sapiens myocyte enhancer factor 2D (MEF2D), transcript variant2, mRNA.
**9125**	CNOT9	Homo sapiens CCR4-NOT transcription complex subunit 9 (CNOT9),transcript variant 1, mRNA.
**10412**	NSA2	Homo sapiens NSA2 ribosome biogenesis factor (NSA2), transcriptvariant 2, mRNA.
**22818**	COPZ1	Homo sapiens COPI coat complex subunit zeta 1 (COPZ1), transcriptvariant 2, mRNA.
**6198**	RPS6KB1	Homo sapiens ribosomal protein S6 kinase B1 (RPS6KB1), transcriptvariant 2, mRNA.
**5573**	PRKAR1A	Homo sapiens protein kinase cAMP-dependent type I regulatorysubunit alpha (PRKAR1A), transcript variant 4, mRNA.
**55432**	YOD1	Homo sapiens YOD1 deubiquitinase (YOD1), transcript variant 2,mRNA.
**6392**	SDHD	Homo sapiens succinate dehydrogenase complex subunit D (SDHD),transcript variant 2, mRNA; nuclear gene for mitochondrial product.
**9140**	ATG12	Homo sapiens autophagy related 12 (ATG12), transcript variant 5,mRNA.
**6786**	STIM1	Homo sapiens stromal interaction molecule 1 (STIM1), transcriptvariant 1, mRNA.
**55222**	LRRC20	Homo sapiens leucine rich repeat containing 20 (LRRC20), transcriptvariant 4, mRNA.
**1876**	E2F6	Homo sapiens E2F transcription factor 6 (E2F6), transcript variantb, mRNA.
**7551**	ZNF3	Homo sapiens zinc finger protein 3 (ZNF3), transcript variant 3,mRNA.
**9631**	NUP155	Homo sapiens nucleoporin 155 (NUP155), transcript variant 3, mRNA.
**2649**	NR6A1	Homo sapiens nuclear receptor subfamily 6 group A member 1 (NR6A1),transcript variant 4, mRNA.
**5599**	MAPK8	Homo sapiens mitogen-activated protein kinase 8 (MAPK8), transcriptvariant JNK1-b2, mRNA.
**5163**	PDK1	Homo sapiens pyruvate dehydrogenase kinase 1 (PDK1), transcriptvariant 1, mRNA; nuclear gene for mitochondrial product.
**92**	ACVR2A	Homo sapiens activin A receptor type 2A (ACVR2A), transcriptvariant 1, mRNA.
**4772**	NFATC1	Homo sapiens nuclear factor of activated T cells 1 (NFATC1),transcript variant 6, mRNA.
**8110**	DPF3	Homo sapiens double PHD fingers 3 (DPF3), transcript variant 2,mRNA.
**6836**	SURF4	Homo sapiens surfeit 4 (SURF4), transcript variant 2, mRNA.
**9415**	FADS2	Homo sapiens fatty acid desaturase 2 (FADS2), transcript variant 2,mRNA.
**54892**	NCAPG2	Homo sapiens non-SMC condensin II complex subunit G2 (NCAPG2),transcript variant 2, mRNA.
**57502**	NLGN4X	Homo sapiens neuroligin 4 X-linked (NLGN4X), transcript variant 3,mRNA.
**10280**	SIGMAR1	Homo sapiens sigma non-opioid intracellular receptor 1 (SIGMAR1),transcript variant 6, mRNA.
**11194**	ABCB8	Homo sapiens ATP binding cassette subfamily B member 8 (ABCB8),transcript variant 1, mRNA; nuclear gene for mitochondrial product.

**23394**	ADNP	Homo sapiens activity dependent neuroprotector homeobox (ADNP),transcript variant 3, mRNA.
**55031**	USP47	Homo sapiens ubiquitin specific peptidase 47 (USP47), transcriptvariant 1, mRNA.
**79791**	FBXO31	Homo sapiens F-box protein 31 (FBXO31), transcript variant 2, mRNA.
**84898**	PLXDC2	Homo sapiens plexin domain containing 2 (PLXDC2), transcriptvariant 2, mRNA.
**57504**	MTA3	Homo sapiens metastasis associated 1 family member 3 (MTA3),transcript variant 1, mRNA.
**219771**	CCNY	Homo sapiens cyclin Y (CCNY), transcript variant 3, mRNA.
**54464**	XRN1	Homo sapiens 5'-3' exoribonuclease 1 (XRN1), transcript variant 3,mRNA.
**7227**	TRPS1	Homo sapiens transcriptional repressor GATA binding 1 (TRPS1),transcript variant 2, mRNA.
**10152**	ABI2	Homo sapiens abl interactor 2 (ABI2), transcript variant 1, mRNA.
**1174**	AP1S1	Homo sapiens adaptor related protein complex 1 subunit sigma 1(AP1S1), mRNA.
**23308**	ICOSLG	Homo sapiens inducible T cell costimulator ligand (ICOSLG),transcript variant 2, mRNA.
**9320**	TRIP12	Homo sapiens thyroid hormone receptor interactor 12 (TRIP12),transcript variant 1, mRNA.
**26037**	SIPA1L1	Homo sapiens signal induced proliferation associated 1 like 1(SIPA1L1), transcript variant 2, mRNA.
**83606**	GUCD1	Homo sapiens guanylyl cyclase domain containing 1 (GUCD1),transcript variant 1, mRNA.
**11011**	TLK2	Homo sapiens tousled like kinase 2 (TLK2), transcript variant C,mRNA.
**8669**	EIF3J	Homo sapiens eukaryotic translation initiation factor 3 subunit J(EIF3J), transcript variant 2, mRNA.
**4734**	NEDD4	Homo sapiens NEDD4 E3 ubiquitin protein ligase (NEDD4), transcriptvariant 3, mRNA.
**79810**	PTCD2	Homo sapiens pentatricopeptide repeat domain 2 (PTCD2), transcriptvariant 2, mRNA; nuclear gene for mitochondrial product.
**84135**	UTP15	Homo sapiens UTP15 small subunit processome component (UTP15),transcript variant 2, mRNA.
**22828**	SCAF8	Homo sapiens SR-related CTD associated factor 8 (SCAF8), transcriptvariant 1, mRNA.
**134637**	ADAT2	Homo sapiens adenosine deaminase tRNA specific 2 (ADAT2),transcript variant 2, mRNA.
**57380**	MRS2	Homo sapiens magnesium transporter MRS2 (MRS2), transcript variant1, mRNA; nuclear gene for mitochondrial product.
**7178**	TPT1	Homo sapiens tumor protein, translationally-controlled 1 (TPT1),transcript variant 1, mRNA.
**4833**	NME4	Homo sapiens NME/NM23 nucleoside diphosphate kinase 4 (NME4),transcript variant 2, mRNA; nuclear gene for mitochondrial product.
**9750**	RIPOR2	Homo sapiens RHO family interacting cell polarization regulator 2(RIPOR2), transcript variant 3, mRNA.
**51390**	AIG1	Homo sapiens androgen induced 1 (AIG1), transcript variant 2, mRNA.
**221477**	C6orf89	Homo sapiens chromosome 6 open reading frame 89 (C6orf89),transcript variant 2, mRNA.
**2180**	ACSL1	Homo sapiens acyl-CoA synthetase long chain family member 1(ACSL1), transcript variant 2, mRNA.
**79774**	GRTP1	Homo sapiens growth hormone regulated TBC protein 1 (GRTP1),transcript variant 2, mRNA.
**262**	1 AMD	Homo sapiens adenosylmethionine decarboxylase 1 (AMD1), transcriptvariant 3, mRNA.
**129401**	NUP35	Homo sapiens nucleoporin 35 (NUP35), transcript variant 3, mRNA.
**55055**	ZWILCH	Homo sapiens zwilch kinetochore protein (ZWILCH), transcriptvariant 2, mRNA.
**57217**	TTC7A	Homo sapiens tetratricopeptide repeat domain 7A (TTC7A), transcriptvariant 1, mRNA.
**8038**	ADAM12	Homo sapiens ADAM metallopeptidase domain 12 (ADAM12), transcriptvariant 3, mRNA.
**221458**	KIF6	Homo sapiens kinesin family member 6 (KIF6), transcript variant 2,mRNA.
**79830**	ZMYM1	Homo sapiens zinc finger MYM-type containing 1 (ZMYM1), transcriptvariant 1, mRNA.
**10382**	TUBB4A	Homo sapiens tubulin beta 4A class IVa (TUBB4A), transcript variant1, mRNA.
**5045**	FURIN	Homo sapiens furin, paired basic amino acid cleaving enzyme(FURIN), transcript variant 2, mRNA.
**6651**	SON	Homo sapiens SON DNA and RNA binding protein (SON), transcriptvariant e, mRNA.
**7182**	NR2C2	Homo sapiens nuclear receptor subfamily 2 group C member 2 (NR2C2),transcript variant 2, mRNA.
**5966**	REL	Homo sapiens REL proto-oncogene, NF-kB subunit (REL), transcriptvariant 2, mRNA.
**55544**	RBM38	Homo sapiens RNA binding motif protein 38 (RBM38), transcriptvariant 3, mRNA.

**2590**	GALNT2	Homo sapiens polypeptide N-acetylgalactosaminyltransferase 2(GALNT2), transcript variant 2, mRNA.
**8602**	NOP14	Homo sapiens NOP14 nucleolar protein (NOP14), transcript variant 1,mRNA.
**55296**	TBC1D19	Homo sapiens TBC1 domain family member 19 (TBC1D19), transcriptvariant 2, mRNA.
**4670**	HNRNPM	Homo sapiens heterogeneous nuclear ribonucleoprotein M (HNRNPM),transcript variant 3, mRNA.
**11127**	KIF3A	Homo sapiens kinesin family member 3A (KIF3A), transcript variant1, mRNA.
**79829**	NAA40	Homo sapiens N-alpha-acetyltransferase 40, NatD catalytic subunit(NAA40), transcript variant 2, mRNA.
**4188**	MDFI	Homo sapiens MyoD family inhibitor (MDFI), transcript variant 1,mRNA.
**8091**	HMGA2	Homo sapiens high mobility group AT-hook 2 (HMGA2), transcriptvariant 3, mRNA.
**8089**	YEATS4	Homo sapiens YEATS domain containing 4 (YEATS4), transcript variant2, mRNA.
**11100**	HNRNPUL1	Homo sapiens heterogeneous nuclear ribonucleoprotein U like 1(HNRNPUL1), transcript variant 5, mRNA.
**2077**	ERF	Homo sapiens ETS2 repressor factor (ERF), transcript variant 2,mRNA.
**9444**	QKI	Homo sapiens QKI, KH domain containing RNA binding (QKI),transcript variant 5, mRNA.
**23601**	CLEC5A	Homo sapiens C-type lectin domain containing 5A (CLEC5A),transcript variant 2, mRNA.
**57761**	TRIB3	Homo sapiens tribbles pseudokinase 3 (TRIB3), transcript variant 2,mRNA.
**1503**	CTPS1	Homo sapiens CTP synthase 1 (CTPS1), transcript variant 2, mRNA.
**1236**	CCR7	Homo sapiens C-C motif chemokine receptor 7 (CCR7), transcriptvariant 2, mRNA.
**131965**	METTL6	Homo sapiens methyltransferase 6, methylcytidine (METTL6),transcript variant 2, mRNA.
**64900**	LPIN3	Homo sapiens lipin 3 (LPIN3), transcript variant 1, mRNA.
**51621**	KLF13	Homo sapiens Kruppel like factor 13 (KLF13), transcript variant 2,mRNA.
**10627**	MYL12A	Homo sapiens myosin light chain 12A (MYL12A), transcript variant 2,mRNA.
**222068**	TMED4	Homo sapiens transmembrane p24 trafficking protein 4 (TMED4),transcript variant 2, mRNA.
**9026**	HIP1R	Homo sapiens huntingtin interacting protein 1 related (HIP1R),transcript variant 2, mRNA.
**22880**	MORC2	Homo sapiens MORC family CW-type zinc finger 2 (MORC2), transcriptvariant 1, mRNA.
**9819**	TSC22D2	Homo sapiens TSC22 domain family member 2 (TSC22D2), transcriptvariant 2, mRNA.
**55197**	RPRD1A	Homo sapiens regulation of nuclear pre-mRNA domain containing 1A(RPRD1A), transcript variant 2, mRNA.
**10907**	TXNL4A	Homo sapiens thioredoxin like 4A (TXNL4A), transcript variant 2,mRNA.
**84440**	RAB11FIP4	Homo sapiens RAB11 family interacting protein 4 (RAB11FIP4),transcript variant 2, mRNA.
**84269**	CHCHD5	Homo sapiens coiled-coil-helix-coiled-coil-helix domain containing5 (CHCHD5), transcript variant 2, mRNA.
**63917**	GALNT11	Homo sapiens polypeptide N-acetylgalactosaminyltransferase 11(GALNT11), transcript variant 2, mRNA.
**2043**	EPHA4	Homo sapiens EPH receptor A4 (EPHA4), transcript variant 1, mRNA.
**55623**	THUMPD1	Homo sapiens THUMP domain containing 1 (THUMPD1), transcriptvariant 2, mRNA.
**54471**	MIEF1	Homo sapiens mitochondrial elongation factor 1 (MIEF1), transcriptvariant 2, mRNA; nuclear gene for mitochondrial product.
**79879**	CCDC134	Homo sapiens coiled-coil domain containing 134 (CCDC134),transcript variant 2, mRNA.
**84336**	TMEM101	Homo sapiens transmembrane protein 101 (TMEM101), transcriptvariant 2, mRNA.
**124491**	TMEM170A	Homo sapiens transmembrane protein 170A (TMEM170A), transcriptvariant 2, mRNA.
**26151**	NAT9	Homo sapiens N-acetyltransferase 9 (putative) (NAT9), transcriptvariant 2, mRNA.
**284273**	PTGR3	Homo sapiens prostaglandin reductase 3 (PTGR3), transcript variant2, mRNA.
**10776**	ARPP19	Homo sapiens cAMP regulated phosphoprotein 19 (ARPP19), transcriptvariant 1, mRNA.
**80854**	SETD7	Homo sapiens SET domain containing 7, histone lysinemethyltransferase (SETD7), transcript variant 2, mRNA.

**4673**	NAP1L1	Homo sapiens nucleosome assembly protein 1 like 1 (NAP1L1),transcript variant 3, mRNA.
**10620**	ARID3B	Homo sapiens AT-rich interaction domain 3B (ARID3B), transcriptvariant 1, mRNA.
**112399**	EGLN3	Homo sapiens egl-9 family hypoxia inducible factor 3 (EGLN3),transcript variant 2, mRNA.
**54813**	KLHL28	Homo sapiens kelch like family member 28 (KLHL28), transcriptvariant 1, mRNA.
**80315**	CPEB4	Homo sapiens cytoplasmic polyadenylation element binding protein 4(CPEB4), transcript variant 2, mRNA.
**54879**	ST7L	Homo sapiens suppression of tumorigenicity 7 like (ST7L),transcript variant 7, mRNA.
**1009**	CDH11	Homo sapiens cadherin 11 (CDH11), transcript variant 2, mRNA.
**6678**	SPARC	Homo sapiens secreted protein acidic and cysteine rich (SPARC),transcript variant 2, mRNA.
**92667**	MGME1	Homo sapiens mitochondrial genome maintenance exonuclease 1(MGME1), transcript variant 1, mRNA; nuclear gene for mitochondrial
**2149**	F2R	Homo sapiens coagulation factor II thrombin receptor (F2R),transcript variant 2, mRNA.
**10628**	TXNIP	Homo sapiens thioredoxin interacting protein (TXNIP), transcriptvariant 2, mRNA.
**4154**	MBNL1	Homo sapiens muscleblind like splicing regulator 1 (MBNL1),transcript variant 8, mRNA.
**54956**	PARP16	Homo sapiens poly(ADP-ribose) polymerase family member 16 (PARP16),transcript variant 2, mRNA.
**55233**	MOB1A	Homo sapiens MOB kinase activator 1A (MOB1A), transcript variant 2,mRNA.
**54927**	CHCHD3	Homo sapiens coiled-coil-helix-coiled-coil-helix domain containing3 (CHCHD3), transcript variant 1, mRNA.
**999**	CDH1	Homo sapiens cadherin 1 (CDH1), transcript variant 2, mRNA.
**166378**	SPATA5	Homo sapiens spermatogenesis associated 5 (SPATA5), transcriptvariant 2, mRNA.
**10336**	PCGF3	Homo sapiens polycomb group ring finger 3 (PCGF3), transcriptvariant 1, mRNA.
**79850**	TLCD3A	Homo sapiens TLC domain containing 3A (TLCD3A), transcript variant2, mRNA.
**114991**	ZNF618	Homo sapiens zinc finger protein 618 (ZNF618), transcript variant2, mRNA.
**2182**	ACSL4	Homo sapiens acyl-CoA synthetase long chain family member 4(ACSL4), transcript variant 3, mRNA.
**6405**	SEMA3F	Homo sapiens semaphorin 3F (SEMA3F), transcript variant 3, mRNA.
**3159**	HMGA1	Homo sapiens high mobility group AT-hook 1 (HMGA1), transcriptvariant 8, mRNA.
**55734**	ZFP64	Homo sapiens ZFP64 zinc finger protein (ZFP64), transcript variant5, mRNA.
**8556**	CDC14A	Homo sapiens cell division cycle 14A (CDC14A), transcript variant4, mRNA.
**80267**	EDEM3	Homo sapiens ER degradation enhancing alpha-mannosidase likeprotein 3 (EDEM3), transcript variant 1, mRNA.
**8209**	GATD3	Homo sapiens glutamine amidotransferase class 1 domain containing 3(GATD3), transcript variant 3, mRNA; nuclear gene for mitochondrial
**159195**	USP54	Homo sapiens ubiquitin specific peptidase 54 (USP54), transcriptvariant 2, mRNA.
**10066**	SCAMP2	Homo sapiens secretory carrier membrane protein 2 (SCAMP2),transcript variant 1, mRNA.
**1385**	CREB1	Homo sapiens cAMP responsive element binding protein 1 (CREB1),transcript variant 3, mRNA.
**342945**	ZSCAN22	Homo sapiens zinc finger and SCAN domain containing 22 (ZSCAN22),transcript variant 3, mRNA.
**284439**	SLC25A42	Homo sapiens solute carrier family 25 member 42 (SLC25A42),transcript variant 2, mRNA; nuclear gene for mitochondrial product.
**9475**	ROCK2	Homo sapiens Rho associated coiled-coil containing protein kinase 2(ROCK2), transcript variant 2, mRNA.
**55137**	FIGN	Homo sapiens fidgetin, microtubule severing factor (FIGN),transcript variant 2, mRNA.
**60560**	NAA35	Homo sapiens N-alpha-acetyltransferase 35, NatC auxiliary subunit(NAA35), transcript variant 2, mRNA.
**57184**	FAM219B	Homo sapiens family with sequence similarity 219 member B(FAM219B), transcript variant 2, mRNA.
**4705**	NDUFA10	Homo sapiens NADH:ubiquinone oxidoreductase subunit A10 (NDUFA10),transcript variant 2, mRNA; nuclear gene for mitochondrial product.
**55066**	PDPR	Homo sapiens pyruvate dehydrogenase phosphatase regulatory subunit(PDPR), transcript variant 2, mRNA; nuclear gene for mitochondrial

**201626**	PDE12	Homo sapiens phosphodiesterase 12 (PDE12), transcript variant 2,mRNA.
**124540**	MSI2	Homo sapiens musashi RNA binding protein 2 (MSI2), transcriptvariant 3, mRNA.
**57536**	KIAA1328	Homo sapiens KIAA1328 (KIAA1328), transcript variant 2, mRNA.
**80851**	SH3BP5L	Homo sapiens SH3 binding domain protein 5 like (SH3BP5L),transcript variant 2, mRNA.
**4585**	MUC4	Homo sapiens mucin 4, cell surface associated (MUC4), transcriptvariant 6, mRNA.
**80005**	DOCK5	Homo sapiens dedicator of cytokinesis 5 (DOCK5), transcript variant2, mRNA.
**6790**	AURKA	Homo sapiens aurora kinase A (AURKA), transcript variant 7, mRNA.
**899**	CCNF	Homo sapiens cyclin F (CCNF), transcript variant 2, mRNA.
**126626**	GABPB2	Homo sapiens GA binding protein transcription factor subunit beta 2(GABPB2), transcript variant 3, mRNA.
**27334**	P2RY10	Homo sapiens P2Y receptor family member 10 (P2RY10), transcriptvariant 3, mRNA.
**79801**	SHCBP1	Homo sapiens SHC binding and spindle associated 1 (SHCBP1),transcript variant 2, mRNA.
**23593**	HEBP2	Homo sapiens heme binding protein 2 (HEBP2), transcript variant 1,mRNA.
**91754**	NEK9	Homo sapiens NIMA related kinase 9 (NEK9), transcript variant 1,mRNA.
**84908**	FAM136A	Homo sapiens family with sequence similarity 136 member A(FAM136A), transcript variant 2, mRNA.
**26995**	TRUB2	Homo sapiens TruB pseudouridine synthase family member 2 (TRUB2),transcript variant 2, mRNA; nuclear gene for mitochondrial product.
**57546**	PDP2	Homo sapiens pyruvate dehyrogenase phosphatase catalytic subunit 2(PDP2), transcript variant 2, mRNA; nuclear gene for mitochondrial
**7043**	TGFB3	Homo sapiens transforming growth factor beta 3 (TGFB3), transcriptvariant 3, mRNA.
**90957**	DHX57	Homo sapiens DExH-box helicase 57 (DHX57), transcript variant 2,mRNA.
**134492**	NUDCD2	Homo sapiens NudC domain containing 2 (NUDCD2), transcript variant2, mRNA.
**222171**	PRR15	Homo sapiens proline rich 15 (PRR15), transcript variant 2, mRNA.
**162394**	SLFN5	Homo sapiens schlafen family member 5 (SLFN5), transcript variant2, mRNA.
**84299**	MIEN1	Homo sapiens migration and invasion enhancer 1 (MIEN1), transcriptvariant 2, mRNA.
**4665**	NAB2	Homo sapiens NGFI-A binding protein 2 (NAB2), transcript variant 2,mRNA.
**5977**	DPF2	Homo sapiens double PHD fingers 2 (DPF2), transcript variant 2,mRNA.
**57508**	INTS2	Homo sapiens integrator complex subunit 2 (INTS2), transcriptvariant 3, mRNA.
**124402**	UBALD1	Homo sapiens UBA like domain containing 1 (UBALD1), transcriptvariant 2, mRNA.
**1912**	PHC2	Homo sapiens polyhomeotic homolog 2 (PHC2), transcript variant 3,mRNA.
**51253**	MRPL37	Homo sapiens mitochondrial ribosomal protein L37 (MRPL37),transcript variant 1, mRNA; nuclear gene for mitochondrial product.
**92106**	OXNAD1	Homo sapiens oxidoreductase NAD binding domain containing 1(OXNAD1), transcript variant 1, mRNA.
**10891**	PPARGC1A	Homo sapiens PPARG coactivator 1 alpha (PPARGC1A), transcriptvariant 1, mRNA.
**11064**	CNTRL	Homo sapiens centriolin (CNTRL), transcript variant 2, mRNA.
**152100**	CMC1	Homo sapiens C-X9-C motif containing 1 (CMC1), transcript variant1, mRNA; nuclear gene for mitochondrial product.
**1603**	DAD1	Homo sapiens defender against cell death 1 (DAD1), mRNA.
**389084**	SNORC	Homo sapiens secondary ossification center associated regulator ofchondrocyte maturation (SNORC), transcript variant 1, mRNA.
**57522**	SRGAP1	Homo sapiens SLIT-ROBO Rho GTPase activating protein 1 (SRGAP1),transcript variant 2, mRNA.
**51585**	PCF11	Homo sapiens PCF11 cleavage and polyadenylation factor subunit(PCF11), transcript variant 1, mRNA.
**23029**	RBM34	Homo sapiens RNA binding motif protein 34 (RBM34), transcriptvariant 4, mRNA.
**399687**	MYO18A	Homo sapiens myosin XVIIIA (MYO18A), transcript variant 3, mRNA.
**1956**	EGFR	Homo sapiens epidermal growth factor receptor (EGFR), transcriptvariant 5, mRNA.
**80095**	ZNF606	Homo sapiens zinc finger protein 606 (ZNF606), transcript variant2, mRNA.

**57608**	JCAD	Homo sapiens junctional cadherin 5 associated (JCAD), transcriptvariant 2, mRNA.
**6789**	STK4	Homo sapiens serine/threonine kinase 4 (STK4), transcript variant2, mRNA.
**64434**	NOM1	Homo sapiens nucleolar protein with MIF4G domain 1 (NOM1),transcript variant 2, mRNA.
**79794**	SPRING1	Homo sapiens SREBF pathway regulator in golgi 1 (SPRING1),transcript variant 2, mRNA.
**7074**	TIAM1	Homo sapiens TIAM Rac1 associated GEF 1 (TIAM1), transcript variant2, mRNA.
**3693**	ITGB5	Homo sapiens integrin subunit beta 5 (ITGB5), transcript variant 2,mRNA.
**4609**	MYC	Homo sapiens MYC proto-oncogene, bHLH transcription factor (MYC),transcript variant 2, mRNA.
**148203**	ZNF738	Homo sapiens zinc finger protein 738 (ZNF738), transcript variant1, mRNA.
**1457**	CSNK2A1	Homo sapiens casein kinase 2 alpha 1 (CSNK2A1), transcript variant4, mRNA.
**79864**	JHY	Homo sapiens junctional cadherin complex regulator (JHY),transcript variant 3, mRNA.
**92140**	MTDH	Homo sapiens metadherin (MTDH), transcript variant 2, mRNA.
**137964**	GPAT4	Homo sapiens glycerol-3-phosphate acyltransferase 4 (GPAT4),transcript variant 2, mRNA.
**84668**	FAM126A	Homo sapiens family with sequence similarity 126 member A(FAM126A), transcript variant 2, mRNA.
**56829**	ZC3HAV1	Homo sapiens zinc finger CCCH-type containing, antiviral 1(ZC3HAV1), transcript variant 3, mRNA.
**57562**	CEP126	Homo sapiens centrosomal protein 126 (CEP126), transcript variant2, mRNA.
**7371**	UCK2	Homo sapiens uridine-cytidine kinase 2 (UCK2), transcript variant2, mRNA.
**91289**	LMF2	Homo sapiens lipase maturation factor 2 (LMF2), transcript variant2, mRNA.
**900**	CCNG1	Homo sapiens cyclin G1 (CCNG1), transcript variant 3, mRNA.
**23095**	KIF1B	Homo sapiens kinesin family member 1B (KIF1B), transcript variant3, mRNA.
**8570**	KHSRP	Homo sapiens KH-type splicing regulatory protein (KHSRP),transcript variant 2, mRNA.
**3841**	KPNA5	Homo sapiens karyopherin subunit alpha 5 (KPNA5), transcriptvariant 1, mRNA.
**91010**	FMNL3	Homo sapiens formin like 3 (FMNL3), transcript variant 3, mRNA.
**10825**	NEU3	Homo sapiens neuraminidase 3 (NEU3), transcript variant 2, mRNA.
**865**	CBFB	Homo sapiens core-binding factor subunit beta (CBFB), transcriptvariant 3, mRNA.
**5899**	RALB	Homo sapiens RAS like proto-oncogene B (RALB), transcript variant2, mRNA.
**6774**	STAT3	Homo sapiens signal transducer and activator of transcription 3(STAT3), transcript variant 4, mRNA.
**7576**	ZNF28	Homo sapiens zinc finger protein 28 (ZNF28), transcript variant 2,mRNA.
**3845**	KRAS	Homo sapiens KRAS proto-oncogene, GTPase (KRAS), transcript variantc, mRNA.
**84939**	PWWP3A	Homo sapiens PWWP domain containing 3A, DNA repair factor (PWWP3A),transcript variant 3, mRNA.
**149076**	ZNF362	Homo sapiens zinc finger protein 362 (ZNF362), transcript variant2, mRNA.
**9031**	BAZ1B	Homo sapiens bromodomain adjacent to zinc finger domain 1B (BAZ1B),transcript variant 1, mRNA.
**55159**	RFWD3	Homo sapiens ring finger and WD repeat domain 3 (RFWD3), transcriptvariant 2, mRNA.
**8454**	CUL1	Homo sapiens cullin 1 (CUL1), transcript variant 2, mRNA.
**80704**	SLC19A3	Homo sapiens solute carrier family 19 member 3 (SLC19A3),transcript variant 2, mRNA.
**4249**	MGAT5	Homo sapiens alpha-1,6-mannosylglycoprotein6-beta-N-acetylglucosaminyltransferase (MGAT5), transcript variant
**1948**	EFNB2	Homo sapiens ephrin B2 (EFNB2), transcript variant 2, mRNA.
**7402**	UTRN	Homo sapiens utrophin (UTRN), transcript variant 2, mRNA.
**64393**	ZMAT3	Homo sapiens zinc finger matrin-type 3 (ZMAT3), transcript variant3, mRNA.
**1778**	DYNC1H1	Homo sapiens dynein cytoplasmic 1 heavy chain 1 (DYNC1H1), mRNA.
**57583**	TMEM181	Homo sapiens transmembrane protein 181 (TMEM181), transcriptvariant 1, mRNA.
**200081**	TXLNA	Homo sapiens taxilin alpha (TXLNA), transcript variant 2, mRNA.
**8740**	TNFSF14	Homo sapiens TNF superfamily member 14 (TNFSF14), transcriptvariant 3, mRNA.

**5287**	PIK3C2B	Homo sapiens phosphatidylinositol-4-phosphate 3-kinase catalyticsubunit type 2 beta (PIK3C2B), transcript variant 1, mRNA.
**389677**	RBM12B	Homo sapiens RNA binding motif protein 12B (RBM12B), transcriptvariant 1, mRNA.
**283149**	BCL9L	Homo sapiens BCL9 like (BCL9L), transcript variant 1, mRNA.
**9759**	HDAC4	Homo sapiens histone deacetylase 4 (HDAC4), transcript variant 1,mRNA.
**2683**	B4GALT1	Homo sapiens beta-1,4-galactosyltransferase 1 (B4GALT1), transcriptvariant 2, mRNA.
**123041**	SLC24A4	Homo sapiens solute carrier family 24 member 4 (SLC24A4),transcript variant 4, mRNA.
**30001**	ERO1A	Homo sapiens endoplasmic reticulum oxidoreductase 1 alpha (ERO1A),transcript variant 1, mRNA.
**6773**	STAT2	Homo sapiens signal transducer and activator of transcription 2(STAT2), transcript variant 3, mRNA.
**7803**	PTP4A1	Homo sapiens protein tyrosine phosphatase 4A1 (PTP4A1), transcriptvariant 2, mRNA.
**11122**	PTPRT	Homo sapiens protein tyrosine phosphatase receptor type T (PTPRT),transcript variant 3, mRNA.
**9908**	G3BP2	Homo sapiens G3BP stress granule assembly factor 2 (G3BP2),transcript variant 4, mRNA.
**7416**	VDAC1	Homo sapiens voltage dependent anion channel 1 (VDAC1), transcriptvariant 4, mRNA.
**2309**	FOXO3	Homo sapiens forkhead box O3 (FOXO3), transcript variant 1, mRNA.
**196**	AHR	Homo sapiens aryl hydrocarbon receptor (AHR), mRNA.
**363**	AQP6	Homo sapiens aquaporin 6 (AQP6), mRNA.
**391**	RHOG	Homo sapiens ras homolog family member G (RHOG), mRNA.
**894**	CCND2	Homo sapiens cyclin D2 (CCND2), mRNA.
**1006**	CDH8	Homo sapiens cadherin 8 (CDH8), mRNA.
**1295**	COL8A1	Homo sapiens collagen type VIII alpha 1 chain (COL8A1), transcriptvariant 1, mRNA.
**1349**	COX7B	Homo sapiens cytochrome c oxidase subunit 7B (COX7B), mRNA; nucleargene for mitochondrial product.
**1825**	DSC3	Homo sapiens desmocollin 3 (DSC3), transcript variant Dsc3a, mRNA.
**1829**	DSG2	Homo sapiens desmoglein 2 (DSG2), mRNA.
**2288**	FKBP4	Homo sapiens FKBP prolyl isomerase 4 (FKBP4), mRNA.
**2329**	FMO4	Homo sapiens flavin containing dimethylaniline monoxygenase 4(FMO4), mRNA.
**2526**	FUT4	Homo sapiens fucosyltransferase 4 (FUT4), mRNA.
**2740**	GLP1R	Homo sapiens glucagon like peptide 1 receptor (GLP1R), transcriptvariant 1, mRNA.
**3308**	HSPA4	Homo sapiens heat shock protein family A (Hsp70) member 4 (HSPA4),mRNA.
**3675**	ITGA3	Homo sapiens integrin subunit alpha 3 (ITGA3), mRNA.
**3833**	KIFC1	Homo sapiens kinesin family member C1 (KIFC1), mRNA.
**4884**	NPTX1	Homo sapiens neuronal pentraxin 1 (NPTX1), mRNA.
**4893**	NRAS	Homo sapiens NRAS proto-oncogene, GTPase (NRAS), mRNA.
**5155**	PDGFB	Homo sapiens platelet derived growth factor subunit B (PDGFB),transcript variant 1, mRNA.
**5252**	PHF1	Homo sapiens PHD finger protein 1 (PHF1), transcript variant 1,mRNA.
**5326**	PLAGL2	Homo sapiens PLAG1 like zinc finger 2 (PLAGL2), mRNA.
**5336**	PLCG2	Homo sapiens phospholipase C gamma 2 (PLCG2), mRNA.
**5603**	MAPK13	Homo sapiens mitogen-activated protein kinase 13 (MAPK13),transcript variant 1, mRNA.
**5719**	PSMD13	Homo sapiens proteasome 26S subunit, non-ATPase 13 (PSMD13),transcript variant 1, mRNA.
**6095**	RORA	Homo sapiens RAR related orphan receptor A (RORA), transcriptvariant 3, mRNA.
**6422**	SFRP1	Homo sapiens secreted frizzled related protein 1 (SFRP1), mRNA.
**6482**	ST3GAL1	Homo sapiens ST3 beta-galactoside alpha-2,3-sialyltransferase 1(ST3GAL1), transcript variant 1, mRNA.
**6524**	SLC5A2	Homo sapiens solute carrier family 5 member 2 (SLC5A2), transcriptvariant 1, mRNA.

**6599**	SMARCC1	Homo sapiens SWI/SNF related, matrix associated, actin dependentregulator of chromatin subfamily c member 1 (SMARCC1), mRNA.
**6659**	SOX4	Homo sapiens SRY-box transcription factor 4 (SOX4), mRNA.
**6711**	SPTBN1	Homo sapiens spectrin beta, non-erythrocytic 1 (SPTBN1), transcriptvariant 1, mRNA.
**7057**	THBS1	Homo sapiens thrombospondin 1 (THBS1), mRNA.
**7317**	UBA1	Homo sapiens ubiquitin like modifier activating enzyme 1 (UBA1),transcript variant 1, mRNA.
**7322**	UBE2D2	Homo sapiens ubiquitin conjugating enzyme E2 D2 (UBE2D2),transcript variant 1, mRNA.
**7329**	UBE2I	Homo sapiens ubiquitin conjugating enzyme E2 I (UBE2I), transcriptvariant 1, mRNA.
**7414**	VCL	Homo sapiens vinculin (VCL), transcript variant 2, mRNA.
**7479**	WNT8B	Homo sapiens Wnt family member 8B (WNT8B), mRNA.
**9422**	ZNF264	Homo sapiens zinc finger protein 264 (ZNF264), mRNA.
**8578**	SCARF1	Homo sapiens scavenger receptor class F member 1 (SCARF1),transcript variant 1, mRNA.
**8614**	STC2	Homo sapiens stanniocalcin 2 (STC2), mRNA.
**8651**	SOCS1	Homo sapiens suppressor of cytokine signaling 1 (SOCS1), mRNA.
**8661**	EIF3A	Homo sapiens eukaryotic translation initiation factor 3 subunit A(EIF3A), mRNA.
**8664**	EIF3D	Homo sapiens eukaryotic translation initiation factor 3 subunit D(EIF3D), transcript variant 1, mRNA.
**8739**	HRK	Homo sapiens harakiri, BCL2 interacting protein (HRK), transcriptvariant 1, mRNA.
**8744**	TNFSF9	Homo sapiens TNF superfamily member 9 (TNFSF9), mRNA.
**8773**	SNAP23	Homo sapiens synaptosome associated protein 23 (SNAP23), transcriptvariant 1, mRNA.
**8795**	TNFRSF10B	Homo sapiens TNF receptor superfamily member 10b (TNFRSF10B),transcript variant 1, mRNA.
**8879**	SGPL1	Homo sapiens sphingosine-1-phosphate lyase 1 (SGPL1), mRNA.
**8932**	MBD2	Homo sapiens methyl-CpG binding domain protein 2 (MBD2), transcriptvariant 1, mRNA.
**388**	RHOB	Homo sapiens ras homolog family member B (RHOB), mRNA.
**1870**	E2F2	Homo sapiens E2F transcription factor 2 (E2F2), mRNA.
**9294**	S1PR2	Homo sapiens sphingosine-1-phosphate receptor 2 (S1PR2), mRNA.
**9306**	SOCS6	Homo sapiens suppressor of cytokine signaling 6 (SOCS6), mRNA.
**91**	ACVR1B	Homo sapiens activin A receptor type 1B (ACVR1B), transcriptvariant 1, mRNA.
**657**	BMPR1A	Homo sapiens bone morphogenetic protein receptor type 1A (BMPR1A),mRNA.
**997**	CDC34	Homo sapiens cell division cycle 34, ubiqiutin conjugating enzyme(CDC34), mRNA.
**1676**	DFFA	Homo sapiens DNA fragmentation factor subunit alpha (DFFA),transcript variant 1, mRNA.
**1857**	DVL3	Homo sapiens dishevelled segment polarity protein 3 (DVL3), mRNA.
**1947**	EFNB1	Homo sapiens ephrin B1 (EFNB1), mRNA.
**3192**	HNRNPU	Homo sapiens heterogeneous nuclear ribonucleoprotein U (HNRNPU),transcript variant 2, mRNA.
**4775**	NFATC3	Homo sapiens nuclear factor of activated T cells 3 (NFATC3),transcript variant 2, mRNA.
**5195**	PEX14	Homo sapiens peroxisomal biogenesis factor 14 (PEX14), mRNA.
**7189**	TRAF6	Homo sapiens TNF receptor associated factor 6 (TRAF6), transcriptvariant 2, mRNA.
**9145**	SYNGR1	Homo sapiens synaptogyrin 1 (SYNGR1), transcript variant 1a, mRNA.
**9146**	HGS	Homo sapiens hepatocyte growth factor-regulated tyrosine kinasesubstrate (HGS), mRNA.
**9341**	VAMP3	Homo sapiens vesicle associated membrane protein 3 (VAMP3), mRNA.
**5433**	POLR2D	Homo sapiens RNA polymerase II subunit D (POLR2D), mRNA.
**9409**	PEX16	Homo sapiens peroxisomal biogenesis factor 16 (PEX16), transcriptvariant 1, mRNA.
**4363**	ABCC1	Homo sapiens ATP binding cassette subfamily C member 1 (ABCC1),mRNA.
**4643**	MYO1E	Homo sapiens myosin IE (MYO1E), mRNA.
**6319**	SCD	Homo sapiens stearoyl-CoA desaturase (SCD), mRNA.

**867**	CBL	Homo sapiens Cbl proto-oncogene (CBL), mRNA.
**1820**	ARID3A	Homo sapiens AT-rich interaction domain 3A (ARID3A), mRNA.
**3005**	H1-0	Homo sapiens H1.0 linker histone (H1-0), mRNA.
**3021**	H3-3B	Homo sapiens H3.3 histone B (H3-3B), mRNA.
**4089**	SMAD4	Homo sapiens SMAD family member 4 (SMAD4), mRNA.
**4352**	MPL	Homo sapiens MPL proto-oncogene, thrombopoietin receptor (MPL),mRNA.
**6574**	SLC20A1	Homo sapiens solute carrier family 20 member 1 (SLC20A1), mRNA.
**8208**	CHAF1B	Homo sapiens chromatin assembly factor 1 subunit B (CHAF1B), mRNA.
**3151**	HMGN2	Homo sapiens high mobility group nucleosomal binding domain 2(HMGN2), mRNA.
**3419**	IDH3A	Homo sapiens isocitrate dehydrogenase (NAD(+)) 3 catalytic subunitalpha (IDH3A), mRNA; nuclear gene for mitochondrial product.
**4091**	SMAD6	Homo sapiens SMAD family member 6 (SMAD6), transcript variant 1,mRNA.
**7014**	TERF2	Homo sapiens telomeric repeat binding factor 2 (TERF2), mRNA.
**9290**	GPR55	Homo sapiens G protein-coupled receptor 55 (GPR55), mRNA.
**10106**	CTDSP2	Homo sapiens CTD small phosphatase 2 (CTDSP2), mRNA.
**25820**	ARIH1	Homo sapiens ariadne RBR E3 ubiquitin protein ligase 1 (ARIH1),mRNA.
**10184**	LHFPL2	Homo sapiens LHFPL tetraspan subfamily member 2 (LHFPL2), mRNA.
**10238**	DCAF7	Homo sapiens DDB1 and CUL4 associated factor 7 (DCAF7), transcriptvariant 1, mRNA.
**4214**	MAP3K1	Homo sapiens mitogen-activated protein kinase kinase kinase 1(MAP3K1), mRNA.
**7386**	UQCRFS1	Homo sapiens ubiquinol-cytochrome c reductase, Rieske iron-sulfurpolypeptide 1 (UQCRFS1), mRNA; nuclear gene for mitochondrial
**829**	CAPZA1	Homo sapiens capping actin protein of muscle Z-line subunit alpha 1(CAPZA1), mRNA.
**5300**	PIN1	Homo sapiens peptidylprolyl cis/trans isomerase, NIMA-interacting 1(PIN1), transcript variant 1, mRNA.
**5611**	DNAJC3	Homo sapiens DnaJ heat shock protein family (Hsp40) member C3(DNAJC3), mRNA.
**5903**	RANBP2	Homo sapiens RAN binding protein 2 (RANBP2), mRNA.
**10489**	LRRC41	Homo sapiens leucine rich repeat containing 41 (LRRC41), mRNA.
**10527**	IPO7	Homo sapiens importin 7 (IPO7), mRNA.
**10643**	IGF2BP3	Homo sapiens insulin like growth factor 2 mRNA binding protein 3(IGF2BP3), mRNA.
**10723**	SLC12A7	Homo sapiens solute carrier family 12 member 7 (SLC12A7), mRNA.
**10749**	KIF1C	Homo sapiens kinesin family member 1C (KIF1C), mRNA.
**6645**	SNTB2	Homo sapiens syntrophin beta 2 (SNTB2), transcript variant 1, mRNA.
**7832**	BTG2	Homo sapiens BTG anti-proliferation factor 2 (BTG2), mRNA.
**8886**	DDX18	Homo sapiens DEAD-box helicase 18 (DDX18), mRNA.
**10885**	WDR3	Homo sapiens WD repeat domain 3 (WDR3), mRNA.
**10892**	MALT1	Homo sapiens MALT1 paracaspase (MALT1), transcript variant 1, mRNA.
**10955**	SERINC3	Homo sapiens serine incorporator 3 (SERINC3), transcript variant 1,mRNA.
**10957**	PNRC1	Homo sapiens proline rich nuclear receptor coactivator 1 (PNRC1),mRNA.
**10960**	LMAN2	Homo sapiens lectin, mannose binding 2 (LMAN2), mRNA.
**4649**	MYO9A	Homo sapiens myosin IXA (MYO9A), mRNA.
**8013**	NR4A3	Homo sapiens nuclear receptor subfamily 4 group A member 3 (NR4A3),transcript variant 1, mRNA.
**11076**	TPPP	Homo sapiens tubulin polymerization promoting protein (TPPP), mRNA.
**11167**	FSTL1	Homo sapiens follistatin like 1 (FSTL1), mRNA.
**7204**	TRIO	Homo sapiens trio Rho guanine nucleotide exchange factor (TRIO),transcript variant 1, mRNA.
**11198**	SUPT16H	Homo sapiens SPT16 homolog, facilitates chromatin remodelingsubunit (SUPT16H), mRNA.

**11264**	PXMP4	Homo sapiens peroxisomal membrane protein 4 (PXMP4), transcriptvariant 1, mRNA.
**11334**	TUSC2	Homo sapiens tumor suppressor 2, mitochondrial calcium regulator(TUSC2), mRNA.
**11240**	PADI2	Homo sapiens peptidyl arginine deiminase 2 (PADI2), mRNA.
**23452**	ANGPTL2	Homo sapiens angiopoietin like 2 (ANGPTL2), mRNA.
**9329**	GTF3C4	Homo sapiens general transcription factor IIIC subunit 4 (GTF3C4),transcript variant 1, mRNA.
**23633**	KPNA6	Homo sapiens karyopherin subunit alpha 6 (KPNA6), mRNA.
**23556**	PIGN	Homo sapiens phosphatidylinositol glycan anchor biosynthesis classN (PIGN), transcript variant 2, mRNA.
**26292**	MYCBP	Homo sapiens MYC binding protein (MYCBP), transcript variant 1,mRNA.
**26205**	GMEB2	Homo sapiens glucocorticoid modulatory element binding protein 2(GMEB2), mRNA.
**27430**	MAT2B	Homo sapiens methionine adenosyltransferase 2B (MAT2B), transcriptvariant 1, mRNA.
**23192**	ATG4B	Homo sapiens autophagy related 4B cysteine peptidase (ATG4B),transcript variant 1, mRNA.
**7766**	ZNF223	Homo sapiens zinc finger protein 223 (ZNF223), mRNA.
**29777**	ABT1	Homo sapiens activator of basal transcription 1 (ABT1), mRNA.
**11177**	BAZ1A	Homo sapiens bromodomain adjacent to zinc finger domain 1A (BAZ1A),transcript variant 1, mRNA.
**28962**	OSTM1	Homo sapiens osteoclastogenesis associated transmembrane protein 1(OSTM1), mRNA.
**28987**	NOB1	Homo sapiens NIN1 (RPN12) binding protein 1 homolog (NOB1),transcript variant 1, mRNA.
**29095**	ORMDL2	Homo sapiens ORMDL sphingolipid biosynthesis regulator 2 (ORMDL2),mRNA.
**22826**	DNAJC8	Homo sapiens DnaJ heat shock protein family (Hsp40) member C8(DNAJC8), transcript variant 1, mRNA.
**27125**	AFF4	Homo sapiens AF4/FMR2 family member 4 (AFF4), mRNA.
**27143**	PALD1	Homo sapiens phosphatase domain containing paladin 1 (PALD1), mRNA.
**27230**	SERP1	Homo sapiens stress associated endoplasmic reticulum protein 1(SERP1), mRNA.
**27246**	RNF115	Homo sapiens ring finger protein 115 (RNF115), mRNA.
**27284**	SULT1B1	Homo sapiens sulfotransferase family 1B member 1 (SULT1B1), mRNA.
**30837**	SOCS7	Homo sapiens suppressor of cytokine signaling 7 (SOCS7), mRNA.
**9775**	EIF4A3	Homo sapiens eukaryotic translation initiation factor 4A3 (EIF4A3),mRNA.
**1718**	DHCR24	Homo sapiens 24-dehydrocholesterol reductase (DHCR24), mRNA.
**9804**	TOMM20	Homo sapiens translocase of outer mitochondrial membrane 20(TOMM20), mRNA; nuclear gene for mitochondrial product.
**9813**	EFCAB14	Homo sapiens EF-hand calcium binding domain 14 (EFCAB14), mRNA.
**22838**	RNF44	Homo sapiens ring finger protein 44 (RNF44), mRNA.
**22873**	DZIP1	Homo sapiens DAZ interacting zinc finger protein 1 (DZIP1),transcript variant 1, mRNA.
**23060**	ZNF609	Homo sapiens zinc finger protein 609 (ZNF609), mRNA.
**23076**	RRP1B	Homo sapiens ribosomal RNA processing 1B (RRP1B), mRNA.
**23170**	TTLL12	Homo sapiens tubulin tyrosine ligase like 12 (TTLL12), mRNA.
**23224**	SYNE2	Homo sapiens spectrin repeat containing nuclear envelope protein 2(SYNE2), transcript variant 1, mRNA.
**23378**	RRP8	Homo sapiens ribosomal RNA processing 8 (RRP8), mRNA.
**23389**	MED13L	Homo sapiens mediator complex subunit 13L (MED13L), mRNA.
**25885**	POLR1A	Homo sapiens RNA polymerase I subunit A (POLR1A), mRNA.
**26043**	UBXN7	Homo sapiens UBX domain protein 7 (UBXN7), mRNA.
**26156**	RSL1D1	Homo sapiens ribosomal L1 domain containing 1 (RSL1D1), mRNA.
**51614**	ERGIC3	Homo sapiens ERGIC and golgi 3 (ERGIC3), transcript variant 2,mRNA.
**51379**	CRLF3	Homo sapiens cytokine receptor like factor 3 (CRLF3), transcriptvariant 1, mRNA.

**51132**	RLIM	Homo sapiens ring finger protein, LIM domain interacting (RLIM),transcript variant 1, mRNA.
**51160**	VPS28	Homo sapiens VPS28 subunit of ESCRT-I (VPS28), transcript variant1, mRNA.
**51244**	CCDC174	Homo sapiens coiled-coil domain containing 174 (CCDC174),transcript variant 1, mRNA.
**51290**	ERGIC2	Homo sapiens ERGIC and golgi 2 (ERGIC2), mRNA.
**84146**	ZNF644	Homo sapiens zinc finger protein 644 (ZNF644), transcript variant3, mRNA.
**8862**	APLN	Homo sapiens apelin (APLN), mRNA.
**54805**	CNNM2	Homo sapiens cyclin and CBS domain divalent metal cation transportmediator 2 (CNNM2), transcript variant 1, mRNA.
**54863**	TOR4A	Homo sapiens torsin family 4 member A (TOR4A), mRNA.
**54901**	CDKAL1	Homo sapiens CDK5 regulatory subunit associated protein 1 like 1(CDKAL1), mRNA.
**54910**	SEMA4C	Homo sapiens semaphorin 4C (SEMA4C), mRNA.
**54978**	SLC35F6	Homo sapiens solute carrier family 35 member F6 (SLC35F6), mRNA.
**55705**	IPO9	Homo sapiens importin 9 (IPO9), mRNA.
**55161**	TMEM33	Homo sapiens transmembrane protein 33 (TMEM33), mRNA.
**55207**	ARL8B	Homo sapiens ADP ribosylation factor like GTPase 8B (ARL8B), mRNA.
**55236**	UBA6	Homo sapiens ubiquitin like modifier activating enzyme 6 (UBA6),mRNA.
**55333**	SYNJ2BP	Homo sapiens synaptojanin 2 binding protein (SYNJ2BP), mRNA.
**55341**	LSG1	Homo sapiens large 60S subunit nuclear export GTPase 1 (LSG1),mRNA.
**55349**	CHDH	Homo sapiens choline dehydrogenase (CHDH), mRNA; nuclear gene formitochondrial product.
**55813**	UTP6	Homo sapiens UTP6 small subunit processome component (UTP6), mRNA.
**55515**	ASIC4	Homo sapiens acid sensing ion channel subunit family member 4(ASIC4), transcript variant 1, mRNA.
**54069**	MIS18A	Homo sapiens MIS18 kinetochore protein A (MIS18A), mRNA.
**3206**	HOXA10	Homo sapiens homeobox A10 (HOXA10), transcript variant 1, mRNA.
**54328**	GPR173	Homo sapiens G protein-coupled receptor 173 (GPR173), mRNA.
**54491**	OTULINL	Homo sapiens OTU deubiquitinase with linear linkage specificitylike (OTULINL), mRNA.
**54622**	15 ARL	Homo sapiens ADP ribosylation factor like GTPase 15 (ARL15), mRNA.
**65977**	PLEKHA3	Homo sapiens pleckstrin homology domain containing A3 (PLEKHA3),mRNA.
**56261**	GPCPD1	Homo sapiens glycerophosphocholine phosphodiesterase 1 (GPCPD1),mRNA.
**56913**	C1GALT1	Homo sapiens core 1 synthase, glycoprotein-N-acetylgalactosamine3-beta-galactosyltransferase 1 (C1GALT1), mRNA.
**56852**	RAD18	Homo sapiens RAD18 E3 ubiquitin protein ligase (RAD18), mRNA.
**56977**	STOX2	Homo sapiens storkhead box 2 (STOX2), transcript variant 1, mRNA.
**56984**	PSMG2	Homo sapiens proteasome assembly chaperone 2 (PSMG2), transcriptvariant 1, mRNA.
**57221**	ARFGEF3	Homo sapiens ARFGEF family member 3 (ARFGEF3), mRNA.
**57095**	PITHD1	Homo sapiens PITH domain containing 1 (PITHD1), mRNA.
**5738**	PTGFRN	Homo sapiens prostaglandin F2 receptor inhibitor (PTGFRN), mRNA.
**57191**	VN1R1	Homo sapiens vomeronasal 1 receptor 1 (VN1R1), mRNA.
**57459**	GATAD2B	Homo sapiens GATA zinc finger domain containing 2B (GATAD2B), mRNA.
**57484**	RNF150	Homo sapiens ring finger protein 150 (RNF150), mRNA.
**57532**	NUFIP2	Homo sapiens nuclear FMR1 interacting protein 2 (NUFIP2), mRNA.
**57534**	MIB1	Homo sapiens MIB E3 ubiquitin protein ligase 1 (MIB1), mRNA.
**57537**	SORCS2	Homo sapiens sortilin related VPS10 domain containing receptor 2(SORCS2), mRNA.
**57551**	TAOK1	Homo sapiens TAO kinase 1 (TAOK1), transcript variant 1, mRNA.
**57688**	ZSWIM6	Homo sapiens zinc finger SWIM-type containing 6 (ZSWIM6), mRNA.

**9076**	CLDN1	Homo sapiens claudin 1 (CLDN1), mRNA.
**7126**	TNFAIP1	Homo sapiens TNF alpha induced protein 1 (TNFAIP1), mRNA.
**4166**	CHST6	Homo sapiens carbohydrate sulfotransferase 6 (CHST6), transcriptvariant 1, mRNA.
**60681**	FKBP10	Homo sapiens FKBP prolyl isomerase 10 (FKBP10), mRNA.
**9445**	ITM2B	Homo sapiens integral membrane protein 2B (ITM2B), mRNA.
**64101**	LRRC4	Homo sapiens leucine rich repeat containing 4 (LRRC4), mRNA.
**64757**	MTARC1	Homo sapiens mitochondrial amidoxime reducing component 1 (MTARC1),mRNA; nuclear gene for mitochondrial product.
**152006**	RNF38	Homo sapiens ring finger protein 38 (RNF38), transcript variant 1,mRNA.
**79023**	NUP37	Homo sapiens nucleoporin 37 (NUP37), mRNA.
**79077**	DCTPP1	Homo sapiens dCTP pyrophosphatase 1 (DCTPP1), transcript variant 1,mRNA.
**79727**	LIN28A	Homo sapiens lin-28 homolog A (LIN28A), mRNA.
**79750**	ZNF385D	Homo sapiens zinc finger protein 385D (ZNF385D), mRNA.
**79776**	ZFHX4	Homo sapiens zinc finger homeobox 4 (ZFHX4), mRNA.
**192669**	AGO3	Homo sapiens argonaute RISC catalytic component 3 (AGO3),transcript variant 1, mRNA.
**80014**	WWC2	Homo sapiens WW and C2 domain containing 2 (WWC2), mRNA.
**9662**	CEP135	Homo sapiens centrosomal protein 135 (CEP135), mRNA.
**114034**	TOE1	Homo sapiens target of EGR1, exonuclease (TOE1), mRNA.
**80306**	MED28	Homo sapiens mediator complex subunit 28 (MED28), mRNA.
**50964**	SOST	Homo sapiens sclerostin (SOST), mRNA.
**81563**	C1orf21	Homo sapiens chromosome 1 open reading frame 21 (C1orf21), mRNA.
**59283**	CACNG8	Homo sapiens calcium voltage-gated channel auxiliary subunit gamma8 (CACNG8), mRNA.
**84231**	TRAF7	Homo sapiens TNF receptor associated factor 7 (TRAF7), mRNA.
**84250**	SLF1	Homo sapiens SMC5-SMC6 complex localization factor 1 (SLF1), mRNA.
**84298**	LLPH	Homo sapiens LLP homolog, long-term synaptic facilitation factor(LLPH), mRNA.
**84750**	FUT10	Homo sapiens fucosyltransferase 10 (FUT10), mRNA.
**84919**	PPP1R15B	Homo sapiens protein phosphatase 1 regulatory subunit 15B(PPP1R15B), mRNA.
**85363**	TRIM5	Homo sapiens tripartite motif containing 5 (TRIM5), transcriptvariant alpha, mRNA.
**85364**	ZCCHC3	Homo sapiens zinc finger CCHC-type containing 3 (ZCCHC3), mRNA.
**5814**	PURB	Homo sapiens purine rich element binding protein B (PURB), mRNA.
**112939**	NACC1	Homo sapiens nucleus accumbens associated 1 (NACC1), mRNA.
**115825**	WDFY2	Homo sapiens WD repeat and FYVE domain containing 2 (WDFY2), mRNA.
**116449**	CLNK	Homo sapiens cytokine dependent hematopoietic cell linker (CLNK),mRNA.
**595**	CCND1	Homo sapiens cyclin D1 (CCND1), mRNA.
**116138**	KLHDC3	Homo sapiens kelch domain containing 3 (KLHDC3), transcript variant1, mRNA.
**80155**	NAA15	Homo sapiens N-alpha-acetyltransferase 15, NatA auxiliary subunit(NAA15), mRNA.
**90407**	TMEM41A	Homo sapiens transmembrane protein 41A (TMEM41A), mRNA.
**91894**	C11orf52	Homo sapiens chromosome 11 open reading frame 52 (C11orf52), mRNA.
**92092**	ZC3HAV1L	Homo sapiens zinc finger CCCH-type containing, antiviral 1 like(ZC3HAV1L), mRNA.
**140735**	DYNLL2	Homo sapiens dynein light chain LC8-type 2 (DYNLL2), mRNA.
**81035**	COLEC12	Homo sapiens collectin subfamily member 12 (COLEC12), mRNA.
**53354**	PANK1	Homo sapiens pantothenate kinase 1 (PANK1), transcript variantgamma, mRNA.
**90268**	OTULIN	Homo sapiens OTU deubiquitinase with linear linkage specificity(OTULIN), mRNA.
**91056**	AP5B1	Homo sapiens adaptor related protein complex 5 subunit beta 1(AP5B1), mRNA.

**113201**	GOLM2	Homo sapiens golgi membrane protein 2 (GOLM2), transcript variant1, mRNA.
**115207**	KCTD12	Homo sapiens potassium channel tetramerization domain containing 12(KCTD12), mRNA.
**129685**	TAF8	Homo sapiens TATA-box binding protein associated factor 8 (TAF8),mRNA.
**128637**	TBC1D20	Homo sapiens TBC1 domain family member 20 (TBC1D20), transcriptvariant 1, mRNA.
**140775**	SMCR8	Homo sapiens SMCR8-C9orf72 complex subunit (SMCR8), mRNA.
**93517**	SDR42E1	Homo sapiens short chain dehydrogenase/reductase family 42E, member1 (SDR42E1), mRNA.
**150737**	TTC30B	Homo sapiens tetratricopeptide repeat domain 30B (TTC30B), mRNA.
**148479**	PHF13	Homo sapiens PHD finger protein 13 (PHF13), mRNA.
**163702**	IFNLR1	Homo sapiens interferon lambda receptor 1 (IFNLR1), transcriptvariant 1, mRNA.
**283358**	B4GALNT3	Homo sapiens beta-1,4-N-acetyl-galactosaminyltransferase 3(B4GALNT3), mRNA.
**283373**	ANKRD52	Homo sapiens ankyrin repeat domain 52 (ANKRD52), mRNA.
**257194**	NEGR1	Homo sapiens neuronal growth regulator 1 (NEGR1), mRNA.
**153339**	TMEM167A	Homo sapiens transmembrane protein 167A (TMEM167A), mRNA.
**157638**	LRATD2	Homo sapiens LRAT domain containing 2 (LRATD2), transcript variant1, mRNA.
**221895**	JAZF1	Homo sapiens JAZF zinc finger 1 (JAZF1), mRNA.
**285636**	RIMOC1	Homo sapiens RAB7A interacting MON1-CCZ1 complex subunit 1(RIMOC1), mRNA.
**283459**	GATC	Homo sapiens glutamyl-tRNA amidotransferase subunit C (GATC),transcript variant 1, mRNA; nuclear gene for mitochondrial product.
**353132**	LCE1B	Homo sapiens late cornified envelope 1B (LCE1B), mRNA.
**339983**	NAT8L	Homo sapiens N-acetyltransferase 8 like (NAT8L), mRNA.
**340348**	TSPAN33	Homo sapiens tetraspanin 33 (TSPAN33), mRNA.
**146223**	CMTM4	Homo sapiens CKLF like MARVEL transmembrane domain containing 4(CMTM4), transcript variant 1, mRNA.
**149563**	SRARP	Homo sapiens steroid receptor associated and regulated protein(SRARP), mRNA.
**201595**	STT3B	Homo sapiens STT3 oligosaccharyltransferase complex catalyticsubunit B (STT3B), mRNA.
**8473**	OGT	Homo sapiens O-linked N-acetylglucosamine (GlcNAc) transferase(OGT), transcript variant 1, mRNA.
**221391**	OPN5	Homo sapiens opsin 5 (OPN5), transcript variant 1, mRNA.
**255488**	RNF144B	Homo sapiens ring finger protein 144B (RNF144B), mRNA.
**144108**	SPTY2D1	Homo sapiens SPT2 chromatin protein domain containing 1 (SPTY2D1),mRNA.
**64841**	GNPNAT1	Homo sapiens glucosamine-phosphate N-acetyltransferase 1 (GNPNAT1),mRNA.
**29841**	GRHL1	Homo sapiens grainyhead like transcription factor 1 (GRHL1), mRNA.
**55568**	GALNT10	Homo sapiens polypeptide N-acetylgalactosaminyltransferase 10(GALNT10), mRNA.
**286077**	FAM83H	Homo sapiens family with sequence similarity 83 member H (FAM83H),mRNA.
**374354**	NHLRC2	Homo sapiens NHL repeat containing 2 (NHLRC2), mRNA.
**283514**	SIAH3	Homo sapiens siah E3 ubiquitin protein ligase family member 3(SIAH3), mRNA.
**375035**	SFT2D2	Homo sapiens SFT2 domain containing 2 (SFT2D2), mRNA.
**145741**	C2CD4A	Homo sapiens C2 calcium dependent domain containing 4A (C2CD4A),mRNA.
**283377**	SPRYD4	Homo sapiens SPRY domain containing 4 (SPRYD4), mRNA.
**387640**	SKIDA1	Homo sapiens SKI/DACH domain containing 1 (SKIDA1), mRNA.

Supplementary Table 6. Reactome pathways overrepresented within miRNA targets up-regulated in T2^{high} vs T2^{low} patients.

#term ID	term description	observed gene count	background gene count	strength	signal	false discovery rate
HSA-2262752	Cellular responses to stress	79	747	0.35	0.82	5.49e-07
HSA-162582	Signal Transduction	189	2540	0.2	0.67	5.72e-07
HSA-74160	Gene expression (Transcription)	123	1476	0.25	0.69	1.81e-06
HSA-1280215	Cytokine Signaling in Immune system	71	706	0.33	0.73	6.25e-06
HSA-1643685	Disease	131	1702	0.21	0.59	2.95e-05
HSA-73857	RNA Polymerase II Transcription	107	1337	0.23	0.57	8.13e-05
HSA-2559585	Oncogene Induced Senescence	12	33	0.89	0.87	0.00014
HSA-5663202	Diseases of signal transduction by growth factor receptors and second messengers	47	430	0.36	0.63	0.00014
HSA-168256	Immune System	142	1979	0.18	0.51	0.00022
HSA-212436	Generic Transcription Pathway	97	1215	0.23	0.54	0.00022
HSA-2454202	Fc epsilon receptor (FCERI) signaling	22	131	0.55	0.69	0.00029
HSA-9006936	Signaling by TGFB family members	21	121	0.56	0.7	0.00029
HSA-5663205	Infectious disease	77	917	0.25	0.53	0.00044
HSA-597592	Post-translational protein modification	106	1405	0.2	0.49	0.00056
HSA-9006934	Signaling by Receptor Tyrosine Kinases	50	521	0.31	0.52	0.00094
HSA-453279	Mitotic G1 phase and G1/S transition	22	148	0.5	0.58	0.0012
HSA-69231	Cyclin D associated events in G1	12	47	0.73	0.65	0.0012
HSA-9661069	Defective binding of RB1 mutants to E2F1,(E2F2, E2F3)	8	17	1.0	0.7	0.0012
HSA-9679506	SARS-CoV Infections	42	411	0.33	0.52	0.0012
HSA-449147	Signaling by Interleukins	44	453	0.31	0.49	0.0018
HSA-983705	Signaling by the B Cell Receptor (BCR)	18	113	0.53	0.54	0.0024
HSA-170834	Signaling by TGF-beta Receptor Complex	16	92	0.56	0.55	0.0025
HSA-9006931	Signaling by Nuclear Receptors	30	265	0.38	0.49	0.0026
HSA-9682385	FLT3 signaling in disease	9	28	0.83	0.61	0.0026
HSA-69278	Cell Cycle, Mitotic	48	526	0.28	0.46	0.0027
HSA-1640170	Cell Cycle	56	658	0.25	0.44	0.0034
HSA-3108232	SUMO E3 ligases SUMOylate target proteins	22	166	0.45	0.5	0.0034
HSA-4420097	VEGFA-VEGFR2 Pathway	16	96	0.55	0.52	0.0034
HSA-5607764	CLEC7A (Dectin-1) signaling	16	96	0.55	0.52	0.0034
HSA-6785807	Interleukin-4 and Interleukin-13 signaling	17	107	0.53	0.52	0.0034
HSA-1280218	Adaptive Immune System	62	758	0.24	0.43	0.0036
HSA-8934593	Regulation of RUNX1 Expression and Activity	7	17	0.94	0.58	0.0043
HSA-2173793	Transcriptional activity of SMAD2/SMAD3:SMAD4 heterotrimer	11	50	0.67	0.52	0.0049
HSA-5621481	C-type lectin receptors (CLRs)	19	138	0.46	0.47	0.0054
HSA-9013423	RAC3 GTPase cycle	15	93	0.53	0.48	0.0061
HSA-109606	Intrinsic Pathway for Apoptosis	11	52	0.65	0.5	0.0062
HSA-1236382	Constitutive Signaling by Ligand-Responsive EGFR Cancer Variants	7	19	0.89	0.53	0.0063
HSA-8878159	Transcriptional regulation by RUNX3	15	94	0.53	0.47	0.0063
HSA-194315	Signaling by Rho GTPases	55	672	0.24	0.39	0.0069
HSA-8878166	Transcriptional regulation by RUNX2	17	119	0.48	0.46	0.0069
HSA-9716542	Signaling by Rho GTPases, Miro GTPases and RHOBTB3	56	688	0.24	0.39	0.0069

HSA-2173796	SMAD2/SMAD3:SMAD4 heterotrimer regulates transcription	9	36	0.72	0.5	0.0074
HSA-4615885	SUMOylation of DNA replication proteins	10	45	0.67	0.49	0.0074
HSA-4090294	SUMOylation of intracellular receptors	8	28	0.78	0.5	0.0076
HSA-114452	Activation of BH3-only proteins	8	29	0.77	0.48	0.0091
HSA-4551638	SUMOylation of chromatin organization proteins	11	57	0.61	0.45	0.0097
HSA-9607240	FLT3 Signaling	9	38	0.7	0.47	0.0097
HSA-9675126	Diseases of mitotic cell cycle	9	38	0.7	0.47	0.0097
HSA-2559583	Cellular Senescence	20	163	0.41	0.41	0.0101
HSA-1168372	Downstream signaling events of B Cell Receptor (BCR)	13	80	0.54	0.44	0.0104
HSA-157118	Signaling by NOTCH	23	204	0.38	0.4	0.0104
HSA-392499	Metabolism of proteins	125	1917	0.14	0.34	0.0104
HSA-5637810	Constitutive Signaling by EGFRvIII	6	15	0.93	0.49	0.0104
HSA-8939211	ESR-mediated signaling	22	190	0.39	0.41	0.0104
HSA-8986944	Transcriptional Regulation by MECP2	11	59	0.6	0.45	0.0105
HSA-9772573	Late SARS-CoV-2 Infection Events	12	70	0.56	0.44	0.0110
HSA-1169410	Antiviral mechanism by IFN-stimulated genes	13	82	0.52	0.42	0.0118
HSA-195258	RHO GTPase Effectors	29	292	0.32	0.38	0.0118
HSA-9665348	Signaling by ERBB2 ECD mutants	6	16	0.9	0.47	0.0118
HSA-9711123	Cellular response to chemical stress	22	195	0.38	0.39	0.0121
HSA-9694516	SARS-CoV-2 Infection	29	294	0.32	0.38	0.0123
HSA-9022707	MECP2 regulates transcription factors	4	5	1.23	0.47	0.0144
HSA-9754119	Drug-mediated inhibition of CDK4/CDK6 activity	4	5	1.23	0.47	0.0144
HSA-1169408	ISG15 antiviral mechanism	12	74	0.53	0.41	0.0152
HSA-9009391	Extra-nuclear estrogen signaling	12	75	0.53	0.4	0.0166
HSA-109581	Apoptosis	20	175	0.38	0.37	0.0171
HSA-1266738	Developmental Biology	78	1108	0.17	0.33	0.0171
HSA-4085377	SUMOylation of SUMOylation proteins	8	35	0.68	0.41	0.0178
HSA-9614085	FOXO-mediated transcription	11	65	0.55	0.4	0.0178
HSA-3371497	HSP90 chaperone cycle for steroid hormone receptors (SHR) in the presence of ligand	10	55	0.58	0.4	0.0186
HSA-109582	Hemostasis	48	607	0.22	0.33	0.0202
HSA-202403	TCR signaling	15	114	0.44	0.37	0.0202
HSA-2173789	TGF-beta receptor signaling activates SMADs	9	46	0.62	0.39	0.0202
HSA-5357801	Programmed Cell Death	22	206	0.35	0.35	0.0202
HSA-9703465	Signaling by FLT3 fusion proteins	6	19	0.82	0.41	0.0202
HSA-9694635	Translation of Structural Proteins	10	57	0.57	0.38	0.0218
HSA-400253	Circadian Clock	11	68	0.53	0.38	0.0219
HSA-4570464	SUMOylation of RNA binding proteins	9	47	0.61	0.38	0.0223
HSA-168928	DDX58/IFIH1-mediated induction of interferon-alpha/beta	12	80	0.5	0.37	0.0229
HSA-202424	Downstream TCR signaling	13	92	0.47	0.36	0.0232
HSA-5653656	Vesicle-mediated transport	51	666	0.21	0.31	0.0246
HSA-8866652	Synthesis of active ubiquitin: roles of E1 and E2 enzymes	7	29	0.71	0.38	0.0251
HSA-9013026	RHOB GTPase cycle	11	70	0.52	0.36	0.0253
HSA-6811442	Intra-Golgi and retrograde Golgi-to-ER traffic	21	201	0.34	0.33	0.0289
HSA-9615017	FOXO-mediated transcription of oxidative stress, metabolic and neuronal genes	7	30	0.69	0.37	0.0289
HSA-1227986	Signaling by ERBB2	9	50	0.58	0.35	0.0300

HSA-177929	Signaling by EGFR	9	50	0.58	0.35	0.0300
HSA-9006925	Intracellular signaling by second messengers	28	306	0.29	0.31	0.0311
HSA-1181150	Signaling by NODAL	6	22	0.76	0.37	0.0314
HSA-8941326	RUNX2 regulates bone development	7	31	0.68	0.36	0.0322
HSA-383280	Nuclear Receptor transcription pathway	9	52	0.56	0.34	0.0364
HSA-9735871	SARS-CoV-1 targets host intracellular signalling and regulatory pathways	5	15	0.85	0.36	0.0371
HSA-1257604	PIP3 activates AKT signaling	25	266	0.3	0.3	0.0375
HSA-9013404	RAC2 GTPase cycle	12	87	0.46	0.32	0.0375
HSA-5687128	MAPK6/MAPK4 signaling	12	88	0.46	0.32	0.0404
HSA-9679191	Potential therapeutics for SARS	13	101	0.43	0.31	0.0420
HSA-6783589	Interleukin-6 family signaling	6	24	0.72	0.34	0.0423
HSA-9634638	Estrogen-dependent nuclear events downstream of ESR-membrane signaling	6	24	0.72	0.34	0.0423
HSA-9648002	RAS processing	6	24	0.72	0.34	0.0423
HSA-9725370	Signaling by ALK fusions and activated point mutants	9	54	0.55	0.32	0.0423
HSA-9703648	Signaling by FLT3 ITD and TKD mutants	5	16	0.82	0.34	0.0427
HSA-1912422	Pre-NOTCH Expression and Processing	11	78	0.47	0.31	0.0433
HSA-199991	Membrane Trafficking	47	626	0.2	0.28	0.0433
HSA-8948747	Regulation of PTEN localization	4	9	0.97	0.35	0.0433
HSA-8951936	RUNX3 regulates p14-ARF	4	9	0.97	0.35	0.0433
HSA-9026519	Activated NTRK2 signals through RAS	4	9	0.97	0.35	0.0433
HSA-9675108	Nervous system development	44	575	0.21	0.28	0.0433
HSA-913531	Interferon Signaling	20	199	0.33	0.29	0.0447
HSA-446203	Asparagine N-linked glycosylation	27	304	0.27	0.28	0.0450
HSA-69206	G1/S Transition	15	130	0.39	0.3	0.0450
HSA-9006115	Signaling by NTRK2 (TRKB)	6	25	0.7	0.33	0.0450
HSA-933542	TRAF6 mediated NF-kB activation	6	25	0.7	0.33	0.0450
HSA-9664565	Signaling by ERBB2 KD Mutants	6	25	0.7	0.33	0.0450
HSA-114604	GPVI-mediated activation cascade	7	35	0.63	0.32	0.0470
HSA-2559580	Oxidative Stress Induced Senescence	12	92	0.44	0.3	0.0470
HSA-5683057	MAPK family signaling cascades	28	322	0.26	0.28	0.0470
HSA-6804757	Regulation of TP53 Degradation	7	35	0.63	0.32	0.0470
HSA-9012999	RHO GTPase cycle	36	449	0.23	0.27	0.0470
HSA-9755511	KEAP1-NFE2L2 pathway	13	105	0.42	0.3	0.0470
HSA-9758274	Regulation of NF-kappa B signaling	5	17	0.79	0.33	0.0470
HSA-165158	Activation of AKT2	3	4	1.2	0.34	0.0484
HSA-69895	Transcriptional activation of cell cycle inhibitor p21	3	4	1.2	0.34	0.0484
HSA-975871	MyD88 cascade initiated on plasma membrane	12	93	0.44	0.3	0.0484

Supplementary Table 7. Recent urinary proteomic studies (<10 years) in diseases not associated to the renal system (excluding lung diseases).			
**Disease**	**Organ/Tissue/system affected**	**Population group**	**References**
**Parkinson´s disease**	Brain	Adult	[1](2021)
**Alzheimer´s disease**	Brain	Adult	[2](2019)
**Rheumatoid arthritis**	Joint/Immune	Adult	[3](2014)
**Autism**	Brain	Children	[4](2021)<br>[5](2015)
**Coronary hearth disease**	Cardiovascular	Adult	[6](2019)
**Medulloblastoma**	Brain	Children	[7](2020)
**Acute coronary Syndrome**	Cardiovascular	Adult	[8](2017)
**Hypertension**	Cardiovascular	Adult	[9](2015)
**Myocardial infection**	Cardiovascular	Adult	[10](2019)
**Cardiovascular events**	Cardiovascular	Adult	[11](2015)
**Endometrial cancer**	Uterus/Endometrium	Adult	[12](2019)
**Hepatocellular carcinoma**	Liver	Adult	[13](2020)
**Gestational diabetes**	Cardiovascular	Adult	[14](2022)
**Rhabdomyolysis**	Skeletal muscle	Adult	[15](2022)
**Vitiligo**	Skin	Adult	[16](2022)
**Obesity**	Cardiovascular / Adipose tissue	Adult	[17](2019)
**Aging**	All tissues	Adult	[18](2021)<br>[19](2014)
**Pulmonary Embolism**	Cardiovascular/Lung	Adult	[20](2016)
**Multiple Sclerosis**	Brain/Spinal cord	Adult	[21](2015)
**Preeclampsia**	Cardiovascular	Adult	[22](2021)
**Osteoarthritis**	Joint	Adult	[23](2019)
**Kawasaki disease**	Cardiovascular	Children	[24](2013)

REFERENCES

1. Virreira Winter, S., et al., *Urinary proteome profiling for stratifying patients with familial Parkinson's disease.* EMBO Mol Med, 2021. **13**(3): p. e13257.
2. Watanabe, Y., et al., *Molecular Network Analysis of the Urinary Proteome of Alzheimer's Disease Patients.* Dement Geriatr Cogn Dis Extra, 2019. **9**(1): p. 53-65.
3. Kang, M.J., et al., *Urinary proteome profile predictive of disease activity in rheumatoid arthritis.* J Proteome Res, 2014. **13**(11): p. 5206-17.
4. Meng, W., Y. Huan, and Y. Gao, *Urinary proteome profiling for children with autism using data-independent acquisition proteomics.* Transl Pediatr, 2021. **10**(7): p. 1765-1778.
5. Suganya, V., A. Geetha, and S. Sujatha, *Urine proteome analysis to evaluate protein biomarkers in children with autism.* Clin Chim Acta, 2015. **450**: p. 210-9.
6. Sun, H., et al., *Differential urinary proteins to diagnose coronary heart disease based on iTRAQ quantitative proteomics.* Anal Bioanal Chem, 2019. **411**(11): p. 2273-2282.
7. Hao, X., et al., *Urinary protein biomarkers for pediatric medulloblastoma.* J Proteomics, 2020. **225**: p. 103832.
8. Htun, N.M., et al., *Prediction of acute coronary syndromes by urinary proteome analysis.* PLoS One, 2017. **12**(3): p. e0172036.
9. Zhang, Z.Y., et al., *Urinary Proteome and Systolic Blood Pressure as Predictors of 5-Year Cardiovascular and Cardiac Outcomes in a General Population.* Hypertension, 2015. **66**(1): p. 52-60.

10. Zou, L., et al., *Differential urinary proteomics analysis of myocardial infarction using iTRAQ quantification.* Mol Med Rep, 2019. **19**(5): p. 3972-3988.
11. Brown, C.E., et al., *Urinary proteomic biomarkers to predict cardiovascular events.* Proteomics Clin Appl, 2015. **9**(5-6): p. 610-7.
12. Kacirova, M., et al., *Differential Urinary Proteomic Analysis of Endometrial Cancer.* Physiol Res, 2019. **68**(Suppl 4): p. S483-S490.
13. Zhao, Y., et al., *Identification of noninvasive diagnostic biomarkers for hepatocellular carcinoma by urinary proteomics.* J Proteomics, 2020. **225**: p. 103780.
14. Wang, X., et al., *Urinary proteomic analysis during pregnancy and its potential application in early prediction of gestational diabetes mellitus and spontaneous abortion.* Ann Transl Med, 2022. **10**(13): p. 736.
15. Carneiro, A., et al., *Urine proteomics as a non-invasive approach to monitor exertional rhabdomyolysis during military training.* J Proteomics, 2022. **258**: p. 104498.
16. Qian, Y.T., et al., *Urinary Proteomics Analysis of Active Vitiligo Patients: Biomarkers for Steroid Treatment Efficacy Prediction and Monitoring.* Front Mol Biosci, 2022. **9**: p. 761562.
17. Benabdelkamel, H., et al., *A Proteomics-Based Approach Reveals Differential Regulation of Urine Proteins between Metabolically Healthy and Unhealthy Obese Patients.* Int J Mol Sci, 2019. **20**(19).
18. Yu, Y., et al., *Protein signatures from blood plasma and urine suggest changes in vascular function and IL-12 signaling in elderly with a history of chronic diseases compared with an age-matched healthy cohort.* Geroscience, 2021. **43**(2): p. 593-606.
19. Bakun, M., et al., *Urine proteomes of healthy aging humans reveal extracellular matrix (ECM) alterations and immune system dysfunction.* Age (Dordr), 2014. **36**(1): p. 299-311.
20. D'Andrea, V., et al., *Chievitz' juxtaparotid organ, free from cancer.* Ann Ital Chir, 2015. **86**: p. 503-7.
21. Nielsen, H.H., et al., *The Urine Proteome Profile Is Different in Neuromyelitis Optica Compared to Multiple Sclerosis: A Clinical Proteome Study.* PLoS One, 2015. **10**(10): p. e0139659.
22. Bujold, E., et al., *Proteomic Analysis of Maternal Urine for the Early Detection of Preeclampsia and Fetal Growth Restriction.* J Clin Med, 2021. **10**(20).
23. Xiao, K., et al., *Urine Proteomics Profiling and Functional Characterization of Knee Osteoarthritis Using iTRAQ Technology.* Horm Metab Res, 2019. **51**(11): p. 735-740.
24. Kentsis, A., et al., *Urine proteomics for discovery of improved diagnostic markers of Kawasaki disease.* EMBO Mol Med, 2013. **5**(2): p. 210-20.
